# A deep learning-based algorithm for 2-D cell segmentation in microscopy images

**DOI:** 10.1186/s12859-018-2375-z

**Published:** 2018-10-03

**Authors:** Yousef Al-Kofahi, Alla Zaltsman, Robert Graves, Will Marshall, Mirabela Rusu

**Affiliations:** 1 GE Global Research, One Research Circle, Niskayuna, 12309 NY USA; 2 GE Healthcare, 1040 12th Ave NW, Issaquah, 98027 WA USA; 3 Present address: Department of Radiology, Stanford University, 1201 Welch Rd, Stanford, 94305 CA USA

**Keywords:** Microscopy images, 2-D cells segmentation, Deep learning, Watershed segmentation

## Abstract

**Background:**

Automatic and reliable characterization of cells in cell cultures is key to several applications such as cancer research and drug discovery. Given the recent advances in light microscopy and the need for accurate and high-throughput analysis of cells, automated algorithms have been developed for segmenting and analyzing the cells in microscopy images. Nevertheless, accurate, generic and robust whole-cell segmentation is still a persisting need to precisely quantify its morphological properties, phenotypes and sub-cellular dynamics.

**Results:**

We present a single-channel whole cell segmentation algorithm. We use markers that stain the whole cell, but with less staining in the nucleus, and without using a separate nuclear stain. We show the utility of our approach in microscopy images of cell cultures in a wide variety of conditions. Our algorithm uses a deep learning approach to learn and predict locations of the cells and their nuclei, and combines that with thresholding and watershed-based segmentation. We trained and validated our approach using different sets of images, containing cells stained with various markers and imaged at different magnifications. Our approach achieved a 86% similarity to ground truth segmentation when identifying and separating cells.

**Conclusions:**

The proposed algorithm is able to automatically segment cells from single channel images using a variety of markers and magnifications.

**Electronic supplementary material:**

The online version of this article (10.1186/s12859-018-2375-z) contains supplementary material, which is available to authorized users.

## Background

The cell is the basic structural, functional and biological unit in all living organisms. The ability to image, extract and study cells and their sub-cellular compartments is essential to various research areas. Examples include cellular dynamics characterization in normal and pathologic conditions [1] as well as drug discovery where it is important to assess the efficacy of different drug treatments [2]. Recent advancements in high-resolution fluorescent microscopy paved the way for detailed visualization of the cells and their sub-cellular structures [3]. These advancements have been accompanied by the evolution of computing capabilities and the development of novel techniques in computer vision and machine learning for image segmentation and classification [4].

Automatic analysis of 2-D cellular images enables accurate and high-throughput cell quantification and provides reproducible information. Such quantification may enable researchers to address different biological problems instead of relying on the subjective and time-consuming interpretation of human experts.

**Fig. 1:**
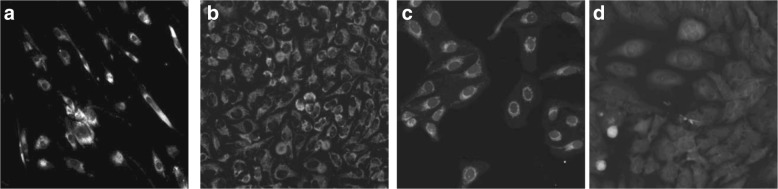
Various channel markers allow the visualization of cells **a**,**b** dsRed, **c** TexasRed, and **d** Cy5. A large variability exists in the appearance of cells, based on the utilized marker and magnification

Often, the term cell segmentation has been used to refer to segmentation of the cell nuclei as opposed to segmenting the entire cell body including the cytoplasm. In this work, we focused on whole cell segmentation in 2D microscopy images where the cytoplasm appears bright, the background is dark, while the nucleus has little or no staining. Our approach involves 1) detecting the cells, 2) separating touching cells and 3) segmenting sub-cellular compartments (i.e. nucleus vs. cytoplasm). Segmenting and separating cell boundaries is a challenging task. Unlike the nuclei that are blob-like similar-sized structures, the cytoplasm shows significant variation in shape and size (Fig. 1). Moreover, touching cells can have weak boundary gradients rendering difficult the separation task.

Over the past decades, several algorithms have been proposed to segmenting cells in 2-D images [4, 5]. Some approaches rely only on one-channel images but perform the segmentation of only the cell nuclei as opposed to segment the cytoplasm. For instance, watershed-based segmentation [6, 7] and levelset methods [8, 9] have been used to separate touching and overlapping nuclei. Other techniques included morphology-based segmentation [10], which assumes a blob-like shape for the cell nucleus, or blob-based detection that initializes a graph-based method [11]. Active contours models and snake algorithms have also been utilized, e.g. [12].

On the other hand, fewer algorithms perform single-channel whole cell segmentation. For instance, machine learning algorithms were used for pixel-based classification and segmentation of cells in bright-field / phase contrast images, e.g. [13, 14]. Moreover, an iterative threshold-based approaches was used in [15]. Those algorithms were evaluated on images with uniform cell appearance and did not show evidence of segmenting images with large variations in cell appearance, as seen in the examples in Fig. 1.

Other approaches rely on using two-channel images. First they segment the nuclei using nuclear stain channel, and then use the nuclei as seeds to segment the whole cell based on a second channel of a cell body/cytoplasm stain (i.e. showing the entire cell), e.g. [16–18]. More recently, deep learning techniques [19] have also been applied for the segmentation of cell nuclei and cytoplasm [20–23]. Van Valen et al. [21] used a two-channel approach with both phase contrast images and fluorescent (nuclear) images to segment the mammalian cell cytoplasm. The authors utilized both channels simultaneously when segmenting the cell cytoplasm. Recent deep-learning based methods have also been focusing on differentiating sub-cellular compartments/organelles, including nuclei, cytoplasm, fibers, etc., using multiple channels [22, 23]. Some methods were used to identify cells of different classes either using multiple channels, including one showing a nuclear marker and one showing the cytoplasm, [21, 24]. However, no actual segmentation of the cell boundary was performed [24]. On the other hand, a convolutional neural network approach was used to segment brightfield images of cells in [25]. However, clustered cells were not separated.

The recent interest in segmenting and tracking cells has prompted the organization of three Cell Tracking Challenges [20]. The goal of the challenge was to track the cells over time, as cells are moving or dividing. Having multiple frames may either help segment individual cells, as multiple instances of the same data is available, but at the same time requires tracking trajectories and divisions which are also challenging. Few of the images included in the challenge dataset have the same characteristics as the images included in our test datasets, i.e. hyperintense cytoplasm and hypointense nuclei and background, the segmentation task in our dataset is rather different.

Given the limited number of channels available in most multiplexed fluorescent microscopes, it is very often desirable to maximize the number of channels used for analytical (discovery) biomarkers to better study different biological phenomena. Moreover, often researchers prefer not to use nuclear markers that might be toxic to the cells, especially in live cell imaging.

The use of different markers and different cell types results in high variability in the cell shape and appearance between different images or between the different cells in the same image. For example, Fig. 1 shows five sample cell images using different markers at two different magnifications. These images were arbitrarily selected to show the variability in the appearance of the stains within the cytoplasm for different cells and experimental conditions. Furthermore, treatment of cells with compounds, such as drugs, leads to dramatic changes in the number of cells and their cellular morphology. Hence, it is very challenging to design generic algorithms that can be easily applied and extended to different and new types of markers and cells. Therefore, there is a persisting need to develop automated and generic algorithms for 2-D cell segmentation.

Segmenting cells in images that show the nuclei and background as hypointense regions while the cytoplasm is hyperintense (Fig. 1) is a challenging task for various reasons. First, microscopy images including ours usually show large variability in the data: 1) appearance and morphology of the nuclei and cells varies greatly between experiments and within experiments, especially drug titration treatments, 2) different markers are used to show various organelles or regions in the cytoplasm, and 3) the images can be acquired at different magnifications. Second, the edges between nuclei and background may be very subtle or even not visible at all in some images (e.g. Fig. 1b), thereby being able to segment the cytoplasm, which encompasses the nuclei can be a daunting task, especially in images showing tightly-packed cells.

In this work, we present an algorithm for automated segmentation of the whole cells, including nuclei and the cytoplasm, in 2-D cellular images. The approach was specifically designed to be robust for images that show hyperintense cytoplasm and nuclei with little or no staining. We evaluated the approach on a wide range of cell markers, drug treatment conditions and magnifications.

Our work brings the following contributions to the state-of-the-art methods in 2-D cell segmentation:

1. We present a deep learning-based framework to provide per-pixel probabilities for nuclei, cytoplasm and background using a single channel image.
2. We present an efficient algorithm that applies blob detection and shape-based watershed to detect the individual nuclei from the nucleus prediction map
3. We present a seeded-watershed algorithm for individual cell segmentation using the cell prediction map as well as the segmented nuclei.

## Methods

**Fig. 2:**
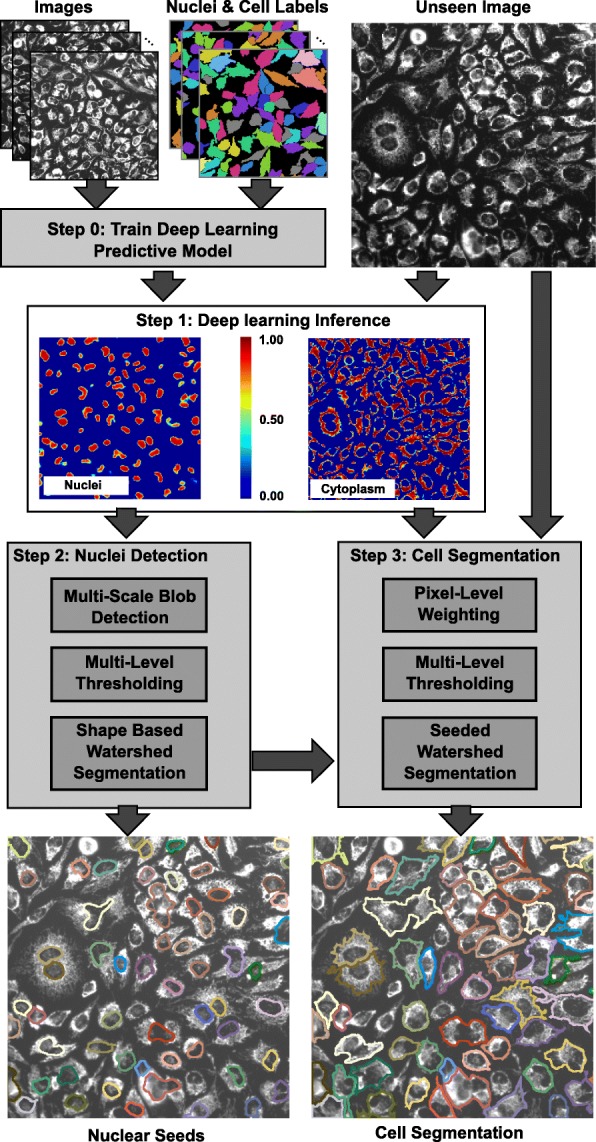
Overview of the 2-D cell segmentation algorithm. Labeled images are used as training set for deep learning. The unseen images are passed through the inference engine to create the probability maps for the nuclear seeds and cytoplasm. Multiple steps are required for the nuclear seed prediction and the cell segmentation

We introduce a single-channel cell segmentation algorithm that uses a cytoplasm marker that usually shows hypointense nuclear regions and hyperintense cellular regions. Our method does not rely on cell nuclei or membrane markers for the cell segmentation. The algorithm requires an offline step to train the deep network model (step 0) to predict cells and nuclei based on one channel images. Given an unseen image to be segmented, the algorithm proceeds in 3 steps as illustrated in Fig. 2: Step 1) Deep learning-based prediction of nuclei and cytoplasm, Step 2) Nuclei seeds detection and Step 3) Seed-based cell segmentation. The details about each step is provided below.

### Image preprocessing

As preprocessing steps prior to training and inference, we corrected the uneven illumination by suppressing the image background via top-hat filtering with a kernel size of 200x200 pixels. Also, to account for the differences in image magnification (and thus pixel size), images are down-sampled to be (approximately) at 10x magnification (e.g. pixel size = 0.65 *μm*x0.65 *μm*).

### Step 0) Train deep learning predictive model

Our deep learning framework used the MXNet library [26] and a UNet-like architecture [27] to compute pixel-level predictions for multiple classes. More specifically, our model is trained using image patches of 160x160 pixels to predict 3 different labels: nuclei, cytoplasm and background. Each label has its own predominant characteristics (see examples in Fig. 1). For instance, nuclei have low-intensity signal compared to the cell body. Often, the intensity range for the nucleus is close to that of the image background. On the other hand, the texture patterns of the brighter cell body, i.e. cytoplasm, vary from one image into another based on the used marker and its concentration. From the input image patch, a series of 5 convolution and pooling steps are applied in the contracting path as detailed in Table 1. The convolution kernel size is 3x3 and the numbers of filters for the 5 layers are 32, 64, 128, 128 and 256. Thus, the lowest layer results with 5x5 images. We found this sequence of filters to be stable and to provide good results. In addition, it is computationally less expensive than using a sequence with 1024 filters at the bottom of the contracting path. The contracting path is followed by an expanding path that includes a series of deconvolution layers (i.e. transposed convolution). Furthermore, we added three layers of dropout regularization to our architecture to reduce model over-fitting on the training data. Notice that our architecture is asymmetric, with minor differences in the number of filters and convolution steps between the contracting and expanding paths as can be seen in Table 1. Our motivation for choosing such architecture was to optimize the network to better solve our problem.

**Table 1:** The used U-net architecture

L#	Type	Size	Output	L#	Type	Size	Output
1	Input		1,160,160	17	Concatenate		256,20,20
2	Convolution	32 filters	32,160,160	18	Dropout	50%	256,20,20
3	Max pool	2 stride 2x2	32,80,80	19	Convolution	128 filters	128,20,20
4	Convolution	64 filters	64,80,80	20	Deconvolution	2 stride, 128x2x2	128,40,40
5	Max pool	2 stride 2x2	64,40,40	21	Convolution	128 filters	128,40,40
6	Convolution	128 filters	128,40,40	22	Concatenate		192,40,40
7	Max pool	2 stride 2x2	128,20,20	23	Dropout	50%	192,40,40
8	Convolution	128 filters	128,20,20	24	Convolution	128 filters	128,40,40
9	Max pool	2 stride 2x2	128,10,10	25	Deconvolution	2 stride, 128x2x2	128,80,80
10	Convolution	256 filters	256,10,10	26	Convolution	128 filters	128,80,80
11	Max pool	2 stride 2x2	256,5,5	27	Concatenate		160,80,80
12	Dropout	50%	256,5,5	28	Concatenate		160,80,80
13	Deconvolution	2 stride, 256x2x2	256,10,10	29	Convolution	64 filters	64,80,80
14	Convolution	128 filters	128,10,10	30	Deconvolution	2 stride, 128x2x2	64,160,160
15	Deconvolution	2 stride, 128x2x2	128,20,20	31	Convolution	64 filters	64,160,160
16	Convolution	128 filters	128,20,20	32	Output		3,160,160

To set the number of epochs, we carried out multiple experiments in which our model was iteratively trained for 30–50 epochs. Then, we found that using 30 epochs to be sufficient for our model to converge. In each epoch, the goal is to estimate the network weights such that a loss function is minimized. More specifically, let *l*_*n*_∈{0,1}, *l*_*c*_∈{0,1} and *l*_*b*_∈{0,1} respectively denote the nuclei, cytoplasm and background labels in the training dataset, and let *p*_*n*_∈ [ 0,1], *p*_*c*_∈ [ 0,1] and *p*_*b*_∈ [ 0,1] be the predictions of the deep learning architecture for the nuclei, cytoplasm and background respectively. Then, the loss function is defined as the root mean square deviation (RSMD) of the prediction and label. In addition, it includes a constrain for the relationship between the different labels as follows:

1 $$\begin{array}{*{20}l} f(x)=& w_{n} * RMSD(p_{n}, l_{n}) + w_{c}*RMSD(p_{c},l_{c})+\\ &w_{b} * RMSD(p_{b},l_{b})+w*RMSD(l_{n}+l_{c}+l_{b},1)  \end{array} $$

where *w*_*n*_, *w*_*c*_, *w*_*b*_ and *w* represent the weights associated with the nuclei, cytoplasm and the background. In our tests, the weights were equal with one. The training input images were divided into overlapping patches of 176x176 pixels, with an overlap of 16 pixels from each side. Therefore, only the internal 160x160 pixels are unique for each patch and were used to train our model. The training data is augmented by rotating the original patches by 90 degrees. Other parameters included the batch size, which was set to 32 in order to achieve good accuracy while being memory efficient, and the learning rate, which was initiated to 0.001.

### Step 1) deep learning inference

**Fig. 3:**
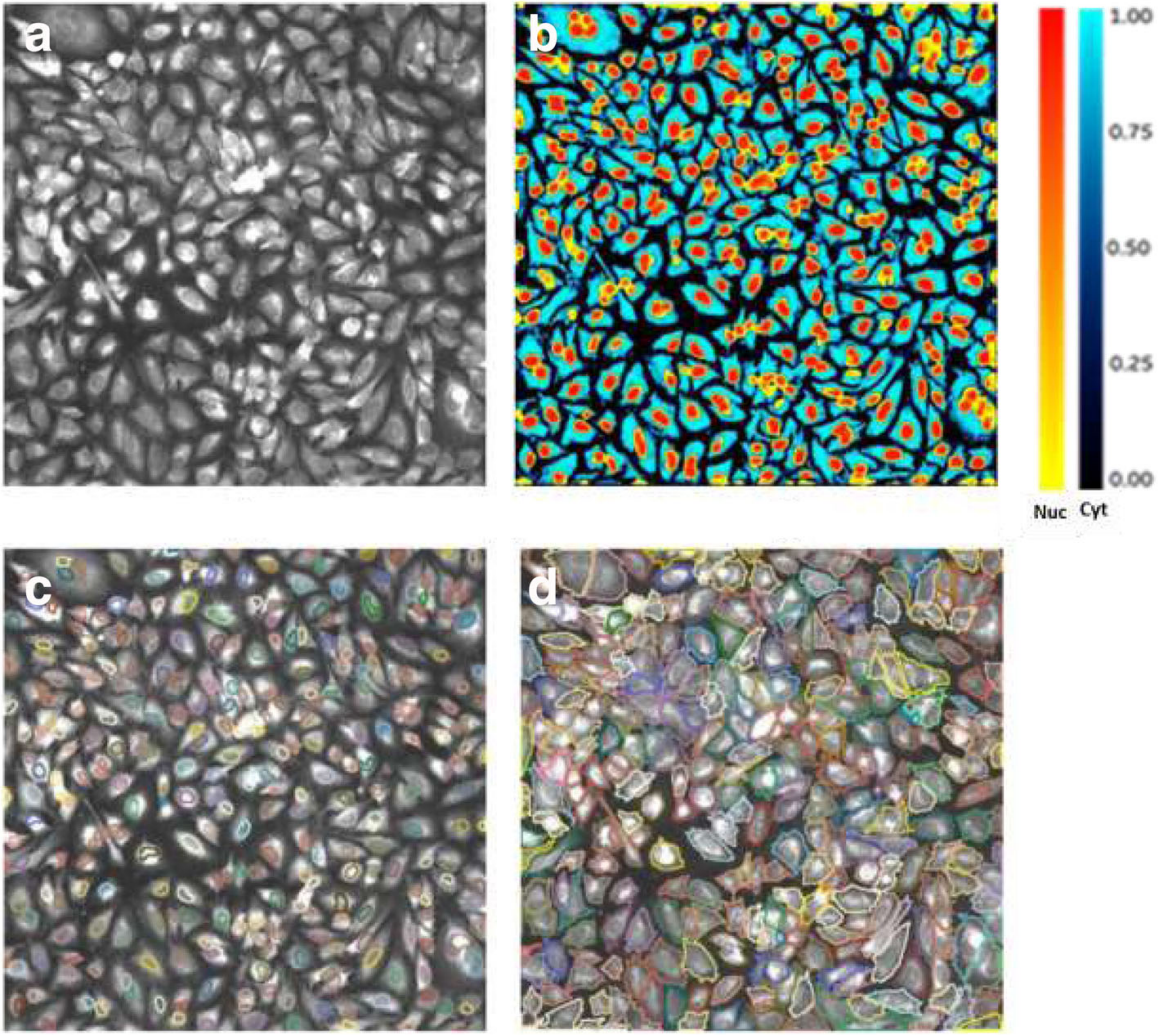
Prediction and segmentation step-by-step outcome. **a** Input image. **b** Nuclei (Yellow-Red) and Cells (Blue-Cyan) prediction map. **c** Segmented Nuclei (seeds), **d** Segmented Cells

Following image preprocessing, the unseen images are divided into 176x176 patches, which are used to create a probability map with a range [0,1] for the nucleus, cytoplasm and background. Once the prediction is completed, the predicted patches are stitched together to build the prediction of the full image. Figure 3b shows an example of Nuclei (Yellow-Red) and Cells (Blue-Cyan) prediction map.

### Step 2) nuclei seed detection

The Nuclei prediction map shows larger probabilities at the locations of nuclei inside the cells. Yet, these nuclei need to be individually segmented as they will serve as seeds to segment the entire cells. In images with sparse cells, simple image thresholding at 0.5 may be sufficient to extract a nuclear mask and identify the independent nuclei. However, this approach is sensitive to false positives and may result in large connected components for touching nuclei of adjacent cells.

Therefore, we propose a nuclei seed detection step that extracts and segments the individual nuclei seeds in the image. Given the nuclei prediction map, a multi-level Laplacian of Gaussian (LoG) blob detector [28] is applied to enhance regions containing blob-like nuclei at multiple scales. The LoG blob detector takes into consideration the expected morphology as well as the intensity profile of the nucleus. The rationale behind applying the LoG at multiple scale is to detect nuclei with different sizes. Next, we extracted the binary nuclear mask, which is achieved through an automated multi-level Otsu thresholding [29]. First, the selected threshold depended on the sensitivity parameter that was used. In our experiments, we set the sensitivity to 60, which was converted into using the third threshold (out of five) as the final threshold to define image background, hypointense nuclei (blobs) and hyperintense nuclei (blobs). Then, we combine all detected nuclei to create a binary image.

The binary mask separates the nuclei from the background. However, touching nuclei may end up forming large connected components. Using these multi-nuclei connected components as seeds for cell segmentation will result with merging adjacent cells. Hence, the last final step of nuclei segmentation delineates the individual nuclei using a shape-based watershed approach. This step starts by computing the inverse distance transform of the binary nuclear mask such that the value at each pixel equals its Euclidean distance from the background. Then, an extended h-minima transform [30] is applied on the distance transform. This starts by applying the H-minima transform at a level *h* to suppress all regional minima in an image whose depth is less than a value *h*. Then it extracts the regional minima of the resulting image. The parameter *h* is set by the user and its default value is 3 *μm*. In the last step, a seeded watershed transform is applied on the inverse distance transform and uses the regional minima extracted in the previous step as seeds. Figure 3c shows a nuclear seed segmentation example for the input image in panel (a) and the nuclei prediction map in panel (b).

### Step 3) cell segmentation

The segmentation of the cells is achieved in multiple steps (Fig. 2) and uses as inputs the cell marker image and the cytoplasm prediction map as obtained from the deep learning step. The cytoplasm prediction map (Cyan-Blue heat map in Fig. 3b) alone was not sufficient to segment the cells, especially when seeking to split touching cells. To ensure the robustness of our approach, we used the segmented nuclei (see the Yellow-Red heat map in Fig. 3b) as seeds for the cell segmentation.

Next, we combined the transformed version of the intensity image with the cell probability maps to enhance the cells by simply multiplying the two images. The transformation of the intensity image consists of applying a Gaussian filter (for simple denoising) followed by intensity scaling and then conversion to log space. Then, we determine the background based on a three-level Otsu thresholding. This step utilizes the number of detected nuclei and the expected cell area to compute the total expected cell area. More specifically, the optimal Otsu threshold is selected to be equal or between the three thresholds such that it results in an area estimated to be the closest to the expected area.

The identified background label, along with the segmented nuclei, are used in the seeded watershed segmentation of the cell marker image. This approach allows for the identification and separation of cells. For each nucleus, the approach will identify a corresponding cell. The approach is robust to a wide variety of stains, cell types, drug treatments and image magnifications.

### Evaluation of the classification results

A 10-fold cross-validation was performed to assess the receiver operating curve (ROC), the Area under the curve (AUC) and the accuracy (ACC) of the nuclei and cell predictions. The cross-validation was performed using the 108 independent images (datasets 1–5 in Table 2). In each cross-validation fold, the images were split into three non-overlapping sets of images: training set (80%), validation set (10%) and a test set (10%).

**Table 2:** Summary of datasets used for the training and testing of the deep learning framework

Data set	Training or testing			Image no	Marker channel
	Experiment 1	Experiment 2	Experiment 3		
1	Training	Training, Testing	Training	22	Green-dsRed, Red-Cy5
2	Training	Training, Testing	Training	12	Green-dsRed
3	Training	Training, Testing	Training	24	Red-Cy5
4	Training	Training, Testing	Training	30	TexasRed-TexasRed
5	10 Training, 10 Testing	Training, Testing	Training	20	Green-dsRed, Red-Cy5
6			Testing	15	TexasRed-TexasRed

Each images has a 2048 x 2048 resolution

For each fold, we assess the ACC to show the values of the mean and standard deviation when assessing either cells or nuclei. Note that the AUC and ACC are computed on binary masks, assuming that all nuclei are one label and respectively all cytoplasm are another label. To obtain the AUC, we thresholded the prediction maps resulting from each deep-learning testing step (without post-processing), using threshold values ranging between 0 and 1 which cover the entire span of predicted values. The thresholding allows us to obtain the sensitivity and specificity values at each level, thus enabling the plotting of the AUC curves. The ACC values are computed using a 0.50 threshold value, which were similarly applied to the predictions maps resulting from the deep-learning testing.

### Segmentation similarity metric

We assess the quality of the automated segmentation by comparing it to the ground truth or reference segmentation. For the images with one or just a few segmented objects, simple binary similarity metrics, e.g. Dice overlap ratio, may be sufficient to assess the quality of the segmentation. However, simple binary measures are not sufficient for cell segmentation given the large number of segmented cells (e.g. hundreds). Therefore, we introduced here a cell segmentation similarity metric and use it to compare our segmentation results to the ground truth.

Let *I*_*R*_ be the reference segmentation image and *I*_*T*_ be the automated target segmentation image. Let the set of labels in the reference and target segmentation be defined as *R*={*r*_1_,*r*_2_,...,*r*_*N*_} and *T*={*t*_1_,*t*_2_,...,*t*_*M*_} respectively. Then, we define a one-to-any mapping *F*:*R*→*T* such that each label in the reference segmentation *r*_*i*_∈*R* is mapped to the corresponding zero or more labels that overlaps within T. The set of zero or more labels in T that are mapped to *r*_*i*_ is denoted as $\phantom {\dot {i}\!}T^{r_{i}}$. Also, define a bijective mapping *P*:*R*→*T* such that each label in the reference segmentation corresponds to one label in the target segmentation, and vice-versa. The set of labels that meet this one-to-one relationship is denoted as $P_{T}^{R}$. Then, the segmentation similarity function SM(R,T) is defined as follows:

2 $$ SM(R,T)\! =\! k \left(\frac{1}{N} \sum\limits_{i=1}^{N} \max_{t_{j} \in T^{r_{i}}}\frac{2|r_{i} \cap t_{j}|}{|r_{i}|+|t_{j}|} \right) +(1-k)\left(\frac{2\left|P_{T}^{R}\right|}{N+M} \right)  $$

where 0≤*k*≤1.0 is a weighting factor and | | represents the cardinality of the set. In this work, we empirically set *k*=0.6. In the equation above, the first term computes the average maximum overlap between each label in the reference segmentation *r*_*i*_∈*R* with the corresponding labels (if any) in the target segmentation $\phantom {\dot {i}\!}T^{r_{i}} \in T$ while the second term computes the ratio of true positive labels to all the labels.

### Experimental design

To evaluate the performance of our approach, we used a dataset containing images of five cellular assays in 96-well microplates (will refer to them as plates for simplicity) acquired using GE’s IN Cell Analyzer systems. We used different types of cell lines including Hela, fibroblasts, HEPG2 and U2OS. In addition, we used different types of markers. For examples a eGFP bound to a tandem FYVE domain construct, was used in two of the plates (first and fifth). In those plates, compounds were added to the cells to deplete intracellular levels of PI(3)P which caused a redistribution of the eGFP signal from punctate endosomes to a more diffuse cytosolic localization. In two of the other plates (second and fourth), we used MitoTracker Red (Thermo Fisher), which stains mitochondria in live cells and its accumulation is dependent on cell membrane potential. In the last (third) plate, the used marker was a proprietary dye reagent from the GE Cytiva Cell Health kit that localizes to the mitochondria.

The different plates were scanned at different magnifications including 10x (pixel size: 0.65 *μm* x 0.65 *μm*) and 20x (pixel size: 0.325 *μm* x 0.325 *μm*). Regardless of the magnification, each image dimension is 2048x2048 pixels. In addition, different fluorescent markers were used to identify cell body or the cytoplasm (Fig. 1). Only a small subset of the wells in each plate (e.g. one or two rows) were used in our experiments, with a total of 123 images. Table 2 lists the data sets that were used in the different experiments for either training or testing our algorithm.

### Ground truth

A set of ground truth segmentations is needed to train our deep learning model as well as to evaluate the goodness of our segmentation results. Ideally, a human expert should create such ground truth segmentation. However, this is a time-consuming process, especially since each image may contain several hundreds of cells. To overcome this limitation, we trained our algorithm using the automatic segmentations obtained when using a two-channel method that utilizes both the nuclear and cell marker channels. We refer to these segmentations as the 2-channel automated (sub-optimal) cell segmentation. Specifically, we first detected the nuclei based on the nuclear (e.g. DAPI) channel using the algorithm described in Step 2 and utilized those nuclei as seeds in Step 3 to segment the cells in the cell marker channel. Segmentation parameters were iteratively optimized and the results were reviewed by experts for feedback and to confirm on segmentation quality.

In addition to the automatically generated ground truth segmentations, a small set of 10 images were semi-automatically segmented by an expert and used in one of our experiments to validate our automated segmentation results as will be explained later (we will refer to them as ground truth segmentations). The expert biologist used Cell Profiler [31] to generate an initial sub-optimal segmentation and further refine and edit the segmentation results by splitting, merging, adding and removing cells.

## Results

Given the automated (sub-optimal) and semi-automated ground truth segmentations, we performed three experiments. For each experiment, the automated two-channel sub-optimal ground truth segmentations were used for training. However, the dataset was divided between training and testing differently. Furthermore, we optimized the network architecture in the first experiment and then used the same architecture in the two other experiments.

### Experiment 1

**Fig. 4:**
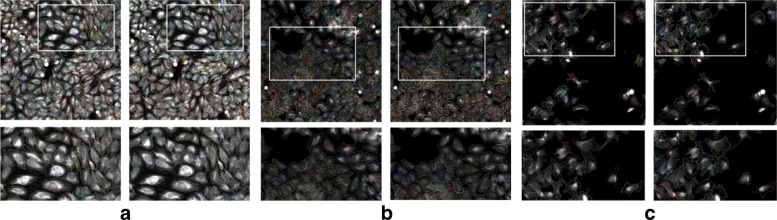
Examples of segmentation results from Experiment 1. **a**-**c** Different stains and cell cultures. Right column: segmentation results using our deep learning-based approach. Left column: semi-automated ground truth segmentation. Bottom row shows close-ups of the area in the white box. Different cell contours are shown in different colors

In Experiment 1, we used the datasets 1–5 in Table 2, which include 108 images. The 10 images with semi-automated ground truth segmentations were utilized for testing while the remaining 98 images were divided into training (88) and validation (10). Examples of segmentation results are shown in Fig. 4. Each example (a-c) shows the automated segmentation results obtained using our proposed algorithm (left column) as well as the semi-automated ground truth segmentation provided by the expert (right column). Although it is apparent that the two segmentations are not identical, they show high similarity when visually comparing them.

**Fig. 5:**
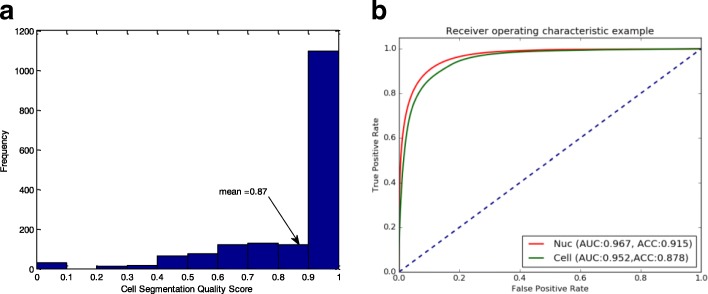
**a** Experiment 1: Histogram of the cell-level quality scores, for a total of 1666 segmented cells. The overall (average) quality score is ∼0.87. **b** Experiment 2: Receiver Operating Curve for a 10-fold cross validation the proposed approach

To assess the accuracy of the proposed segmentation algorithm, we used our similarity metric (SM) to compare the results of the proposed method to the semi-automated ground truth as well as the automated two-channel segmentation. The ground truth segmentations of the 10 test images contained 1666 cells. Then, segmentation quality was computed for each individual cell by comparing it to the corresponding ground truth segmentation of the same cell. Figure 5a shows a histogram of the cell-level quality measures, which showed ∼0.87 overall average cell segmentation quality when compared to the semi-automated ground truth segmentation. Moreover, when comparing our segmentation results to the automated two-channel segmentation results, we found an average score of ∼0.86. Notice that the average quality score between automated two-channel segmentation and the ground truth segmentation was ∼0.93.

In addition to the cell-level segmentation quality scores, we also computed the image-level quality scores by simply averaging the cell scores at the image level. The details of the image-level segmentation quality assessment are given in Table 3. We compared our proposed deep learning segmentation results to the automated two-channel segmentation and the semi-automated ground truth segmentation. The average image-level quality scores were at 0.85 and 0.86 respectively. Clearly, the image level score is slightly lower than the overall cell-level score because one of the images (no 8) showed lower quality than the other images. That image shows actin fiber staining in which the cells are more challenging to segment. These fibers appear elongated and in most cases uniform across the cell. Therefore, no significant different can be seen between the nucleus and the cytoplasm of the cell. This image is also shown in Fig. 1e). To get a better insight on the quality of our segmentation, we compared the two-channel segmentation, which was used for training, to the semi-automated ground truth, and that resulted with an average image-level score of 0.93. This is slightly higher than that of our deep-learning based approach, but on the expense on using an additional channel staining the cell nuclei (e.g. DAPI) in the segmentation.

**Table 3:** Experiment 1: Image level segmentation comparisons

Image ID	Deep learning to two-channel similarity	Deep learning to ground truth similarity	Two-channel to ground truth similarity
1	0.88	0.90	0.94
2	0.86	0.85	0.94
3	0.89	0.91	0.94
4	0.92	0.91	0.91
5	0.88	0.90	0.93
6	0.83	0.84	0.94
7	0.76	0.80	0.87
8	0.72	0.72	0.96
9	0.83	0.86	0.94
10	0.89	0.90	0.95
Avg.	0.85	0.86	0.93

### Experiment 2

In this experiment, we performed 5-fold cross-validation, which used the 108 independent images in the datasets 1–5 (Table 2), with 80% of the images used for the training set, and 10% for each of the validation and test sets. The ROC curve (Fig. 5b) suggests a good performance in identifying both nuclei and cells, with an AUC larger than 0.95 and ACC of 0.915 and 0.878 for the nuclei and cell respectively.

Furthermore, Table 4 shows a summary of the segmentation accuracy for the different datasets. The overall accuracy for the four datasets was computed to be ∼0.84. We found it more intuitive to summarize the results at the dataset level because we wanted to study the performance of the algorithm given the variability we see between the different datasets. For instance, the segmentation quality score for the third datasets was significantly lower than the others (∼0.62). Such results may be attributed to the higher variability in the cell shape and appearance in that dataset and therefore, were more difficult to segment. In this table, the number of detected cells is provided to give more details about the dataset size, but it is not be directly related to the accuracy.

**Table 4:** Experiment 2 - Summary of segmentation similarity - SM (accuracy) for the 10-fold cross-validation

Data set	Number of detected cells	Segmentation SM (accuracy)
1,5	6378	0.86 ±0.14
2	2162	0.62 ±0.09
3	2735	0.91 ±0.12
4	7961	0.85 ±0.17
	Total cell no = 19236	Avg. Accuracy = 0.84 ±0.14

### Experiment 3

The third experiment included datasets 1–5 when training the network, and split the 108 images into 98 images for training and 10 images for validation. Unlike experiment 1, we tested our model using dataset 6, which contains 15 images (Table 2), and is a completely independent experiment than datasets 1–5. Since no semi-automated ground truth segmentation was available for the dataset 6, we generated two-channel sub-optimal segmentations and used them as ground truth for the purpose of computing the segmentation similarity (i.e. accuracy).

**Fig. 6:**
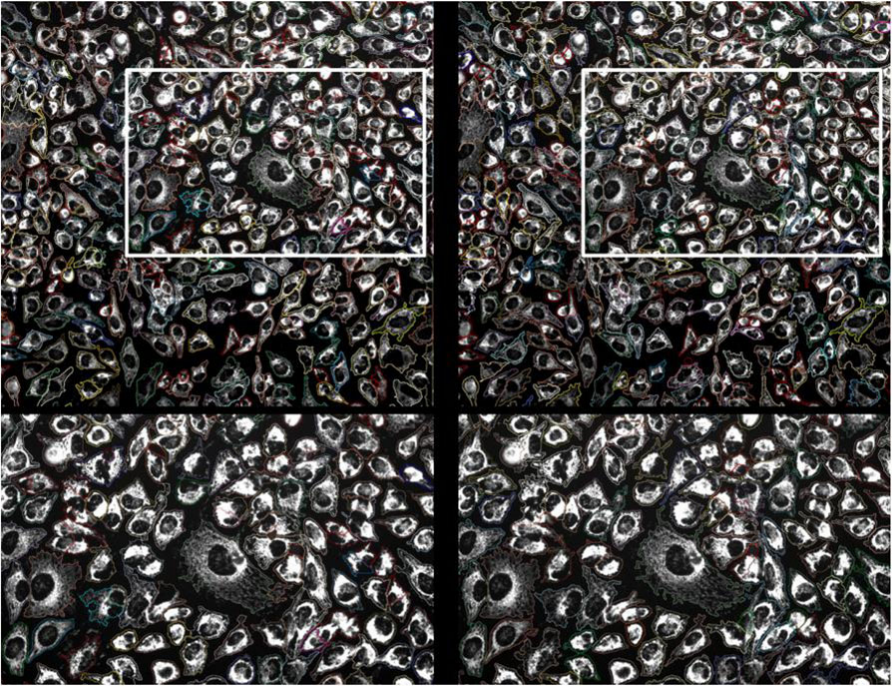
Segmentation examples from Experiment 3. Right column: segmentation results using our deep learning-based approach. Left column: semi-automated ground truth segmentation. Bottom row shows close-ups of the area in the white box. Different cell contours are shown in different colors

Similar to experiment 1, we first computed the overall cell-level segmentation quality scores, which was found to be at ∼0.84. Then, we computed the image-level segmentation quality scores by averaging the cell-level scores for each image. The detailed list of scores is given in Table 5. Most of the scores ranged between 0.8 and 0.9 with an average similarity score of 0.84. An example is shown in Fig. 6 comparing our single-channel deep learning-based segmentation to the two-channel segmentation for one image.

**Table 5:** Experiment 3: Image level segmentation comparisons

Image ID	Deep learning to two-channel similarity	Image ID	Deep learning to two-channel similarity
1	0.88	9	0.84
2	0.83	10	0.80
3	0.82	11	0.86
4	0.81	12	0.85
5	0.84	13	0.85
6	0.76	14	0.88
7	0.85	15	0.79
8	0.87	Avg.	0.84

### Implementation and processing time

The presented deep learning algorithm was implemented in python using the MXNet library [26], while the nuclei and cell segmentations were developed using C++, ITK [32] and Python. The deep learning approach was trained on an Amazon cloud environment (AWS) based on Ubuntu Linux using an NVIDIA graphics card Tesla K80. The training of the deep learning predictor takes 11–13 min per epoch, for ∼6h per fold. Applying our approach on an unseen image takes 4–6s/image.

## Discussion

Despite our very encouraging segmentation results, a few aspects of our algorithms could be improved. These areas will be the focus of our future. First, we will make further modifications to the existing deep learning algorithm, which include slight optimization to the network architecture, and optimization of the loss function. Second, we will explore new network architectures that will result in better predictions and will reduce the risk of post-processing errors. Third, we will investigate and test additional data augmentation strategies, which include generating synthetic data. Fourth, we will work on improving the speed and accuracy of the post-processing algorithm. Furthermore, a possible improvement to our algorithm will be to predict the locations of cell boundaries using the CNN model, and therefore to eliminate or, at least, to reduce the number of post-processing steps. In one of our early experiments, we tried to define a cell boundary class using the cell-to-cell borders in the ground truth segmentation. Unfortunately, that resulted with poor prediction of the cell boundaries. That poor performance could be attributed to the imperfect nature of our ground truth segmentation results. In future work, we may investigate adding higher weights in the loss function on pixels close to the cell boundaries.

## Conclusions

We presented an algorithm for 2-D cell segmentation in microscopy images using a single channel/marker. Given the significant variability in cell appearance that resulted from using different stains and different cell types, achieving robust cell segmentation results via traditional image analysis or machine learning approaches may require carefully engineered/handcrafted image features that capture intensity and morphological properties of the cells. To overcome this limitation, we trained a deep convolutional neural network (CNN) that uses a cascade of layers of non-linear processing units for feature extraction and transformation to form a hierarchy from low-level to high-level features. The deep CNN was then used to predict locations of cells and nuclei in test images. Our deep learning prediction was followed with a few post-processing steps to help generate the final cell segmentation mask. Given the accuracy of our predictions, we relied on traditional image analysis techniques for post-processing such as LoG blob detection and watershed transforms since they were efficient, computationally attractive and produced sufficiently good results. Our segmentation results were assessed both qualitatively (by an expert) and quantitatively. The quantitative assessment was performed by computing a similarity measure between each segmented image and the corresponding semi-automated ground truth segmentation and/or the automated two-channel segmentation. In general, the accuracies are slightly higher when comparing our results to the semi-automated ground truth than to the automated two-channel segmentation. That is expected because the automatic two-step segmentation algorithm had errors that were manually corrected in the semi-automated ground truth segmentation. Our proposed algorithm did not reproduce some of those errors and therefore, it was closer to the semi-automated ground truth segmentation. Both the qualitative and quantitative results showed that we could use a single channel (cell marker) to obtain a segmentation that is comparable to that obtained when using two-channels (i.e. with the addition of a nuclear channel).

## Additional file

Additional file 1 Sample images and results. Sample datasets used in this paper (# 1 and #5 in table 2). The dataset includes input images of both dsRed and Cy5 channels and the corresponding cell segmentation. (ZIP 245,472 kb)

## Abbreviations

LOG: Laplacian of Gaussian
RSMD: Root mean square deviation
SM: Similarity function
