# Supplementary figures and images for: A deep learning-based algorithm for 2-D cell segmentation in microscopy images
Source: BMC Bioinformatics. 2018 Oct 3;19:365. doi: 10.1186/s12859-018-2375-z (PMC6171227; doi:10.1186/s12859-018-2375-z)

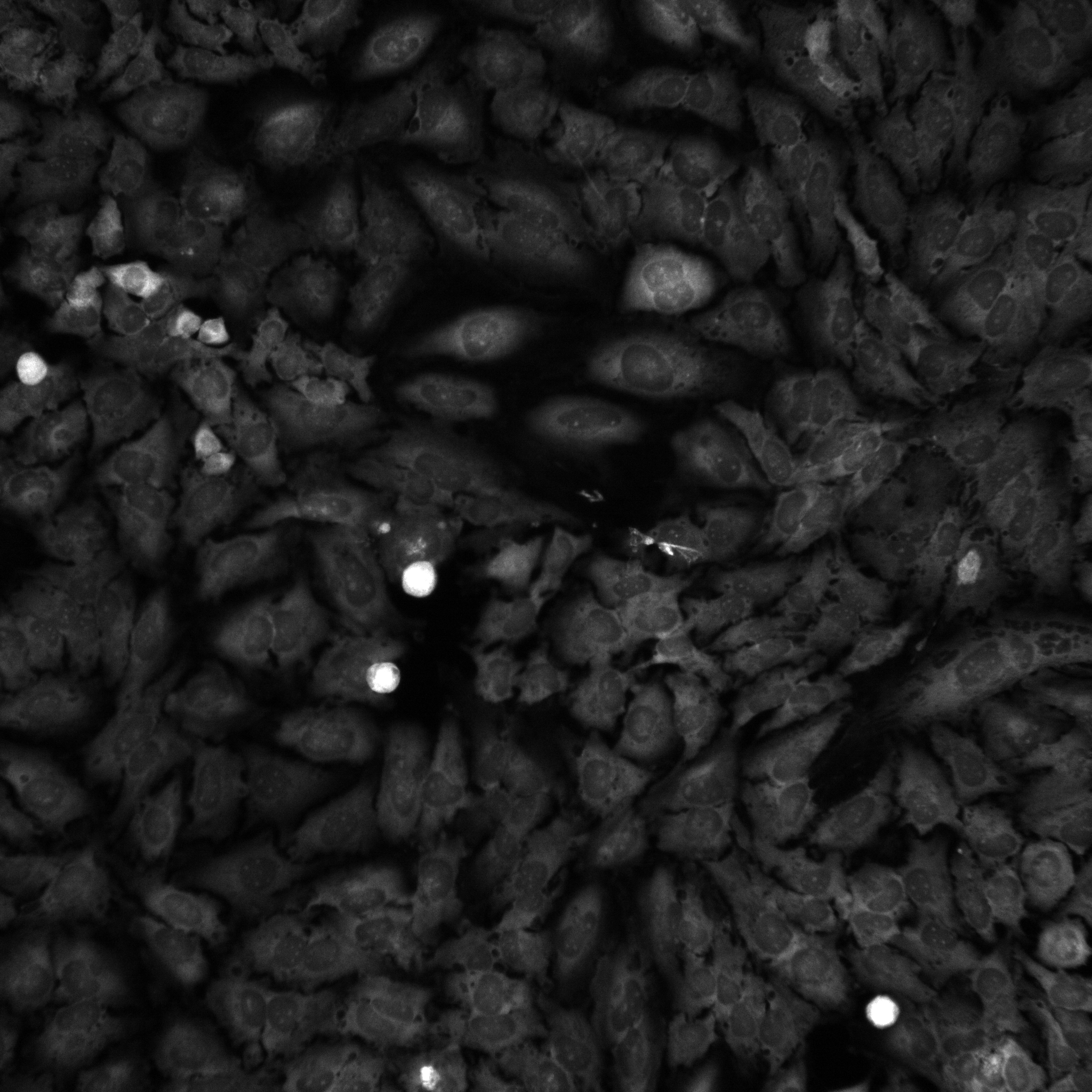

Supplement: Supplementary file 1 — Sample images and results. Sample datasets used in this paper (# 1 and #5 in table 2). The dataset includes input images of both dsRed and Cy5 channels and the corresponding cell segmentation. (ZIP 245,472 kb) [file 12859_2018_2375_MOESM1_ESM.zip › FYVE Hela 1/A - 1(fld 1 wv Green - dsRed).tif]

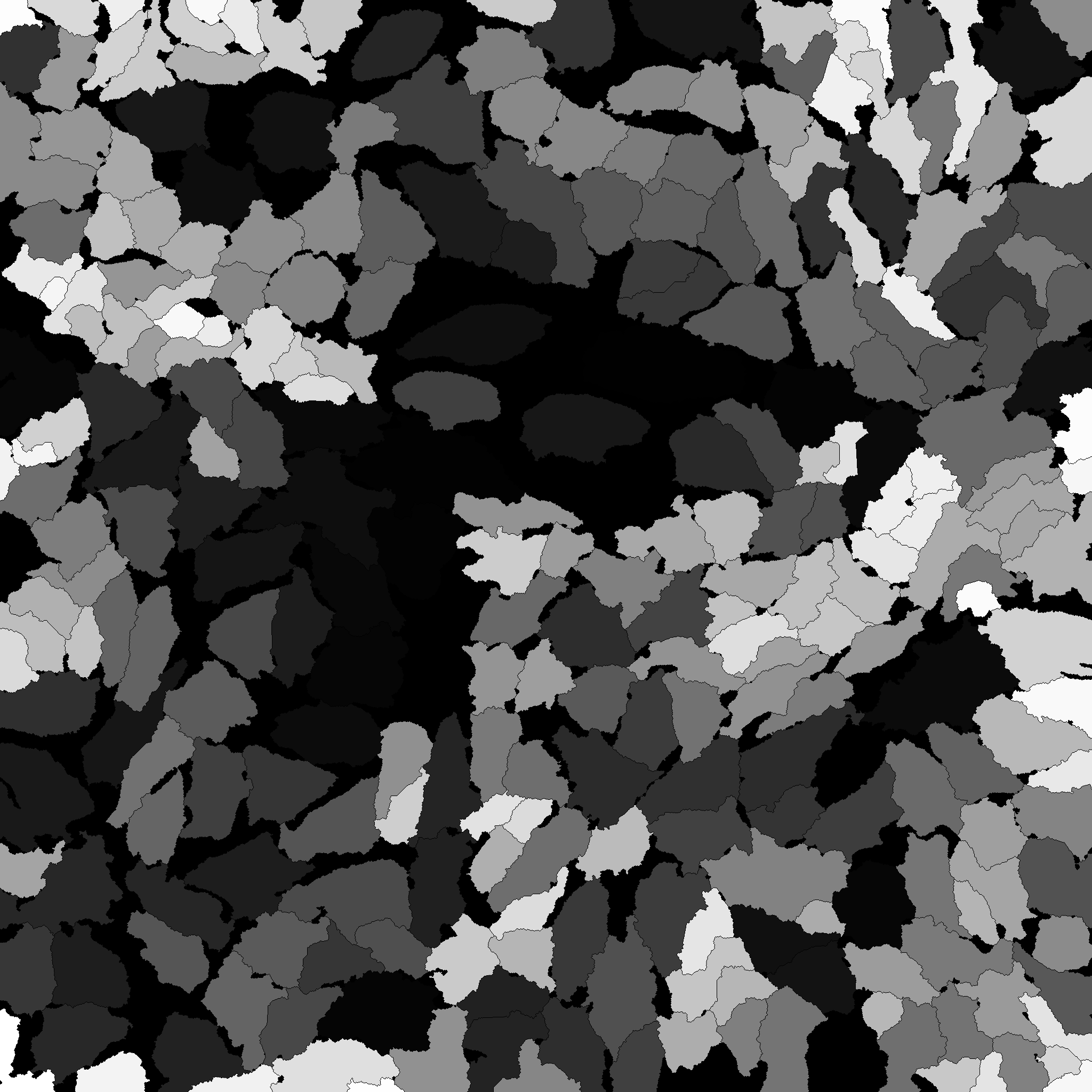

Supplement: Supplementary file 1 — Sample images and results. Sample datasets used in this paper (# 1 and #5 in table 2). The dataset includes input images of both dsRed and Cy5 channels and the corresponding cell segmentation. (ZIP 245,472 kb) [file 12859_2018_2375_MOESM1_ESM.zip › FYVE Hela 1/A - 1(fld 1 wv Green - dsRed)_cellseg_label.tif]

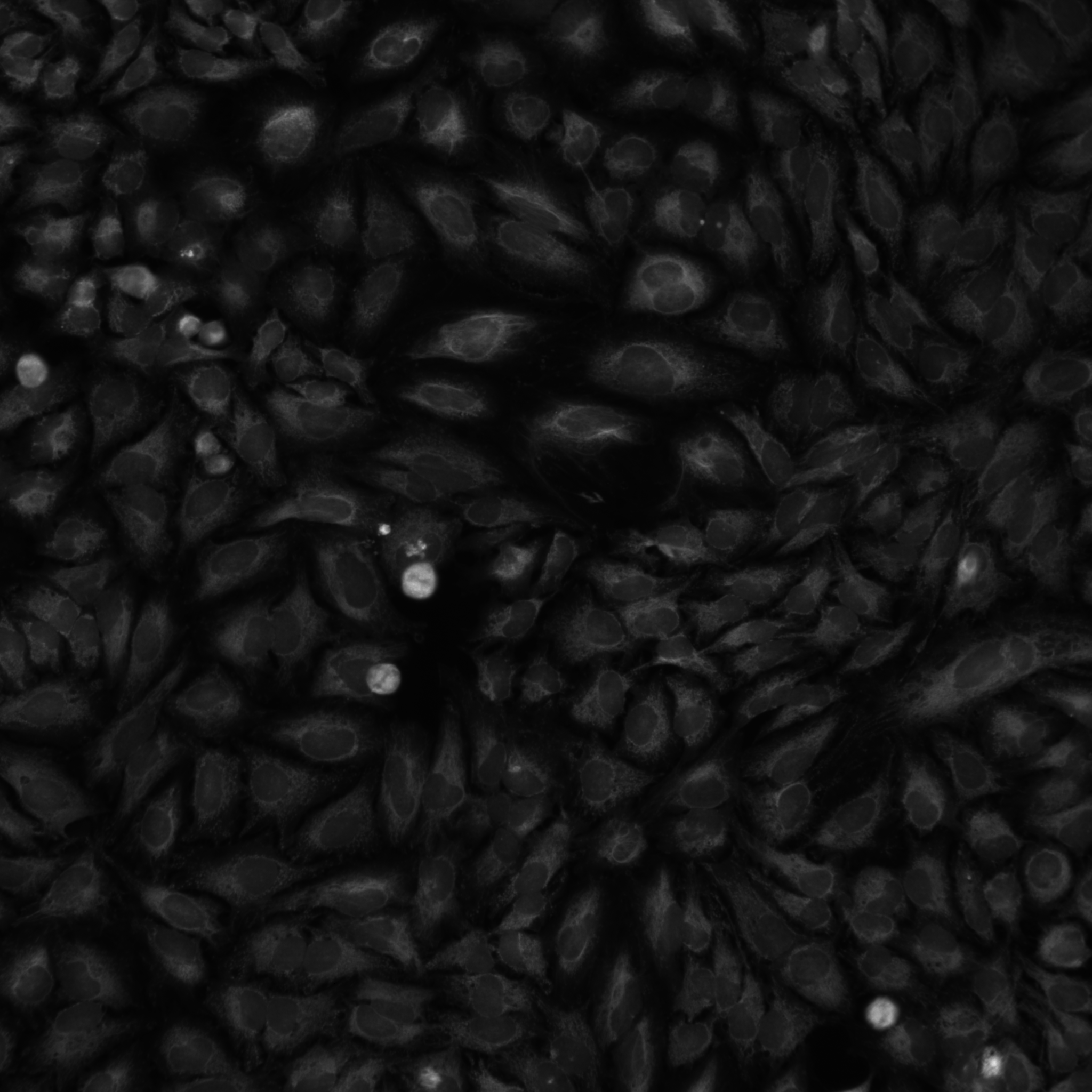

Supplement: Supplementary file 1 — Sample images and results. Sample datasets used in this paper (# 1 and #5 in table 2). The dataset includes input images of both dsRed and Cy5 channels and the corresponding cell segmentation. (ZIP 245,472 kb) [file 12859_2018_2375_MOESM1_ESM.zip › FYVE Hela 1/A - 1(fld 1 wv Red - Cy5).tif]

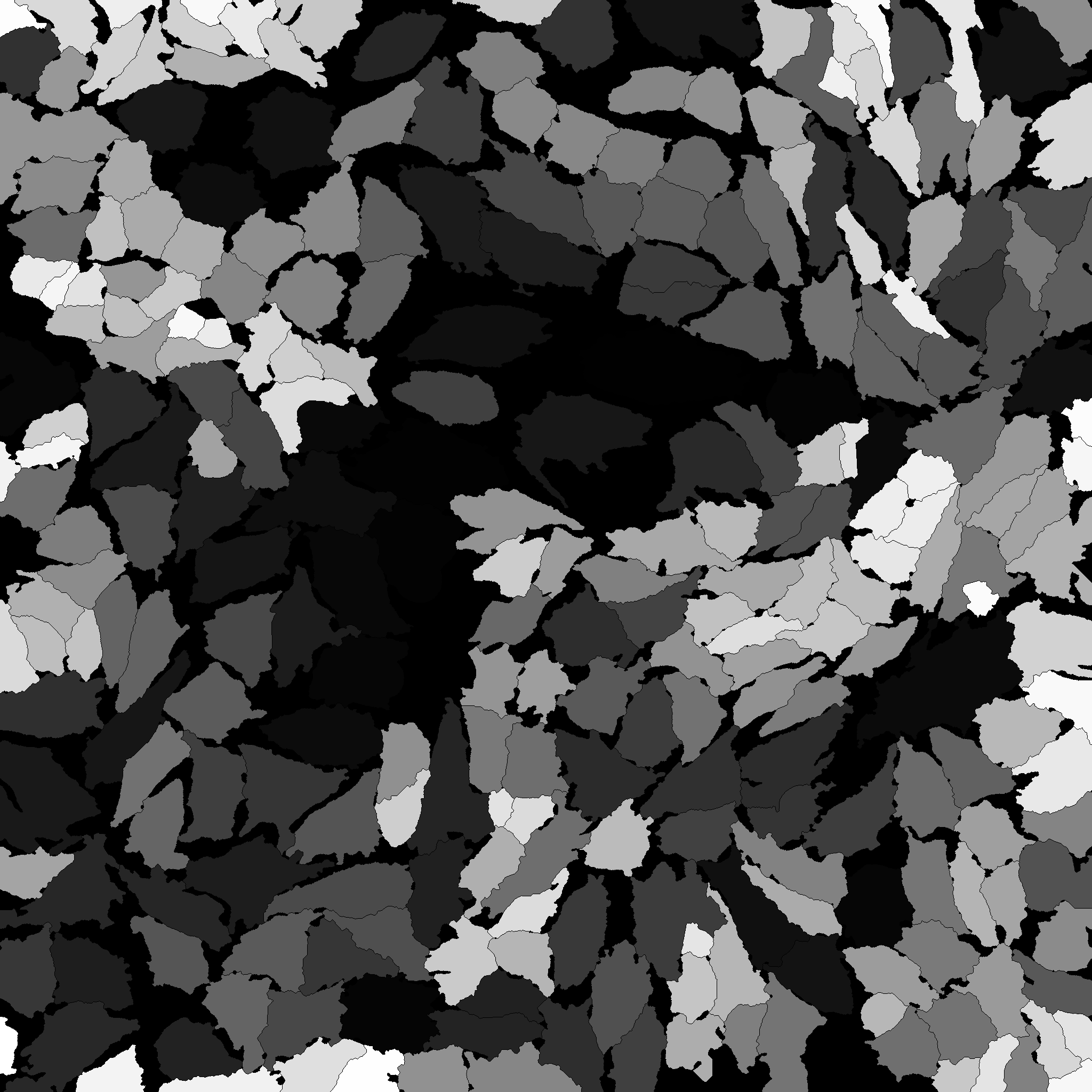

Supplement: Supplementary file 1 — Sample images and results. Sample datasets used in this paper (# 1 and #5 in table 2). The dataset includes input images of both dsRed and Cy5 channels and the corresponding cell segmentation. (ZIP 245,472 kb) [file 12859_2018_2375_MOESM1_ESM.zip › FYVE Hela 1/A - 1(fld 1 wv Red - Cy5)_cellseg_label.tif]

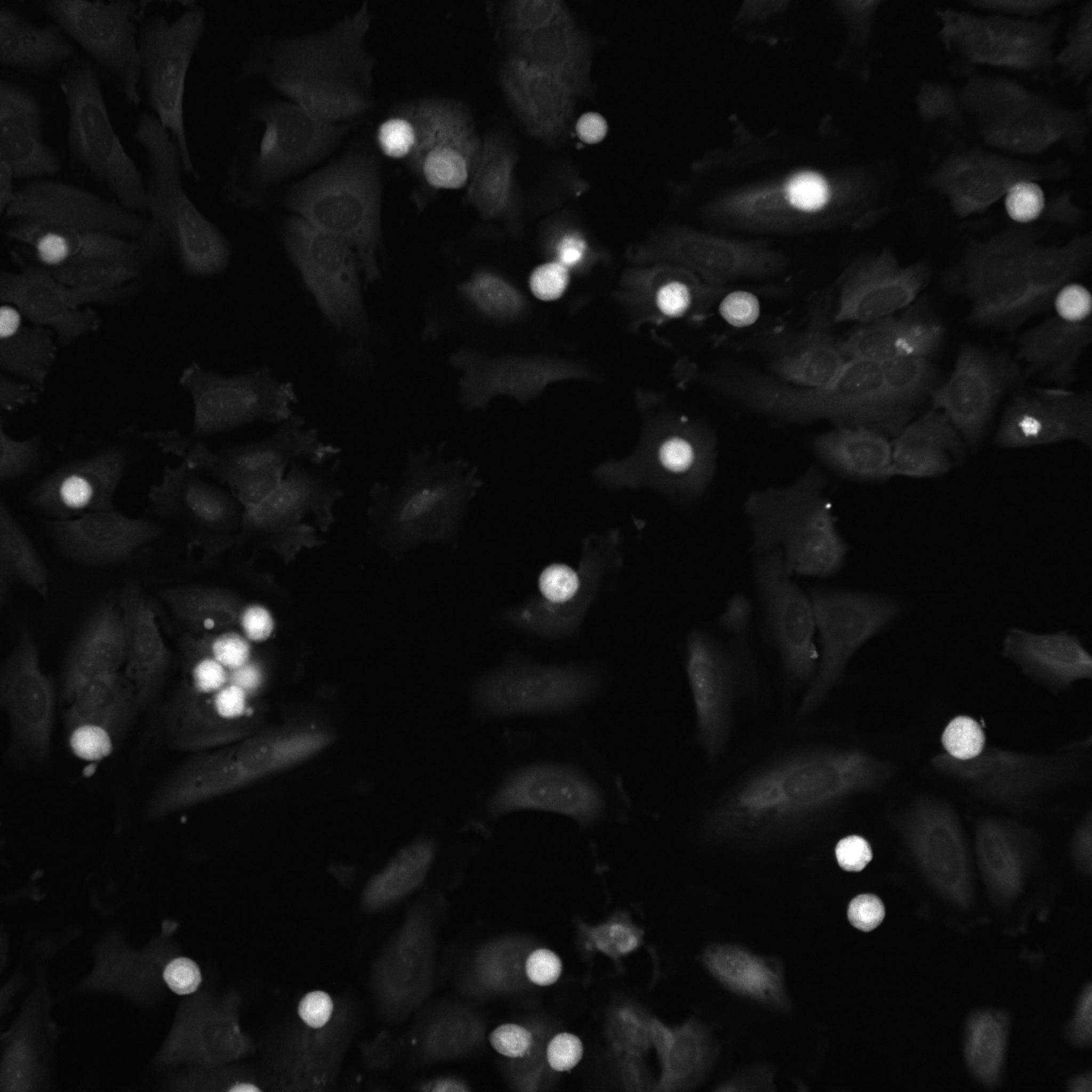

Supplement: Supplementary file 1 — Sample images and results. Sample datasets used in this paper (# 1 and #5 in table 2). The dataset includes input images of both dsRed and Cy5 channels and the corresponding cell segmentation. (ZIP 245,472 kb) [file 12859_2018_2375_MOESM1_ESM.zip › FYVE Hela 1/A - 10(fld 1 wv Green - dsRed).tif]

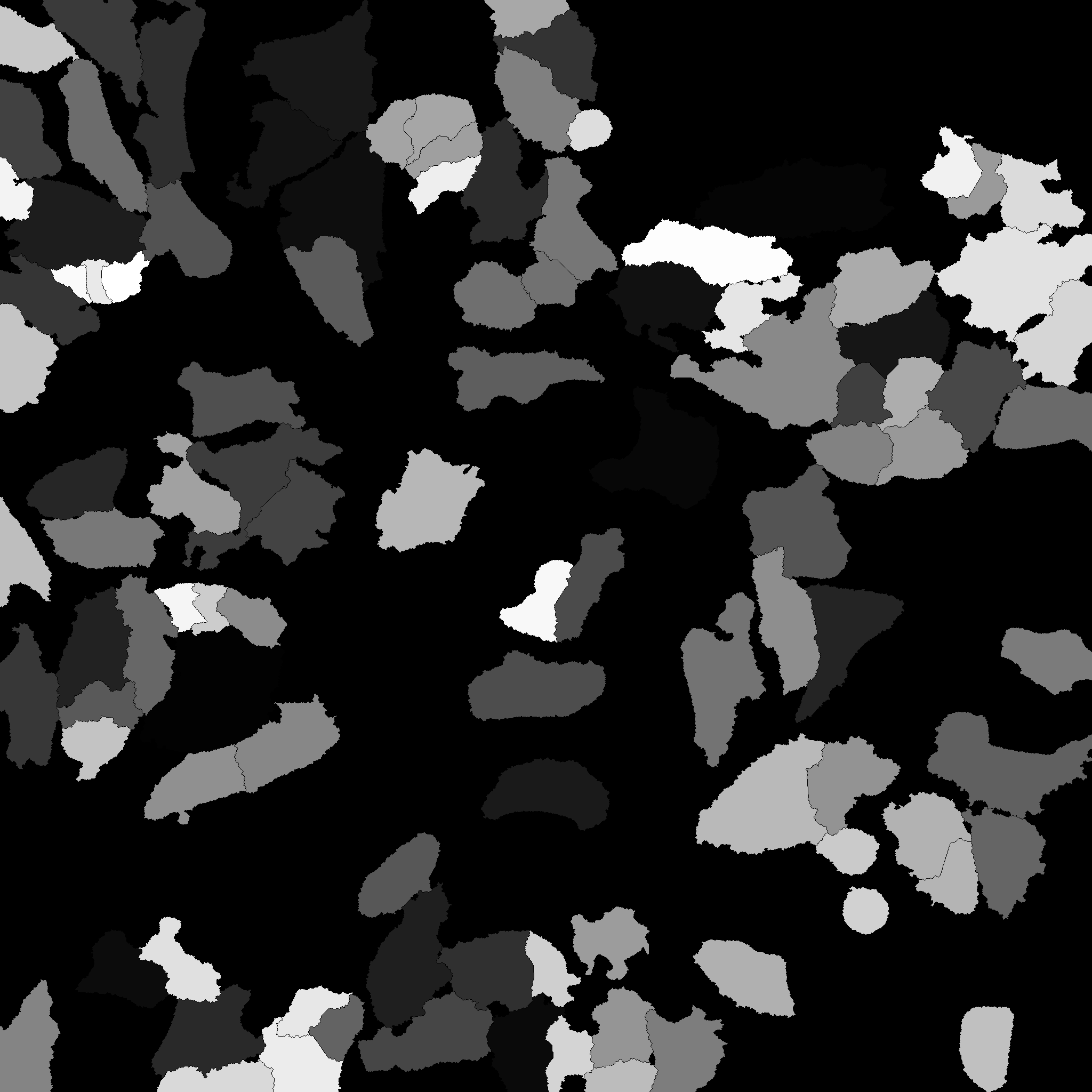

Supplement: Supplementary file 1 — Sample images and results. Sample datasets used in this paper (# 1 and #5 in table 2). The dataset includes input images of both dsRed and Cy5 channels and the corresponding cell segmentation. (ZIP 245,472 kb) [file 12859_2018_2375_MOESM1_ESM.zip › FYVE Hela 1/A - 10(fld 1 wv Green - dsRed)_cellseg_label.tif]

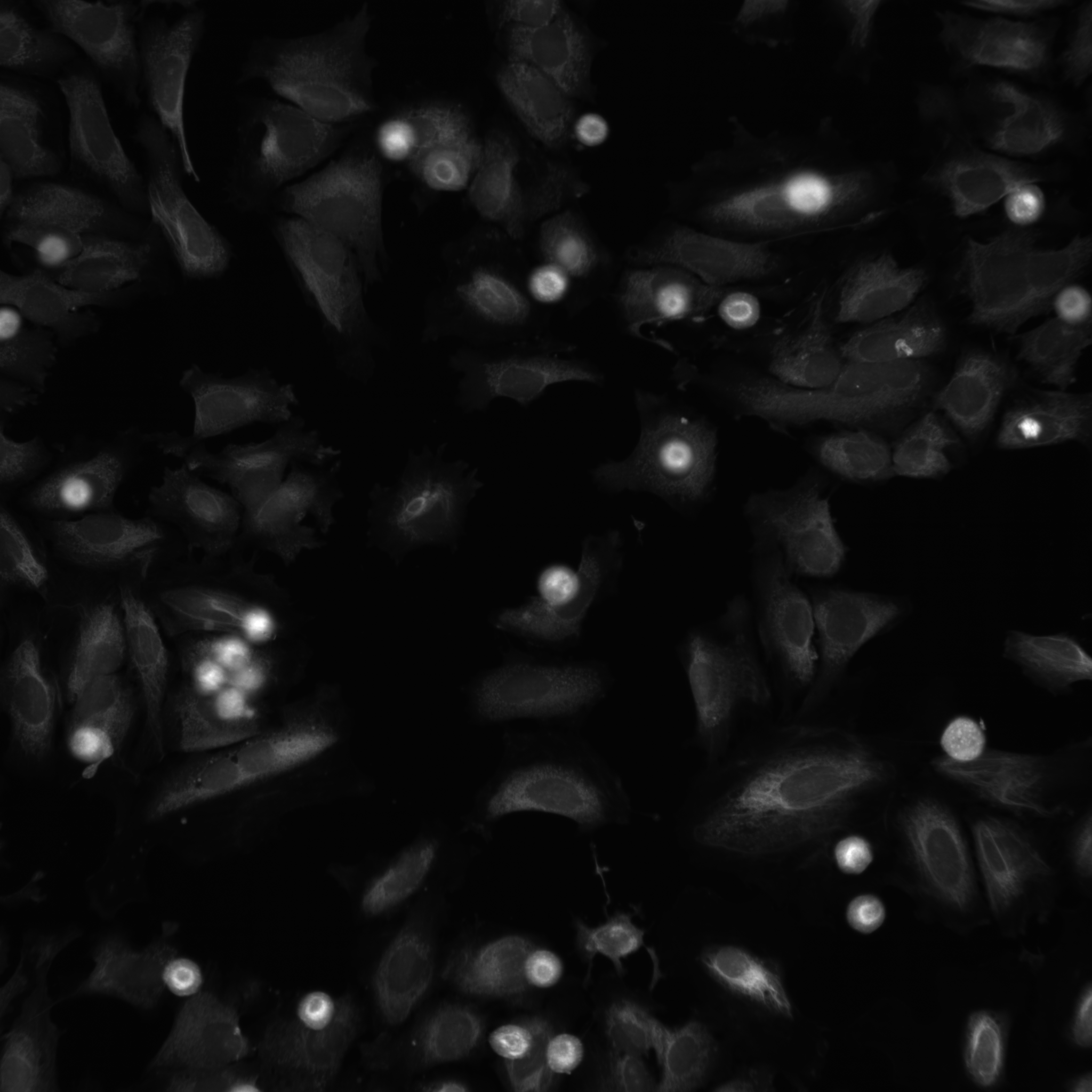

Supplement: Supplementary file 1 — Sample images and results. Sample datasets used in this paper (# 1 and #5 in table 2). The dataset includes input images of both dsRed and Cy5 channels and the corresponding cell segmentation. (ZIP 245,472 kb) [file 12859_2018_2375_MOESM1_ESM.zip › FYVE Hela 1/A - 10(fld 1 wv Red - Cy5).tif]

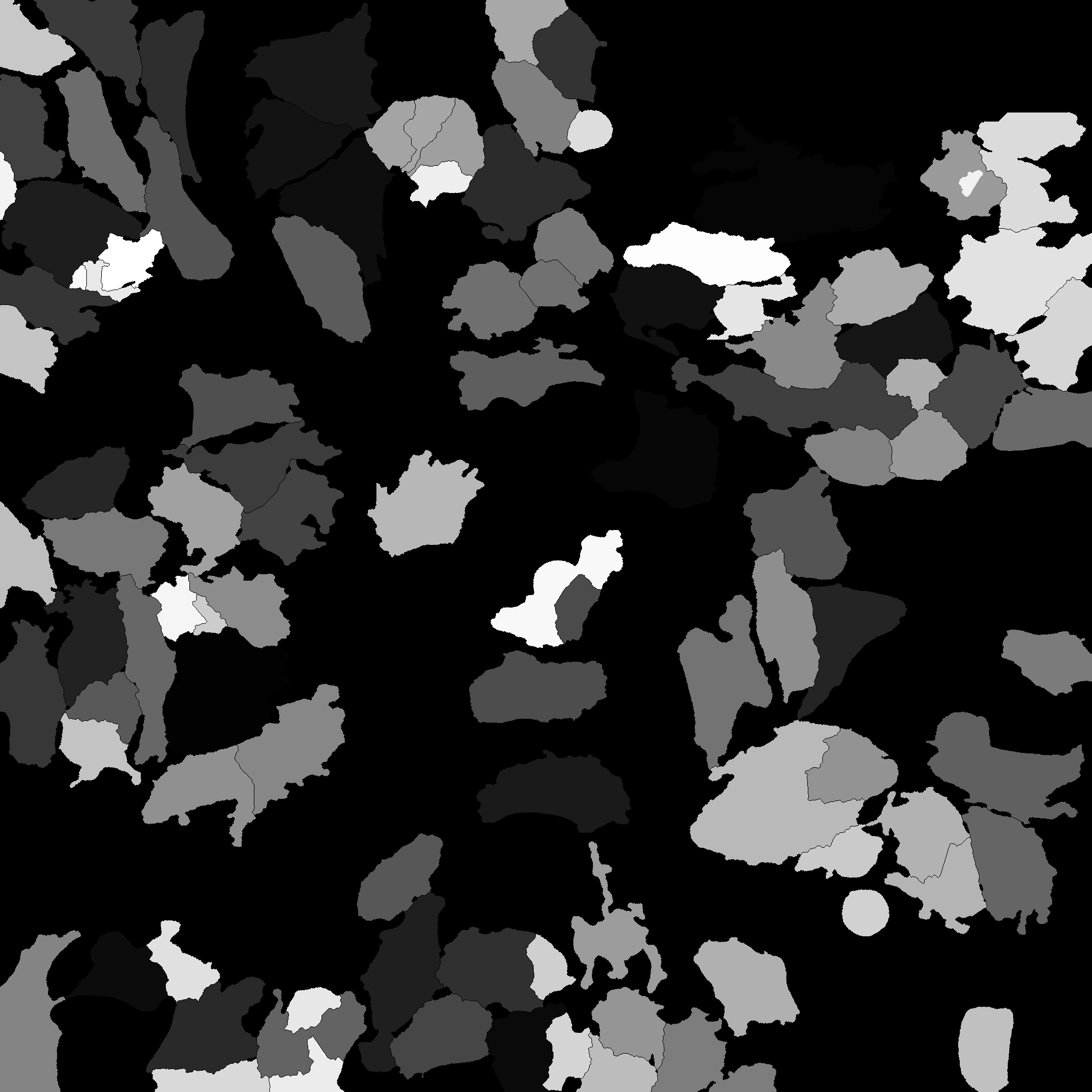

Supplement: Supplementary file 1 — Sample images and results. Sample datasets used in this paper (# 1 and #5 in table 2). The dataset includes input images of both dsRed and Cy5 channels and the corresponding cell segmentation. (ZIP 245,472 kb) [file 12859_2018_2375_MOESM1_ESM.zip › FYVE Hela 1/A - 10(fld 1 wv Red - Cy5)_cellseg_label.tif]

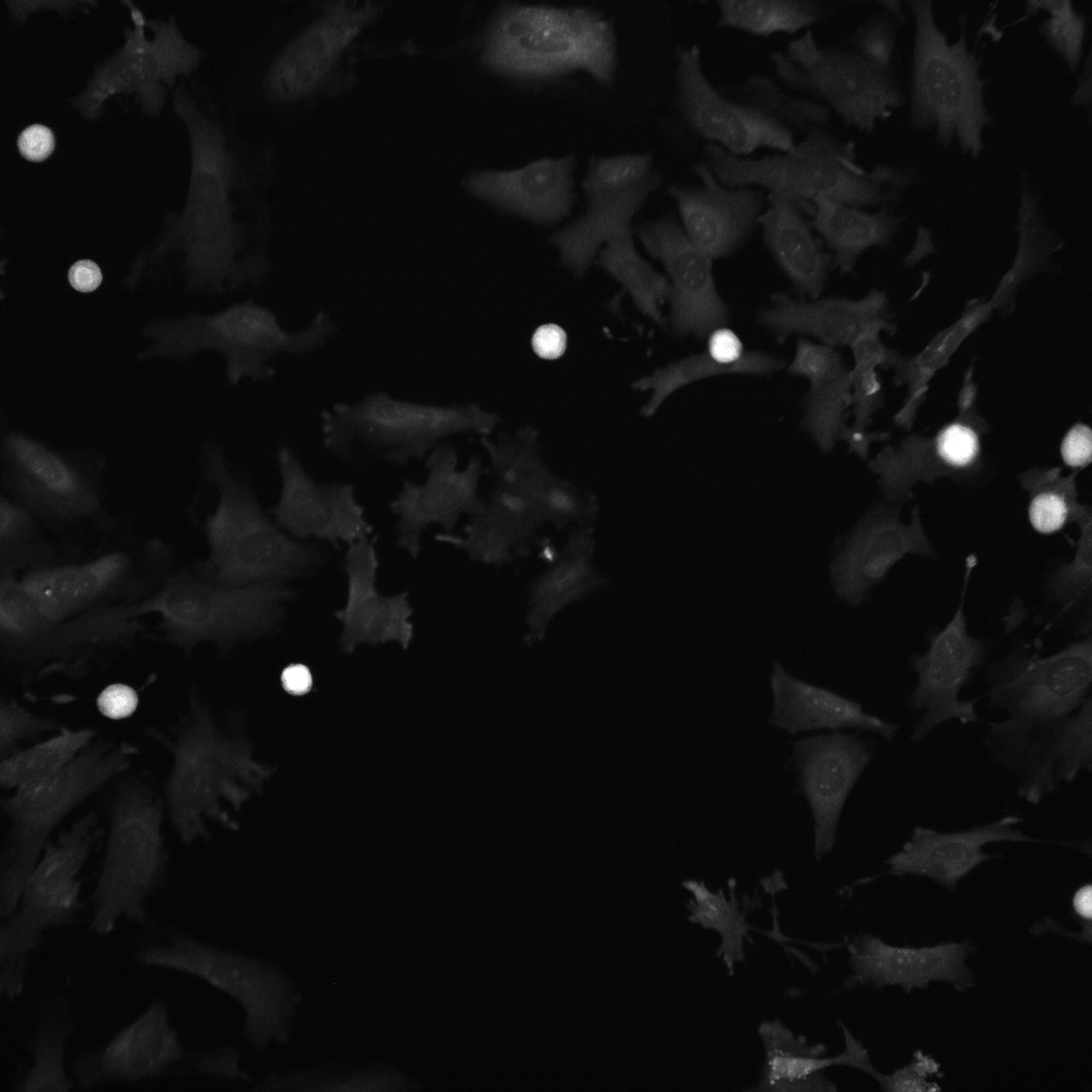

Supplement: Supplementary file 1 — Sample images and results. Sample datasets used in this paper (# 1 and #5 in table 2). The dataset includes input images of both dsRed and Cy5 channels and the corresponding cell segmentation. (ZIP 245,472 kb) [file 12859_2018_2375_MOESM1_ESM.zip › FYVE Hela 1/A - 11(fld 1 wv Green - dsRed).tif]

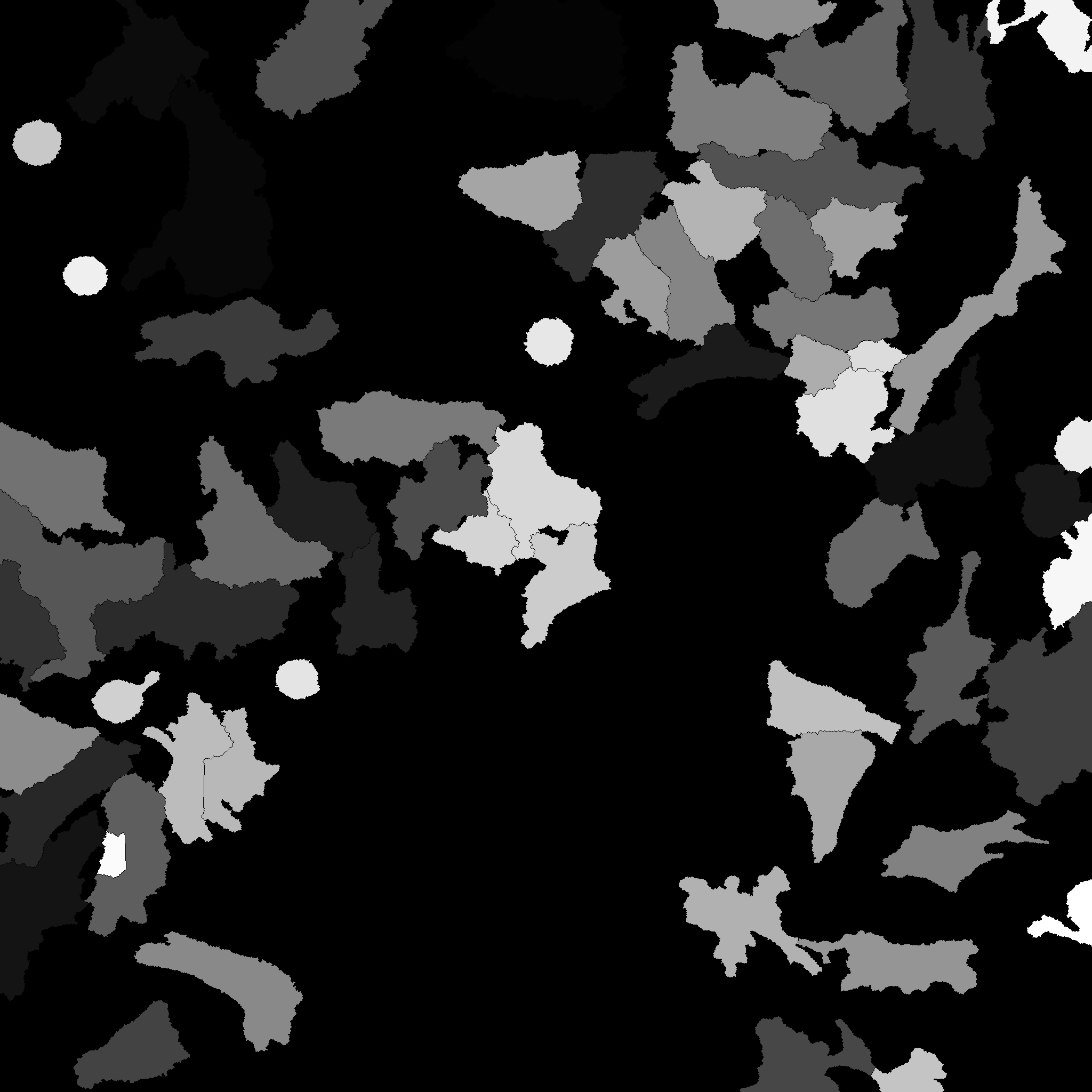

Supplement: Supplementary file 1 — Sample images and results. Sample datasets used in this paper (# 1 and #5 in table 2). The dataset includes input images of both dsRed and Cy5 channels and the corresponding cell segmentation. (ZIP 245,472 kb) [file 12859_2018_2375_MOESM1_ESM.zip › FYVE Hela 1/A - 11(fld 1 wv Green - dsRed)_cellseg_label.tif]

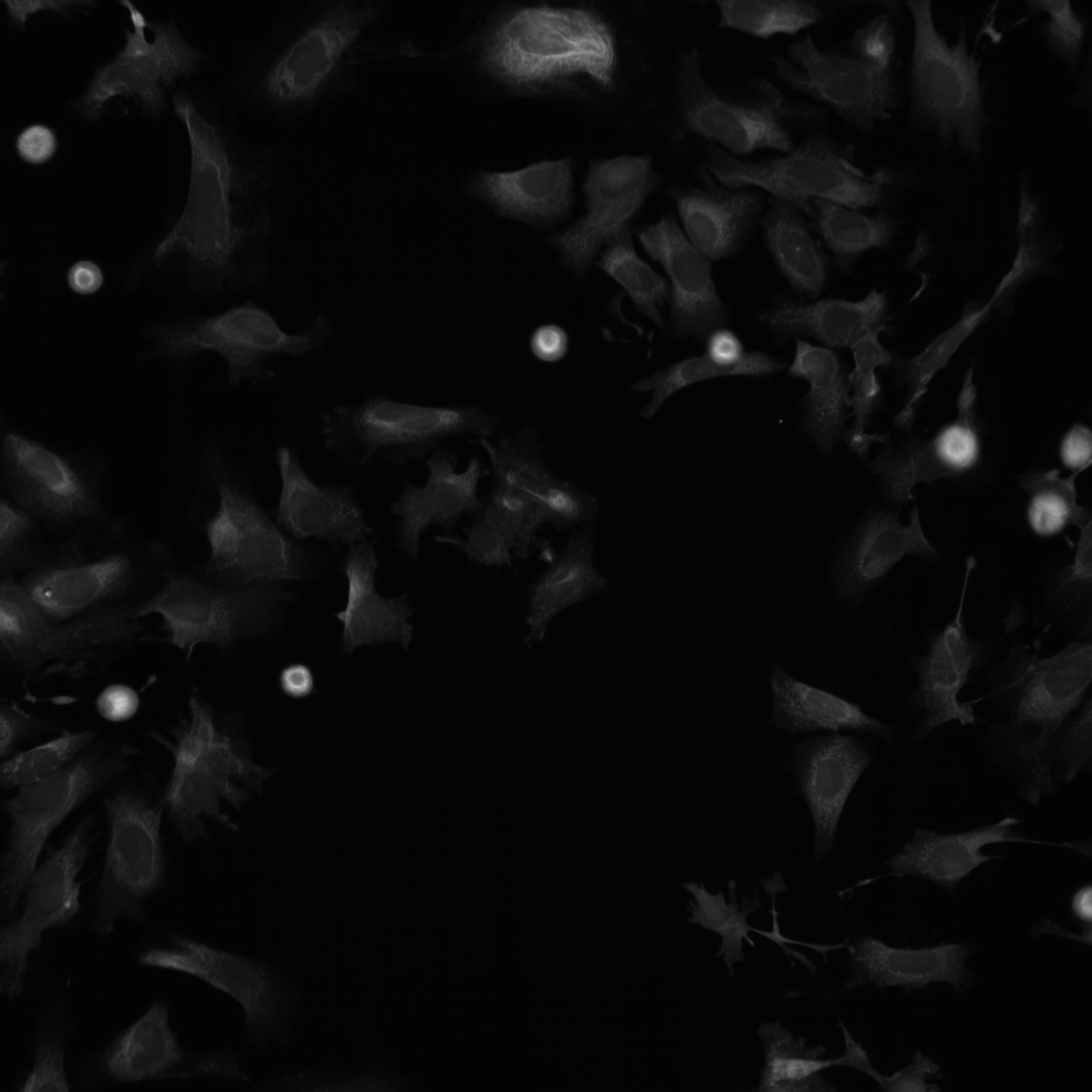

Supplement: Supplementary file 1 — Sample images and results. Sample datasets used in this paper (# 1 and #5 in table 2). The dataset includes input images of both dsRed and Cy5 channels and the corresponding cell segmentation. (ZIP 245,472 kb) [file 12859_2018_2375_MOESM1_ESM.zip › FYVE Hela 1/A - 11(fld 1 wv Red - Cy5).tif]

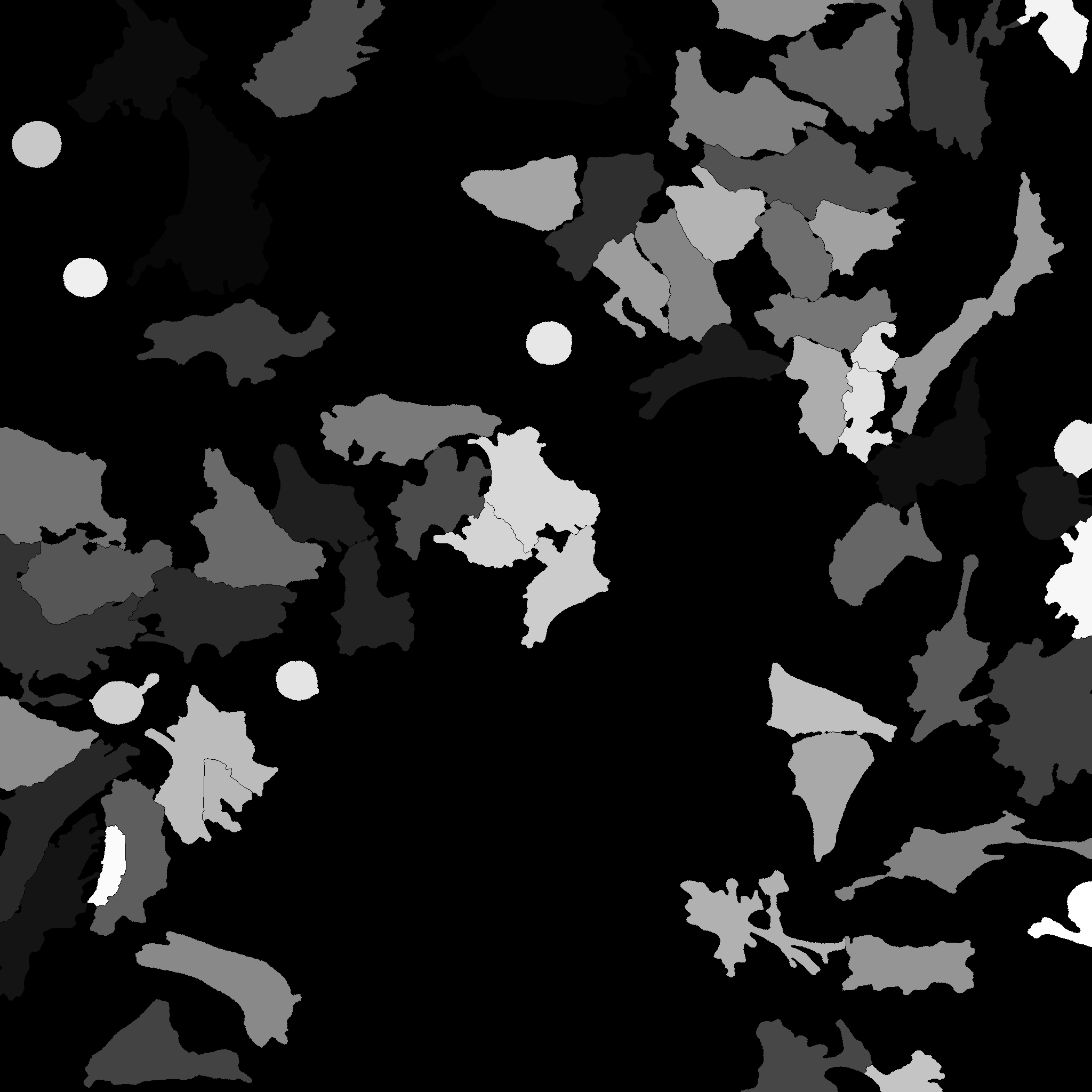

Supplement: Supplementary file 1 — Sample images and results. Sample datasets used in this paper (# 1 and #5 in table 2). The dataset includes input images of both dsRed and Cy5 channels and the corresponding cell segmentation. (ZIP 245,472 kb) [file 12859_2018_2375_MOESM1_ESM.zip › FYVE Hela 1/A - 11(fld 1 wv Red - Cy5)_cellseg_label.tif]

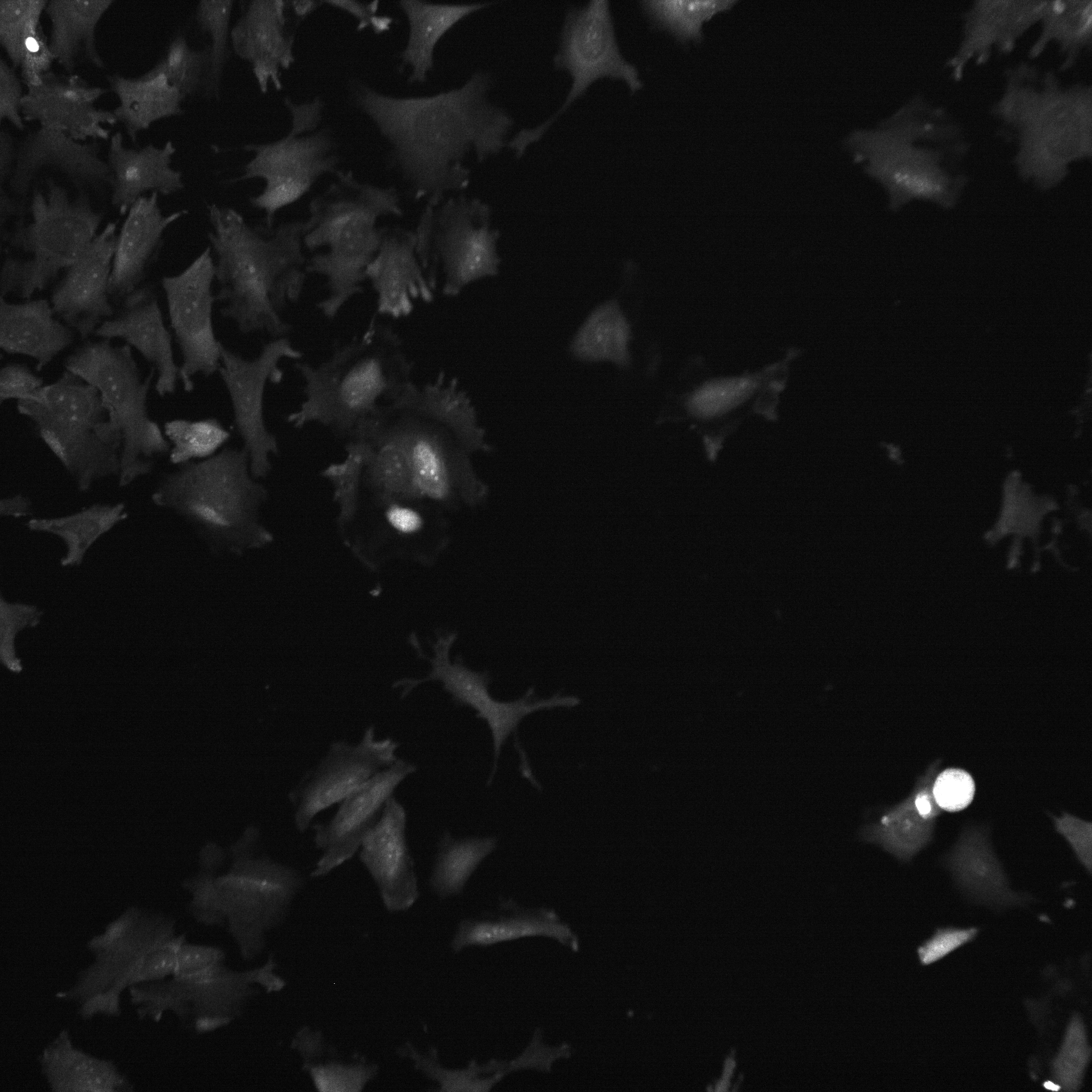

Supplement: Supplementary file 1 — Sample images and results. Sample datasets used in this paper (# 1 and #5 in table 2). The dataset includes input images of both dsRed and Cy5 channels and the corresponding cell segmentation. (ZIP 245,472 kb) [file 12859_2018_2375_MOESM1_ESM.zip › FYVE Hela 1/A - 12(fld 1 wv Green - dsRed).tif]

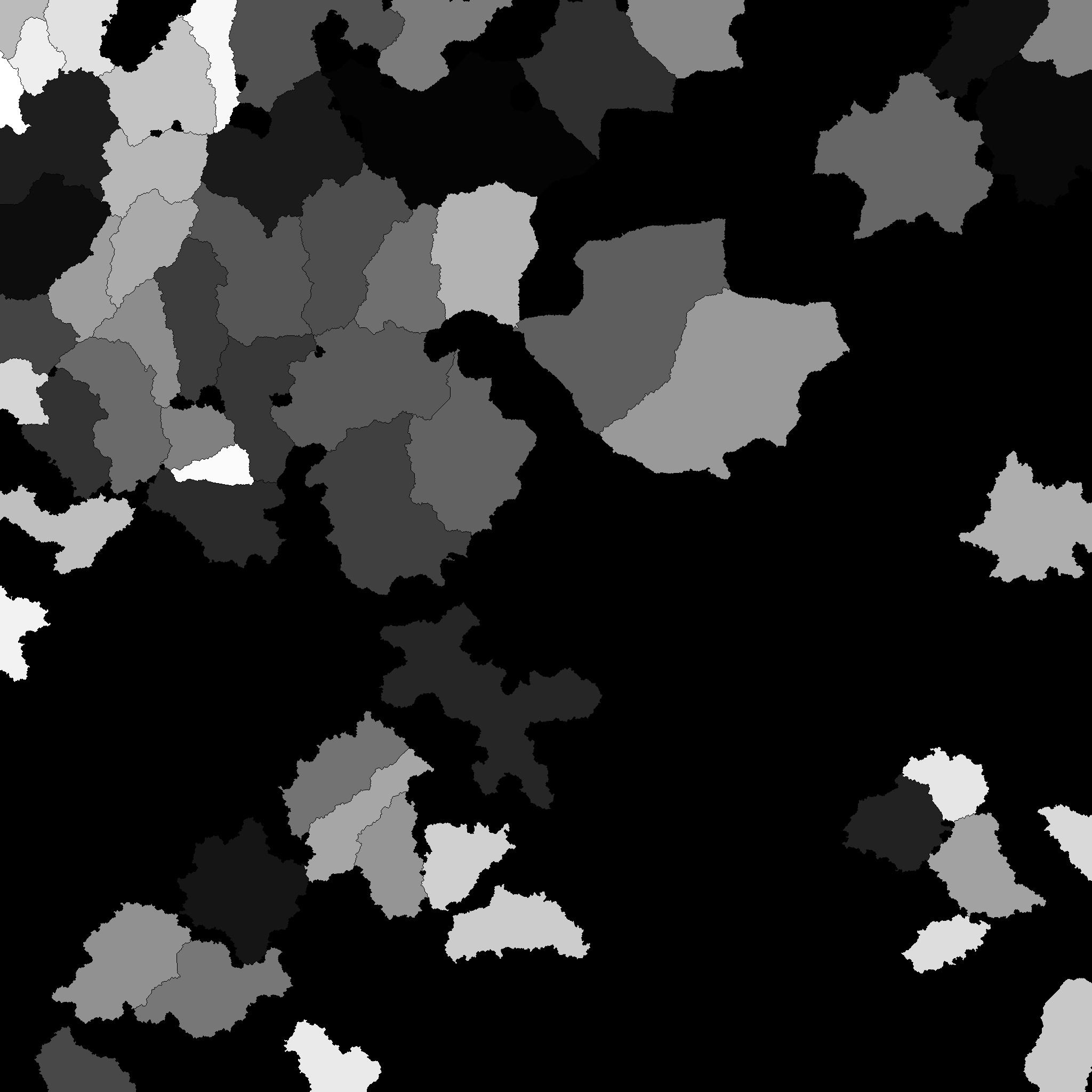

Supplement: Supplementary file 1 — Sample images and results. Sample datasets used in this paper (# 1 and #5 in table 2). The dataset includes input images of both dsRed and Cy5 channels and the corresponding cell segmentation. (ZIP 245,472 kb) [file 12859_2018_2375_MOESM1_ESM.zip › FYVE Hela 1/A - 12(fld 1 wv Green - dsRed)_cellseg_label.tif]

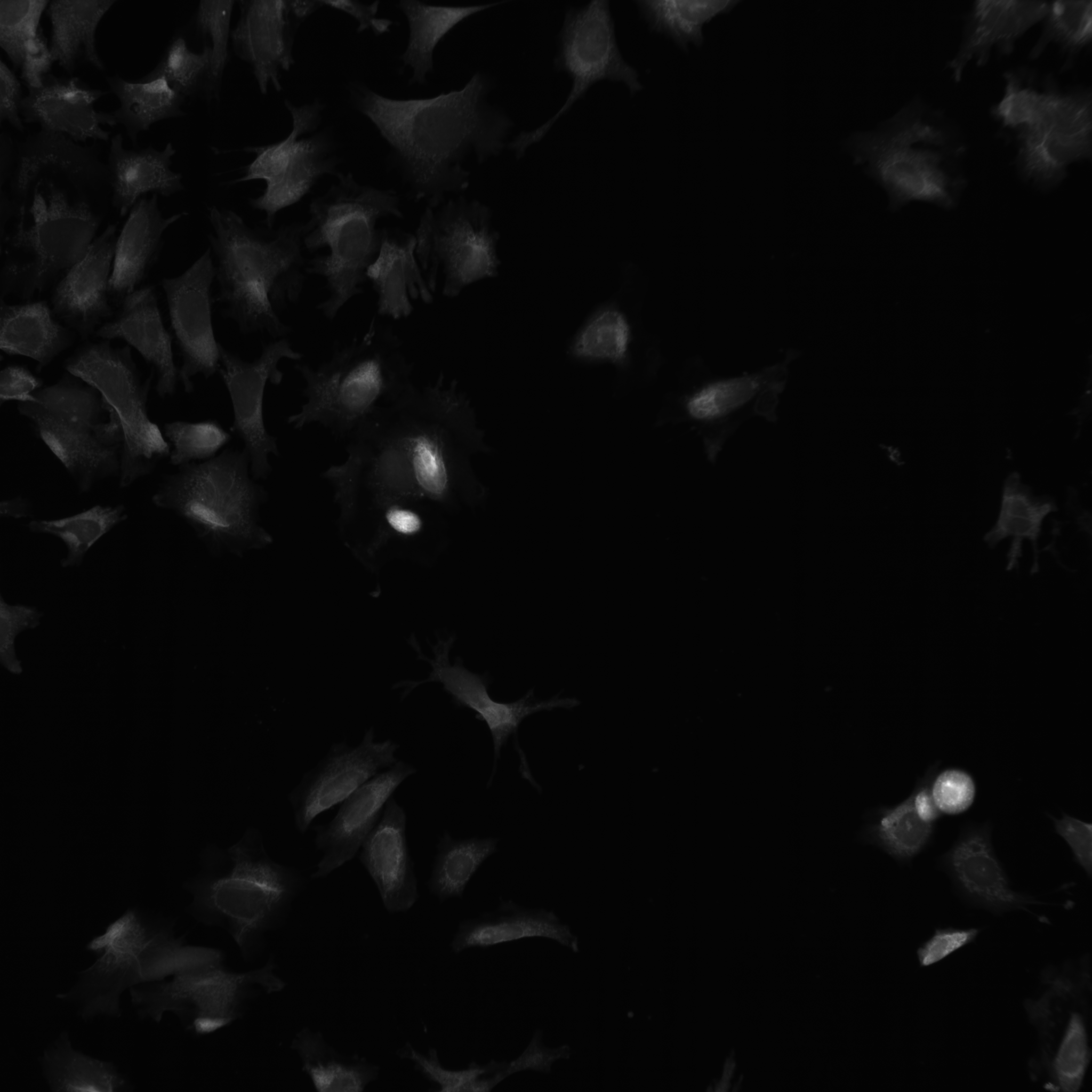

Supplement: Supplementary file 1 — Sample images and results. Sample datasets used in this paper (# 1 and #5 in table 2). The dataset includes input images of both dsRed and Cy5 channels and the corresponding cell segmentation. (ZIP 245,472 kb) [file 12859_2018_2375_MOESM1_ESM.zip › FYVE Hela 1/A - 12(fld 1 wv Red - Cy5).tif]

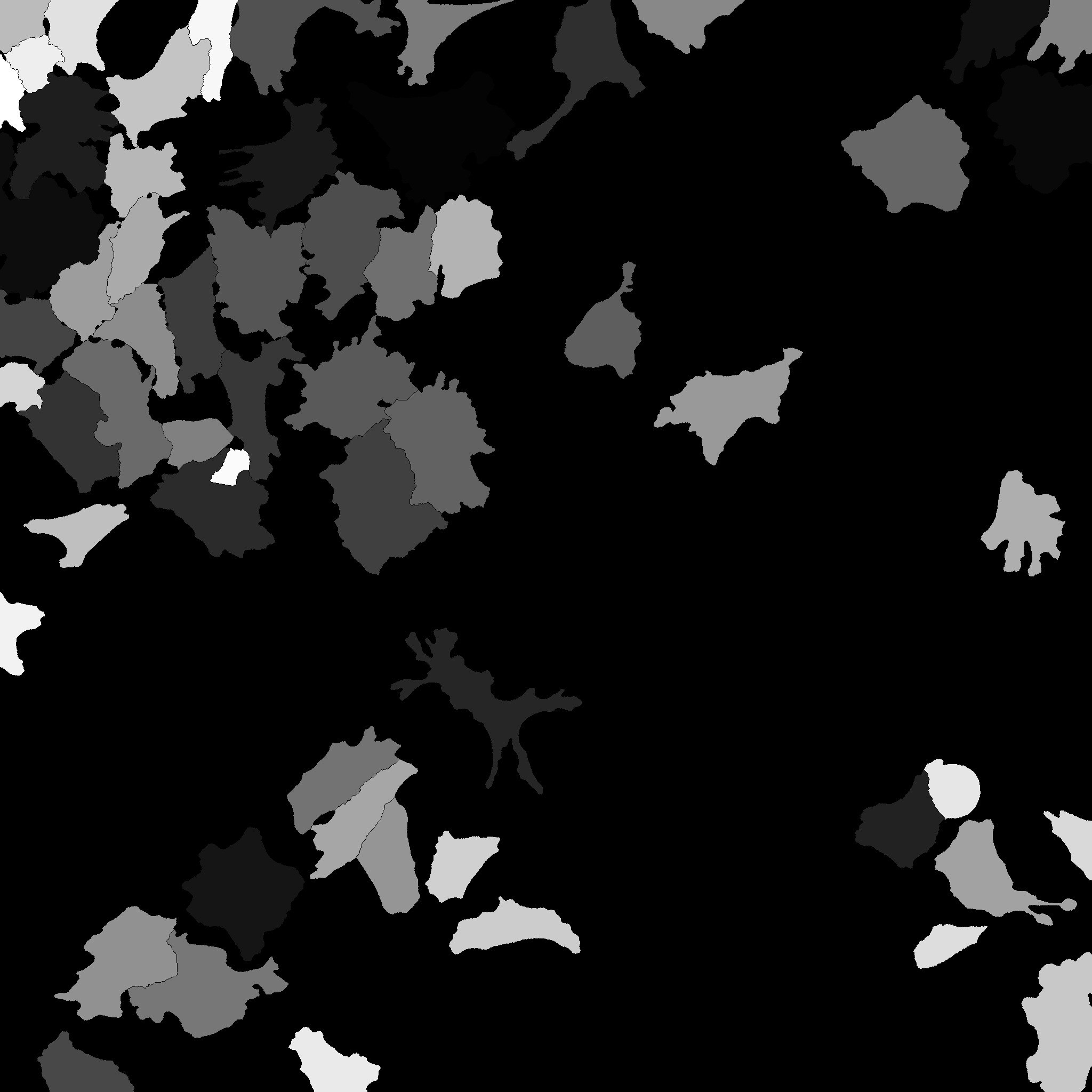

Supplement: Supplementary file 1 — Sample images and results. Sample datasets used in this paper (# 1 and #5 in table 2). The dataset includes input images of both dsRed and Cy5 channels and the corresponding cell segmentation. (ZIP 245,472 kb) [file 12859_2018_2375_MOESM1_ESM.zip › FYVE Hela 1/A - 12(fld 1 wv Red - Cy5)_cellseg_label.tif]

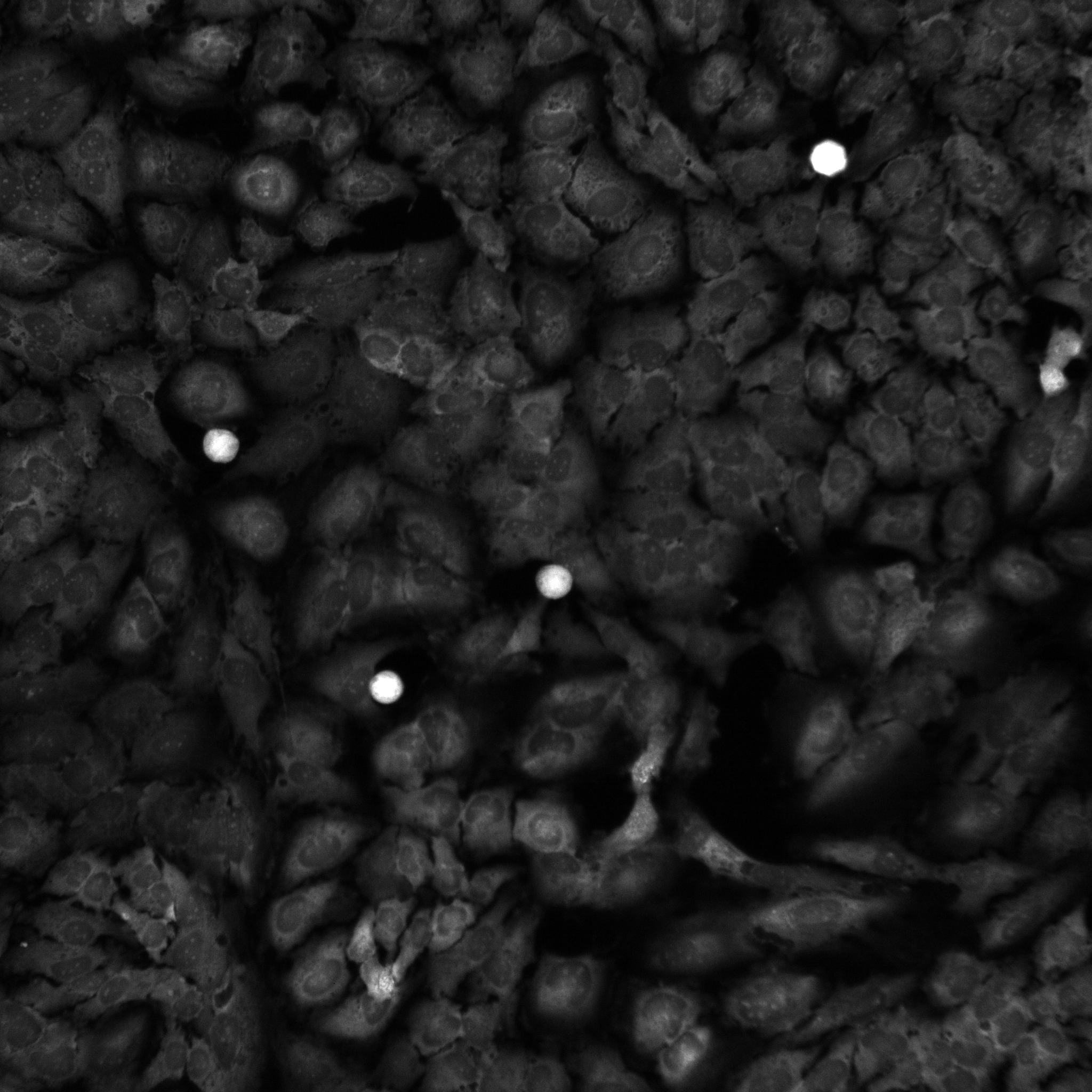

Supplement: Supplementary file 1 — Sample images and results. Sample datasets used in this paper (# 1 and #5 in table 2). The dataset includes input images of both dsRed and Cy5 channels and the corresponding cell segmentation. (ZIP 245,472 kb) [file 12859_2018_2375_MOESM1_ESM.zip › FYVE Hela 1/A - 2(fld 1 wv Green - dsRed).tif]

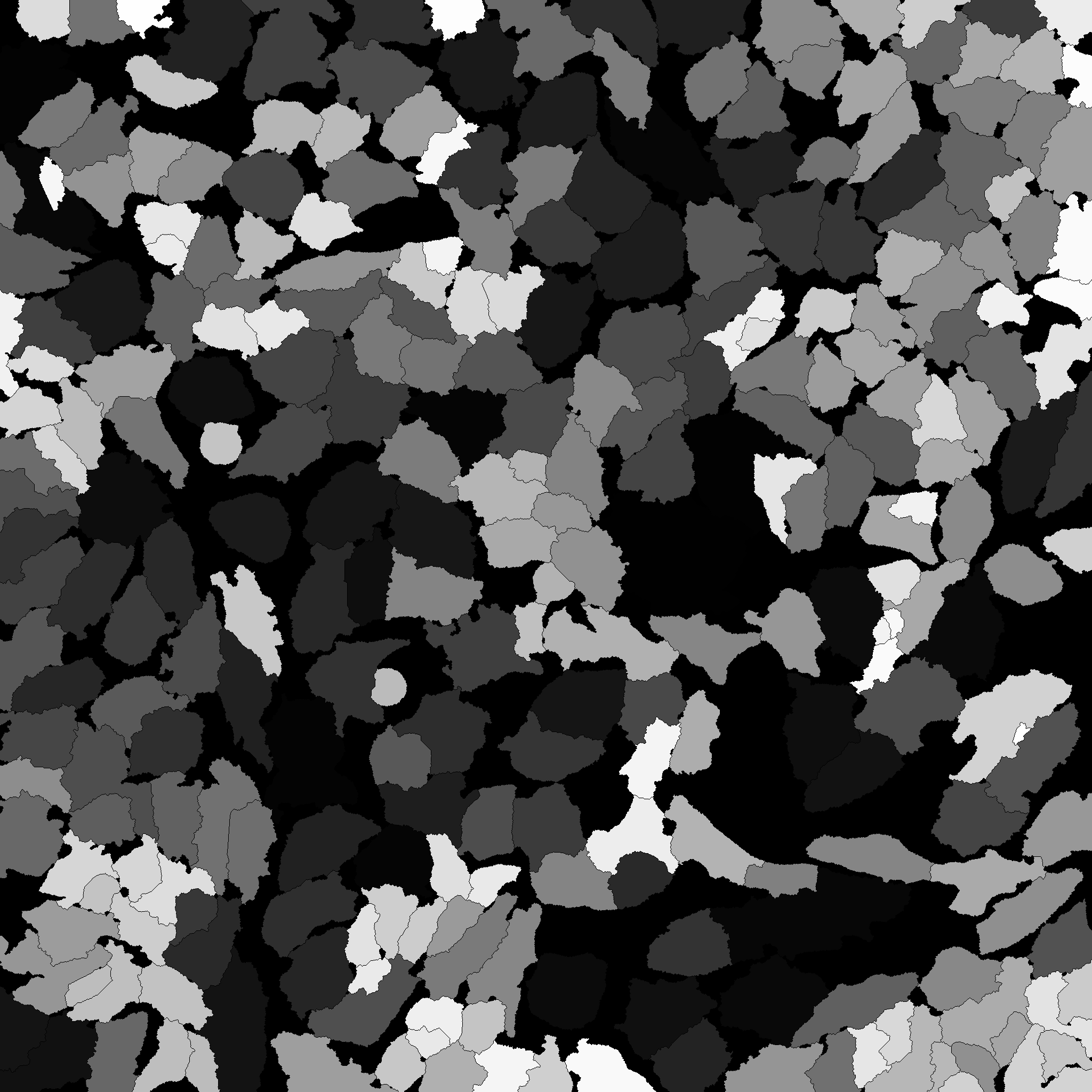

Supplement: Supplementary file 1 — Sample images and results. Sample datasets used in this paper (# 1 and #5 in table 2). The dataset includes input images of both dsRed and Cy5 channels and the corresponding cell segmentation. (ZIP 245,472 kb) [file 12859_2018_2375_MOESM1_ESM.zip › FYVE Hela 1/A - 2(fld 1 wv Green - dsRed)_cellseg_label.tif]

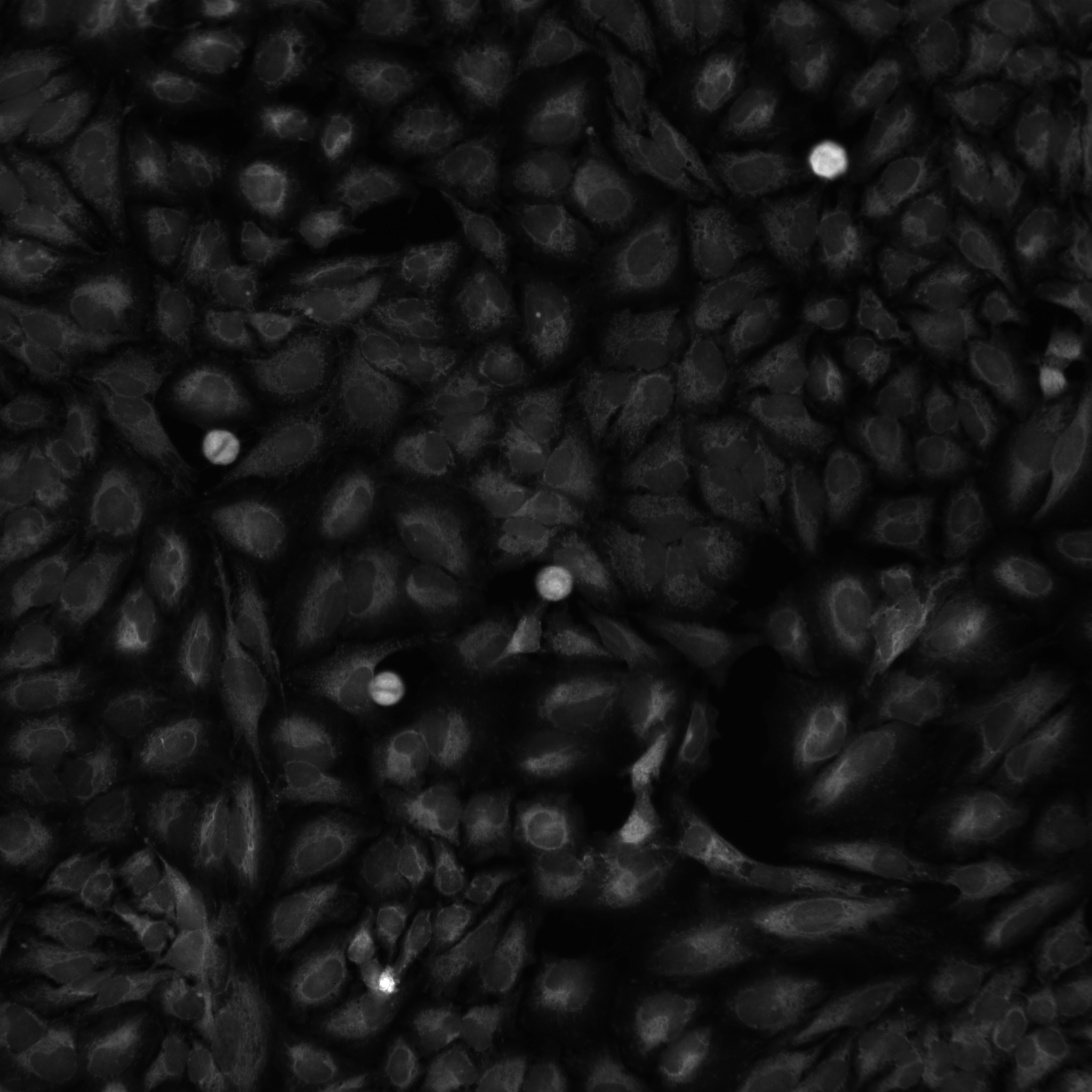

Supplement: Supplementary file 1 — Sample images and results. Sample datasets used in this paper (# 1 and #5 in table 2). The dataset includes input images of both dsRed and Cy5 channels and the corresponding cell segmentation. (ZIP 245,472 kb) [file 12859_2018_2375_MOESM1_ESM.zip › FYVE Hela 1/A - 2(fld 1 wv Red - Cy5).tif]

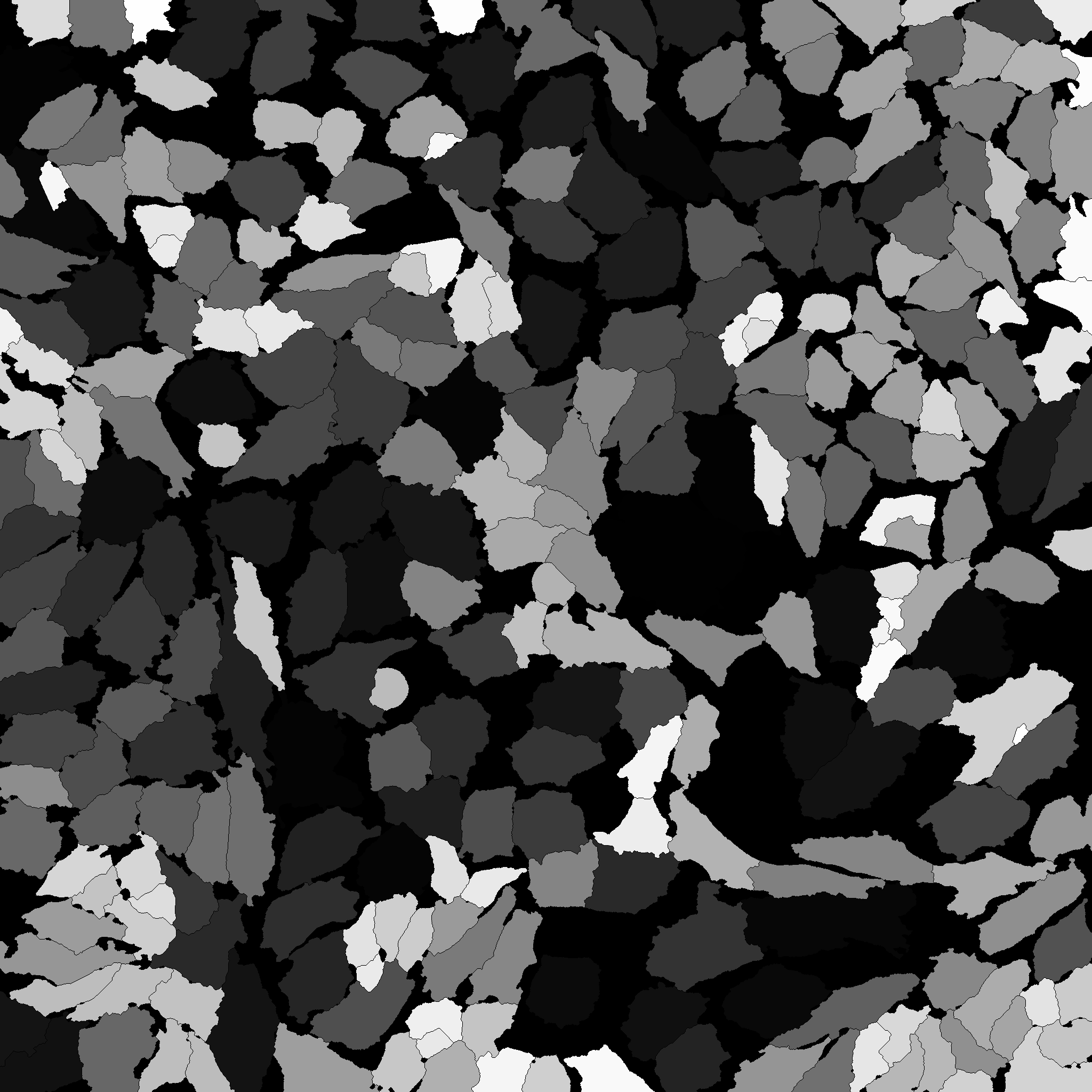

Supplement: Supplementary file 1 — Sample images and results. Sample datasets used in this paper (# 1 and #5 in table 2). The dataset includes input images of both dsRed and Cy5 channels and the corresponding cell segmentation. (ZIP 245,472 kb) [file 12859_2018_2375_MOESM1_ESM.zip › FYVE Hela 1/A - 2(fld 1 wv Red - Cy5)_cellseg_label.tif]

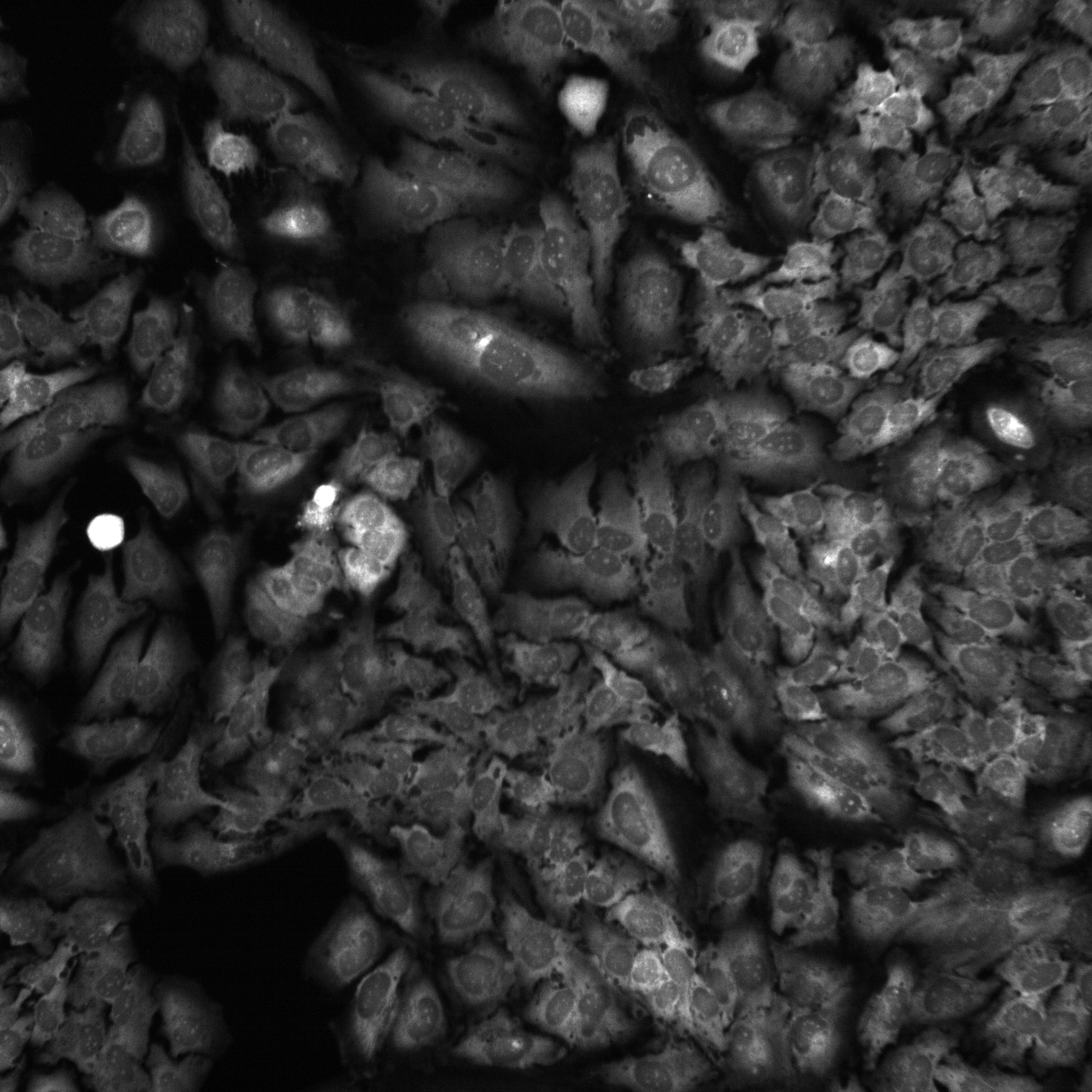

Supplement: Supplementary file 1 — Sample images and results. Sample datasets used in this paper (# 1 and #5 in table 2). The dataset includes input images of both dsRed and Cy5 channels and the corresponding cell segmentation. (ZIP 245,472 kb) [file 12859_2018_2375_MOESM1_ESM.zip › FYVE Hela 1/A - 3(fld 1 wv Green - dsRed).tif]

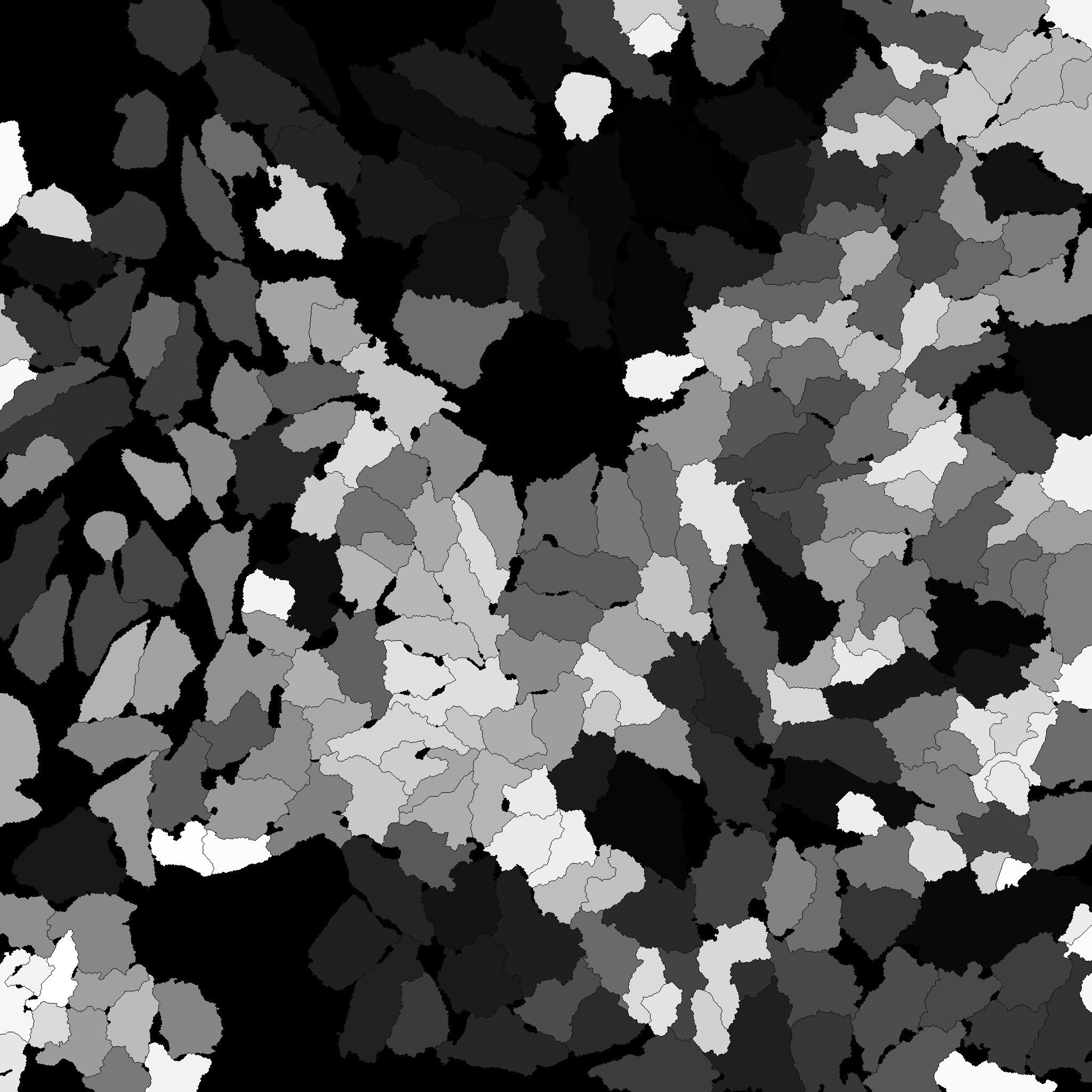

Supplement: Supplementary file 1 — Sample images and results. Sample datasets used in this paper (# 1 and #5 in table 2). The dataset includes input images of both dsRed and Cy5 channels and the corresponding cell segmentation. (ZIP 245,472 kb) [file 12859_2018_2375_MOESM1_ESM.zip › FYVE Hela 1/A - 3(fld 1 wv Green - dsRed)_cellseg_label.tif]

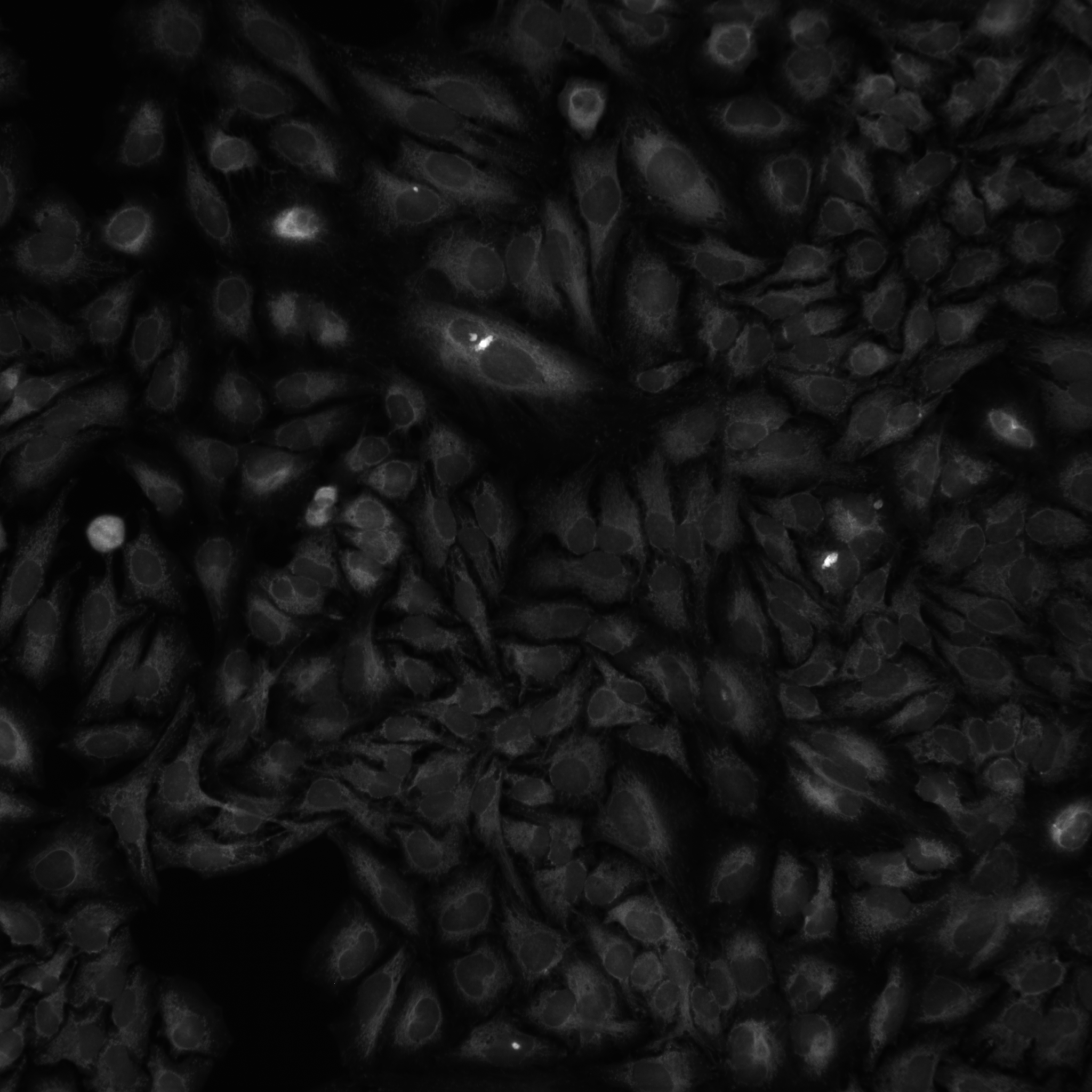

Supplement: Supplementary file 1 — Sample images and results. Sample datasets used in this paper (# 1 and #5 in table 2). The dataset includes input images of both dsRed and Cy5 channels and the corresponding cell segmentation. (ZIP 245,472 kb) [file 12859_2018_2375_MOESM1_ESM.zip › FYVE Hela 1/A - 3(fld 1 wv Red - Cy5).tif]

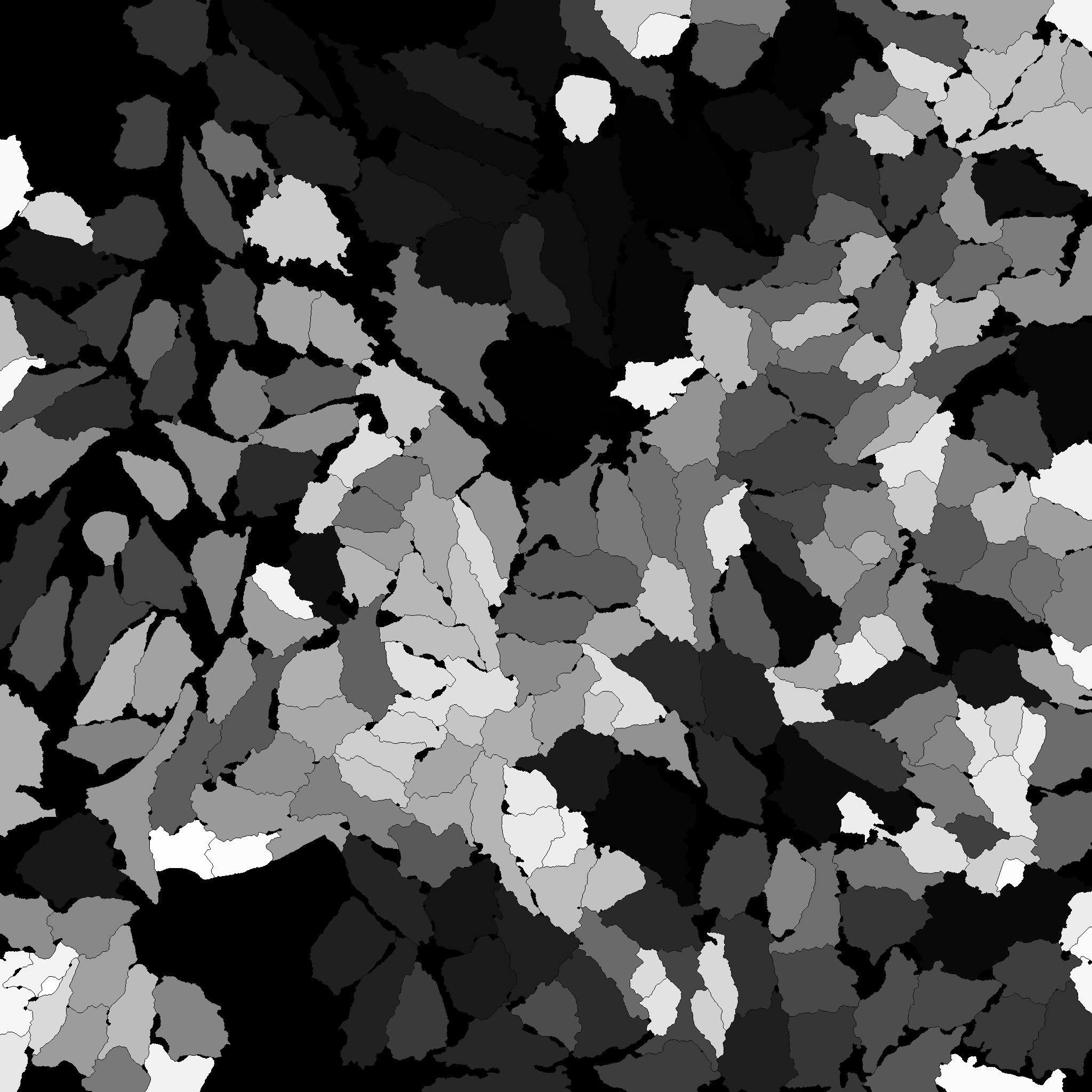

Supplement: Supplementary file 1 — Sample images and results. Sample datasets used in this paper (# 1 and #5 in table 2). The dataset includes input images of both dsRed and Cy5 channels and the corresponding cell segmentation. (ZIP 245,472 kb) [file 12859_2018_2375_MOESM1_ESM.zip › FYVE Hela 1/A - 3(fld 1 wv Red - Cy5)_cellseg_label.tif]

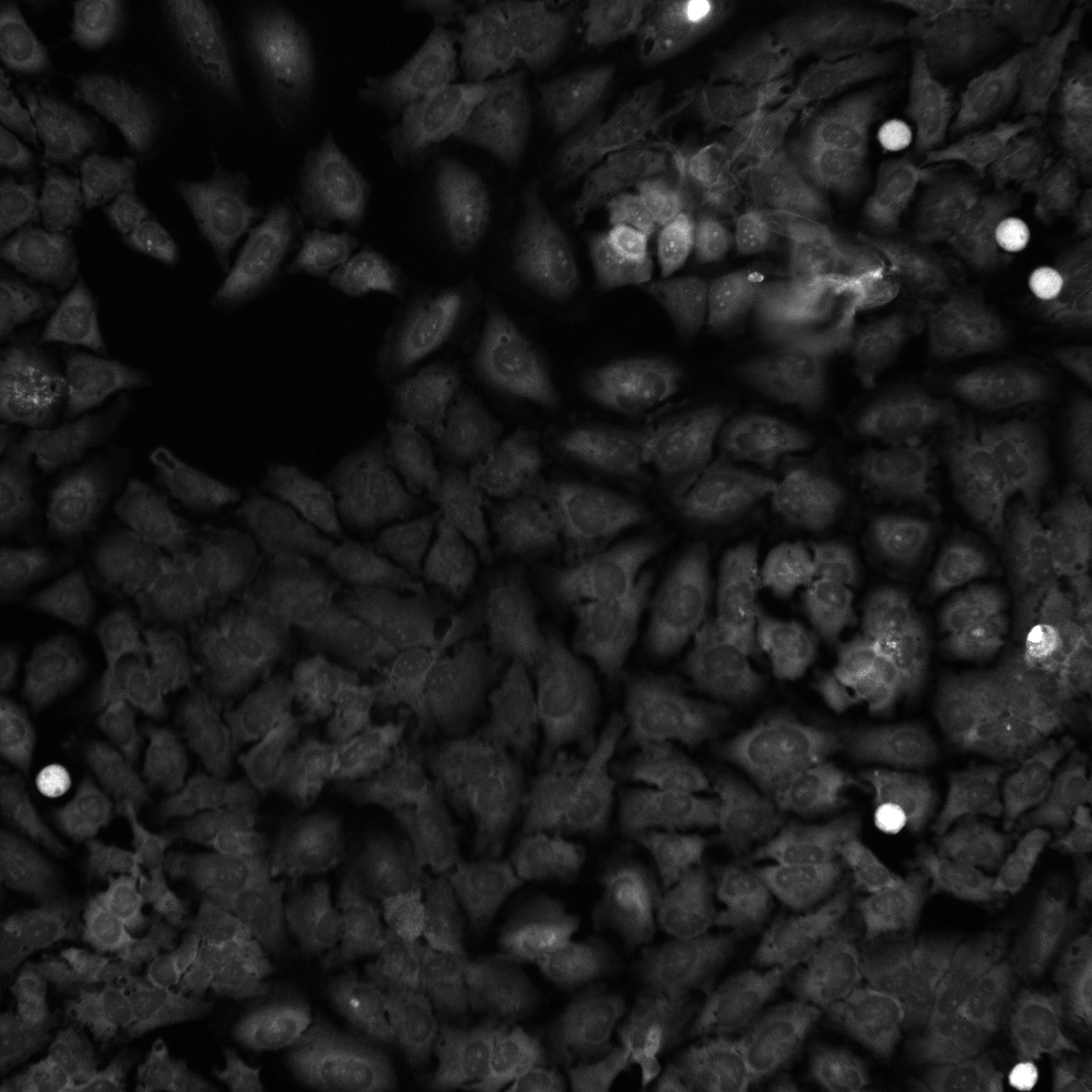

Supplement: Supplementary file 1 — Sample images and results. Sample datasets used in this paper (# 1 and #5 in table 2). The dataset includes input images of both dsRed and Cy5 channels and the corresponding cell segmentation. (ZIP 245,472 kb) [file 12859_2018_2375_MOESM1_ESM.zip › FYVE Hela 1/A - 4(fld 1 wv Green - dsRed).tif]

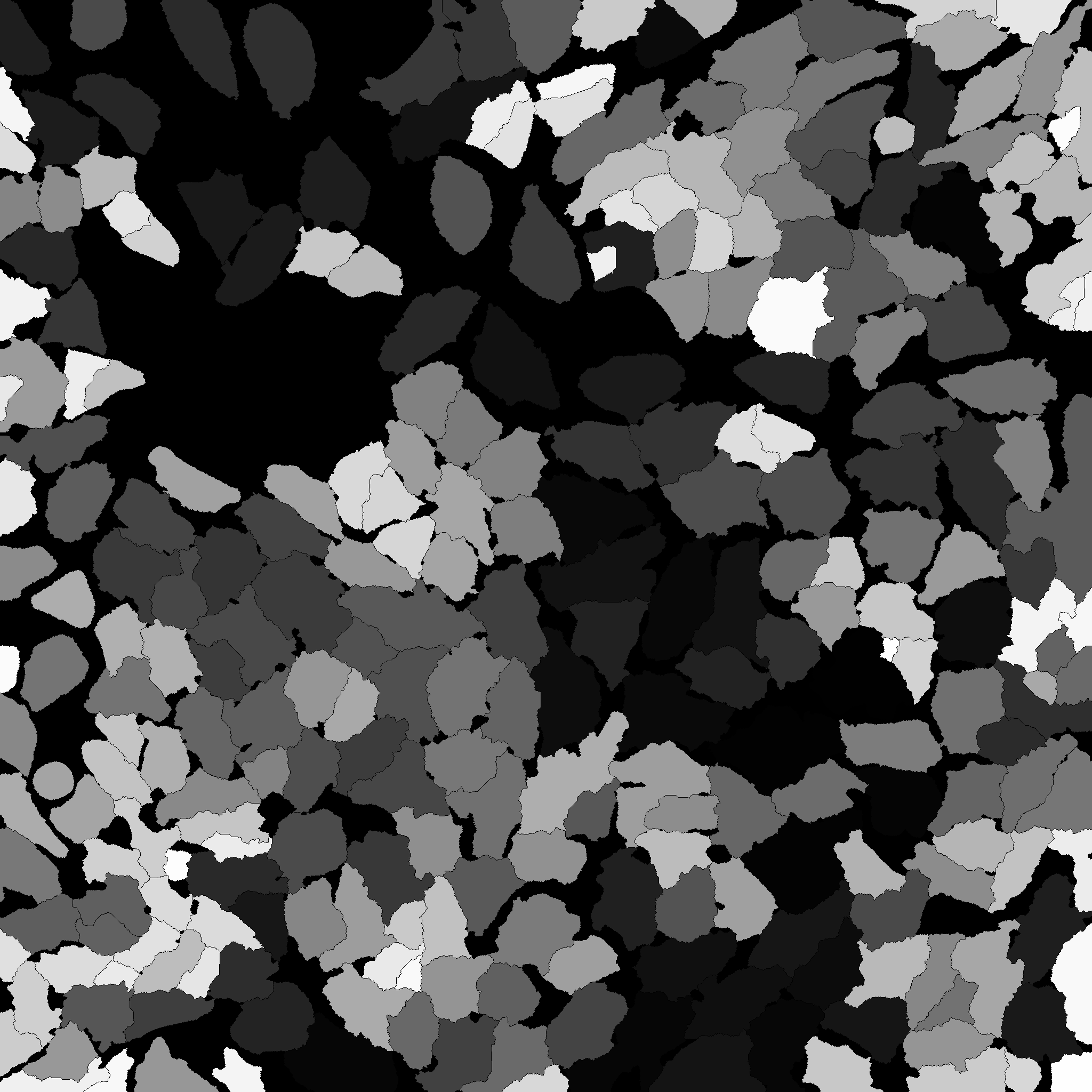

Supplement: Supplementary file 1 — Sample images and results. Sample datasets used in this paper (# 1 and #5 in table 2). The dataset includes input images of both dsRed and Cy5 channels and the corresponding cell segmentation. (ZIP 245,472 kb) [file 12859_2018_2375_MOESM1_ESM.zip › FYVE Hela 1/A - 4(fld 1 wv Green - dsRed)_cellseg_label.tif]

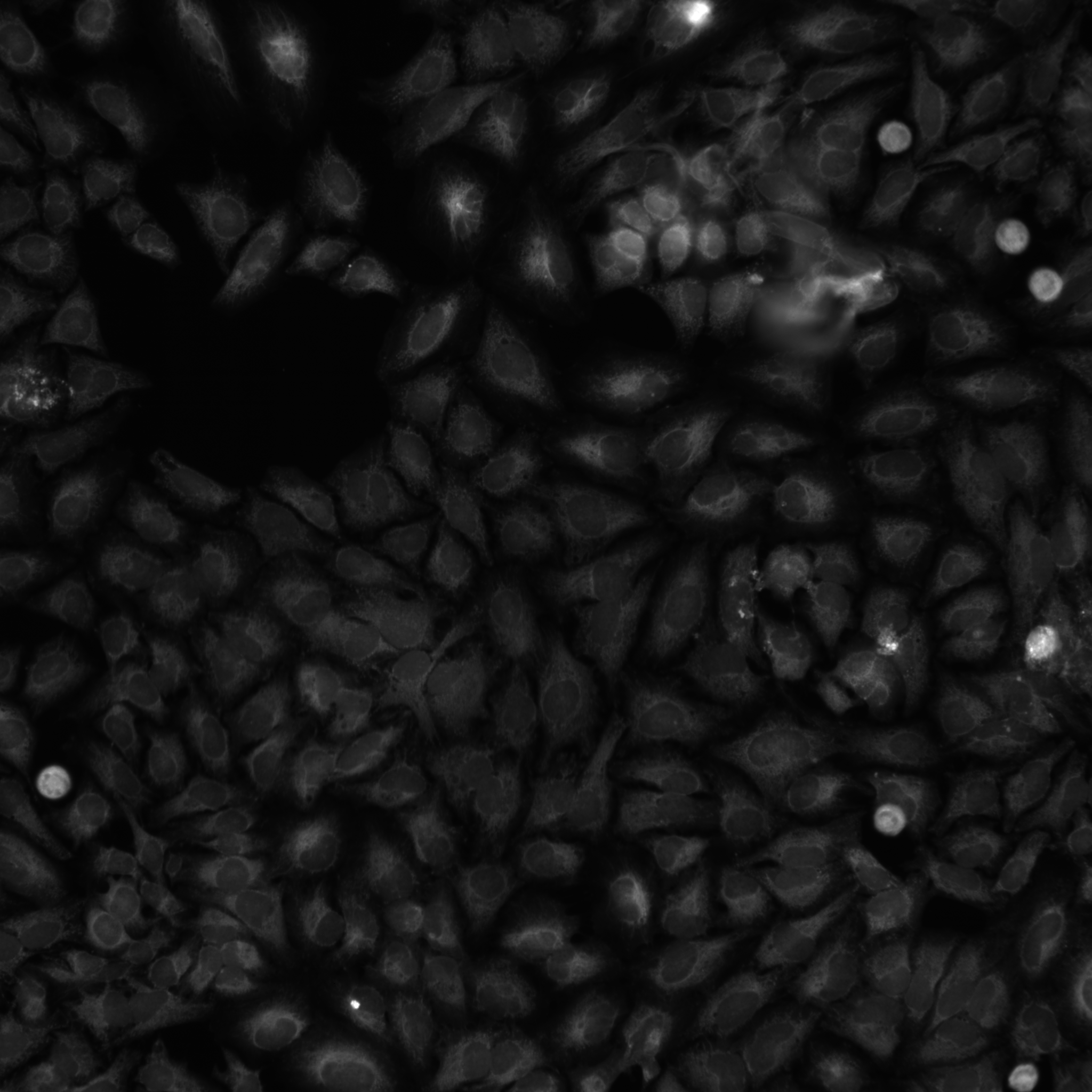

Supplement: Supplementary file 1 — Sample images and results. Sample datasets used in this paper (# 1 and #5 in table 2). The dataset includes input images of both dsRed and Cy5 channels and the corresponding cell segmentation. (ZIP 245,472 kb) [file 12859_2018_2375_MOESM1_ESM.zip › FYVE Hela 1/A - 4(fld 1 wv Red - Cy5).tif]

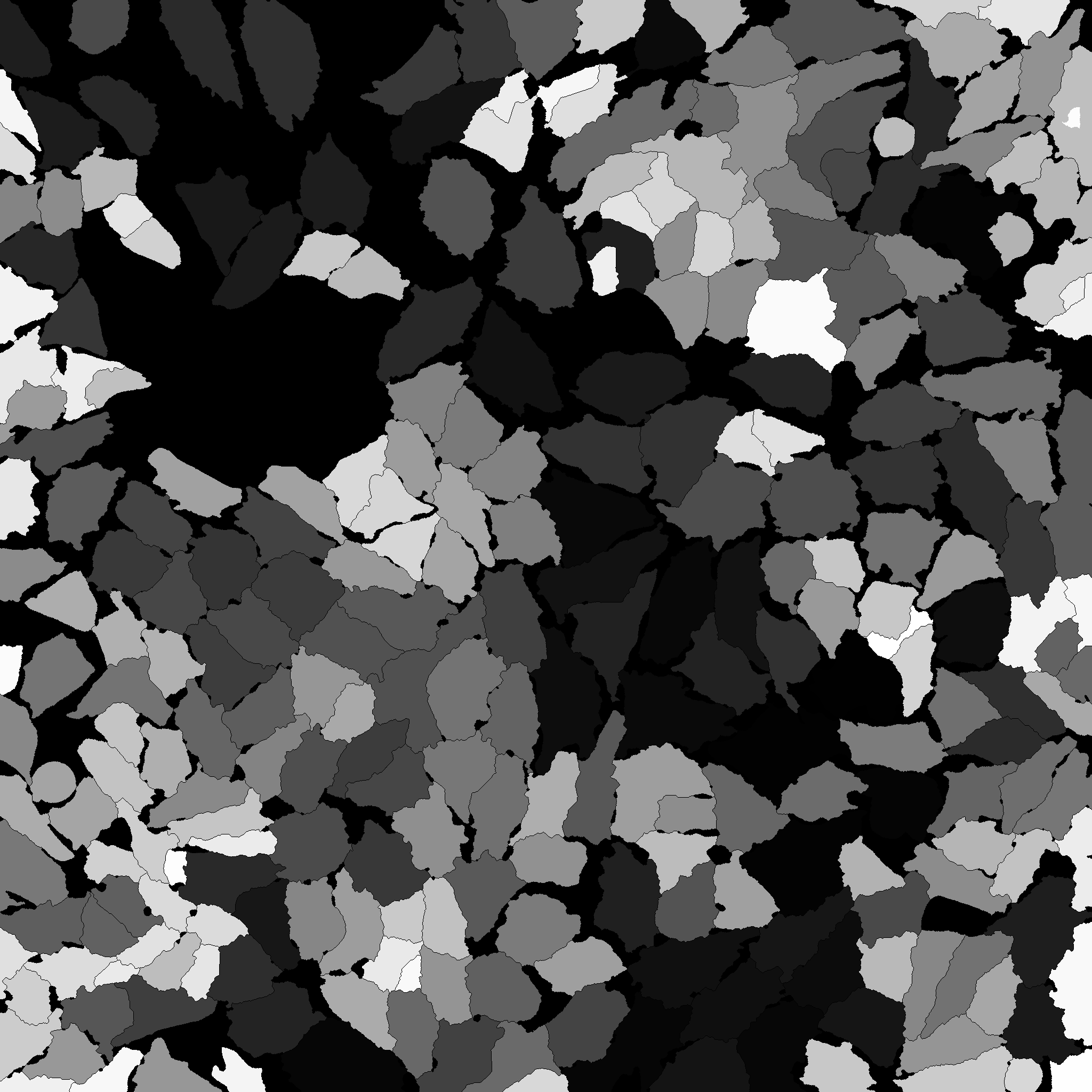

Supplement: Supplementary file 1 — Sample images and results. Sample datasets used in this paper (# 1 and #5 in table 2). The dataset includes input images of both dsRed and Cy5 channels and the corresponding cell segmentation. (ZIP 245,472 kb) [file 12859_2018_2375_MOESM1_ESM.zip › FYVE Hela 1/A - 4(fld 1 wv Red - Cy5)_cellseg_label.tif]

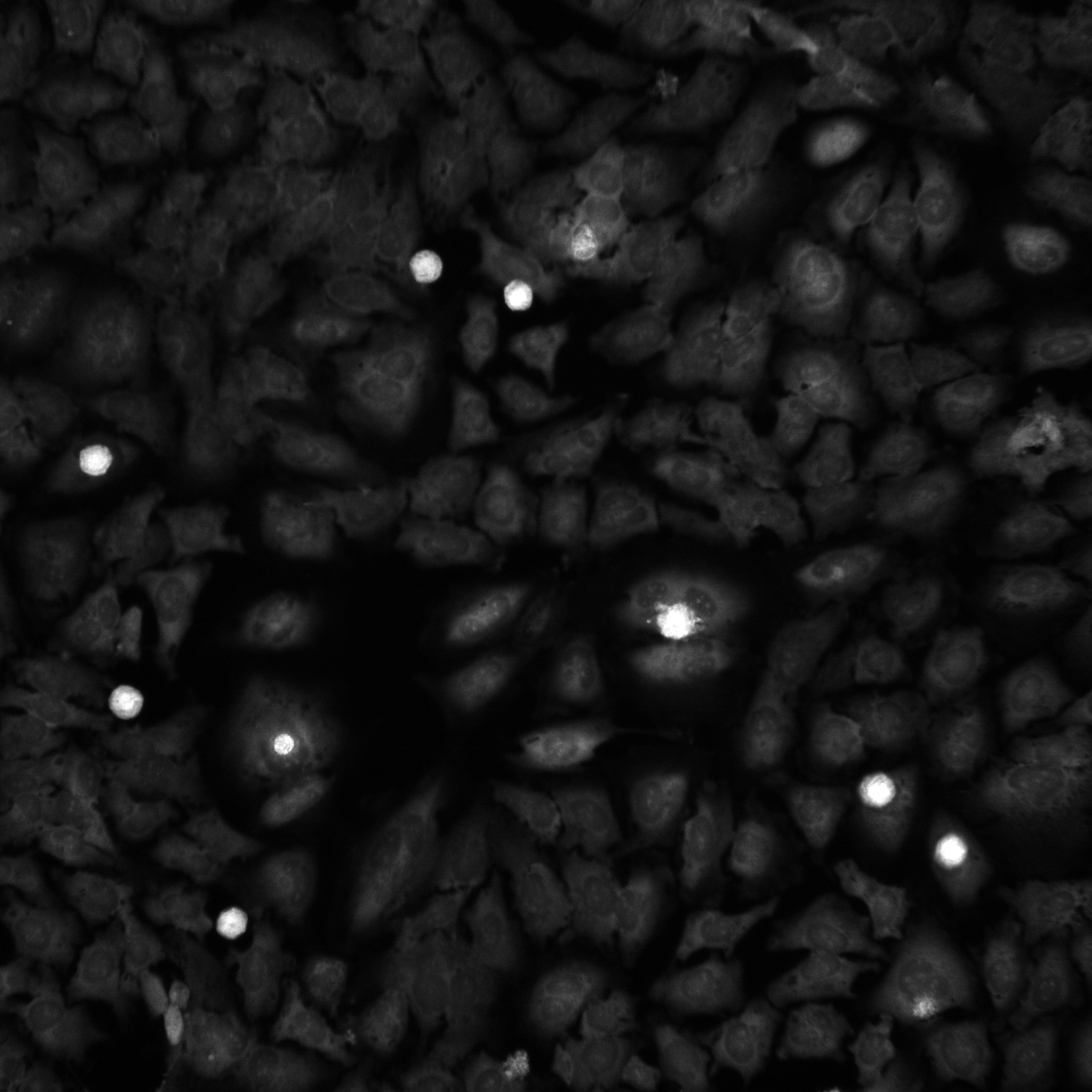

Supplement: Supplementary file 1 — Sample images and results. Sample datasets used in this paper (# 1 and #5 in table 2). The dataset includes input images of both dsRed and Cy5 channels and the corresponding cell segmentation. (ZIP 245,472 kb) [file 12859_2018_2375_MOESM1_ESM.zip › FYVE Hela 1/A - 5(fld 1 wv Green - dsRed).tif]

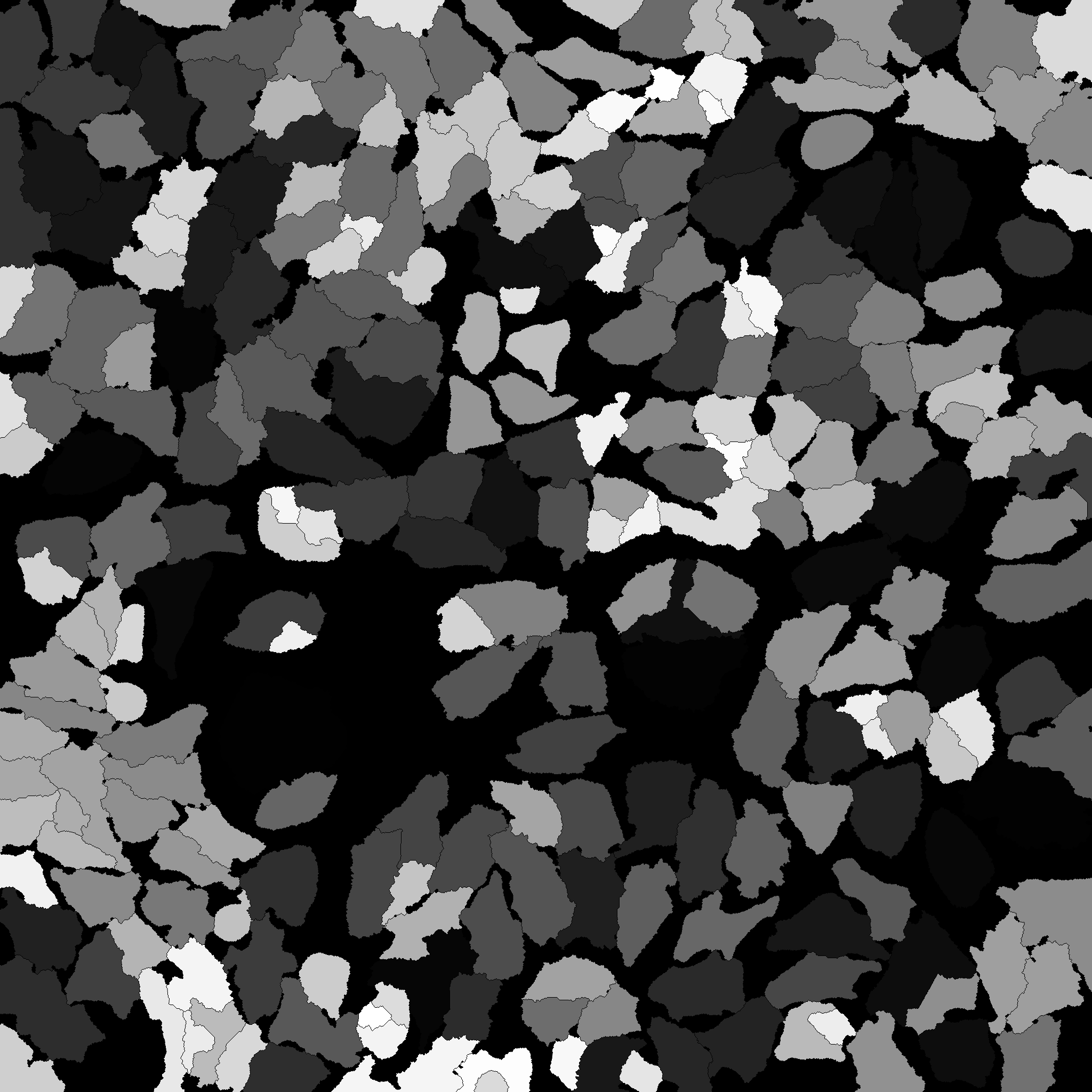

Supplement: Supplementary file 1 — Sample images and results. Sample datasets used in this paper (# 1 and #5 in table 2). The dataset includes input images of both dsRed and Cy5 channels and the corresponding cell segmentation. (ZIP 245,472 kb) [file 12859_2018_2375_MOESM1_ESM.zip › FYVE Hela 1/A - 5(fld 1 wv Green - dsRed)_cellseg_label.tif]

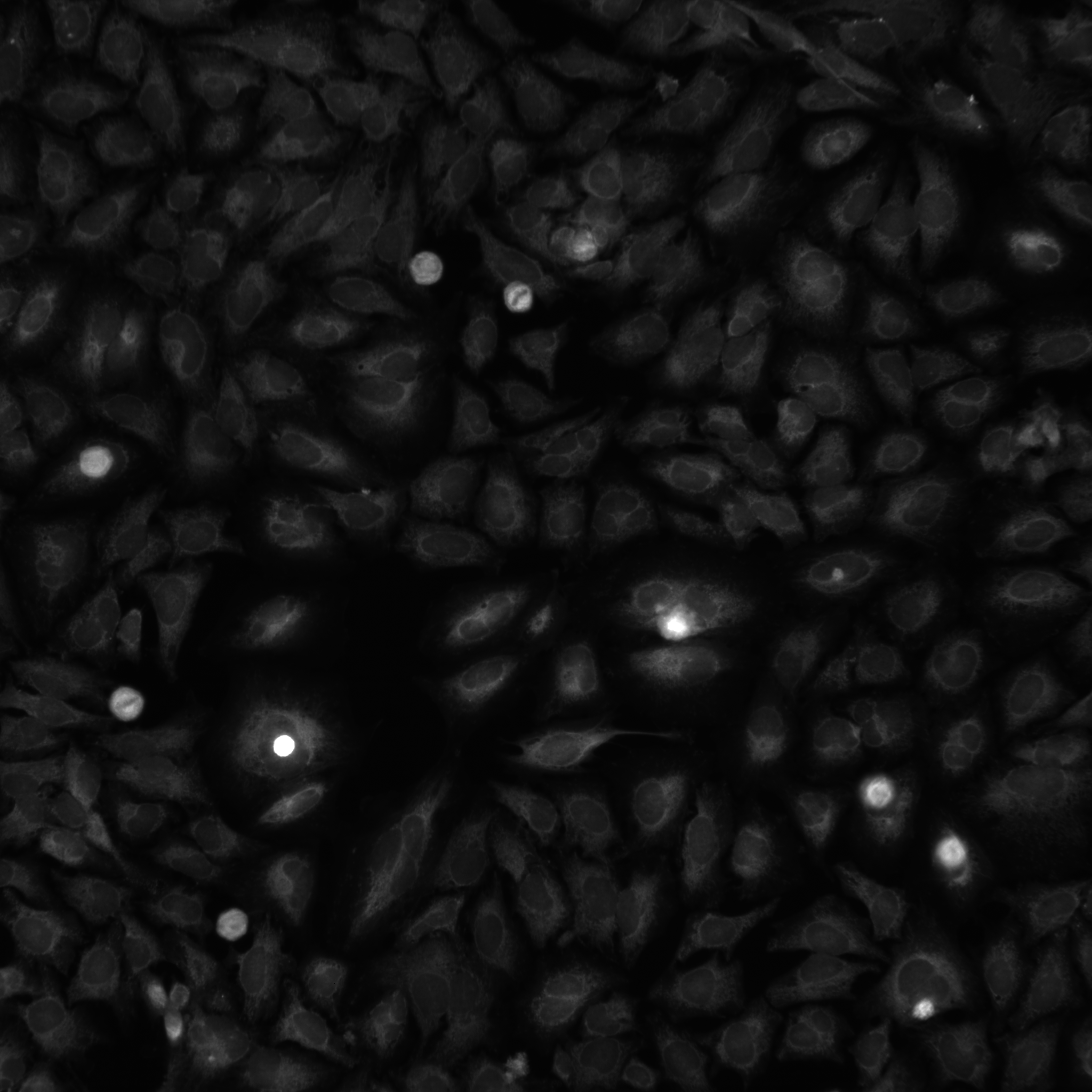

Supplement: Supplementary file 1 — Sample images and results. Sample datasets used in this paper (# 1 and #5 in table 2). The dataset includes input images of both dsRed and Cy5 channels and the corresponding cell segmentation. (ZIP 245,472 kb) [file 12859_2018_2375_MOESM1_ESM.zip › FYVE Hela 1/A - 5(fld 1 wv Red - Cy5).tif]

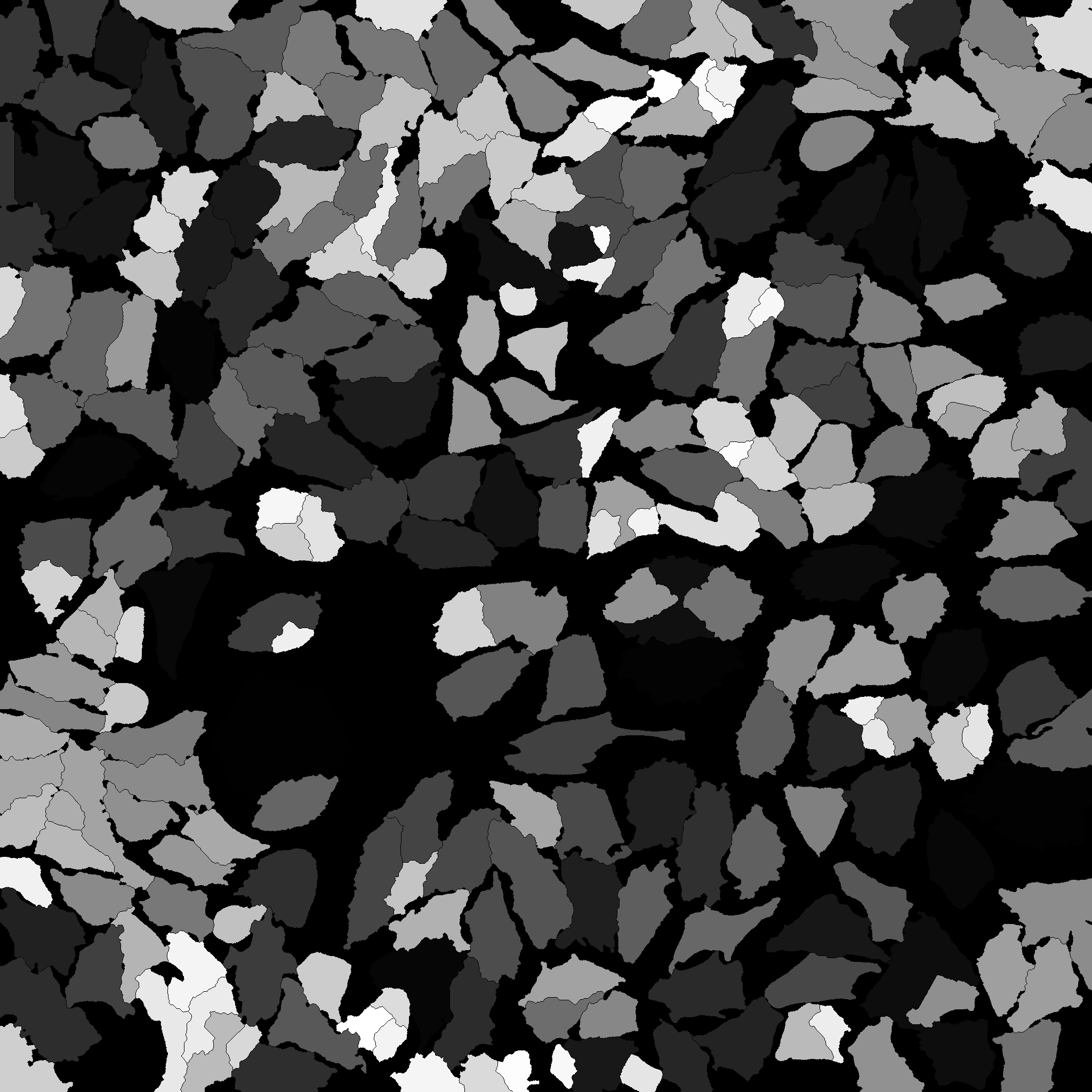

Supplement: Supplementary file 1 — Sample images and results. Sample datasets used in this paper (# 1 and #5 in table 2). The dataset includes input images of both dsRed and Cy5 channels and the corresponding cell segmentation. (ZIP 245,472 kb) [file 12859_2018_2375_MOESM1_ESM.zip › FYVE Hela 1/A - 5(fld 1 wv Red - Cy5)_cellseg_label.tif]

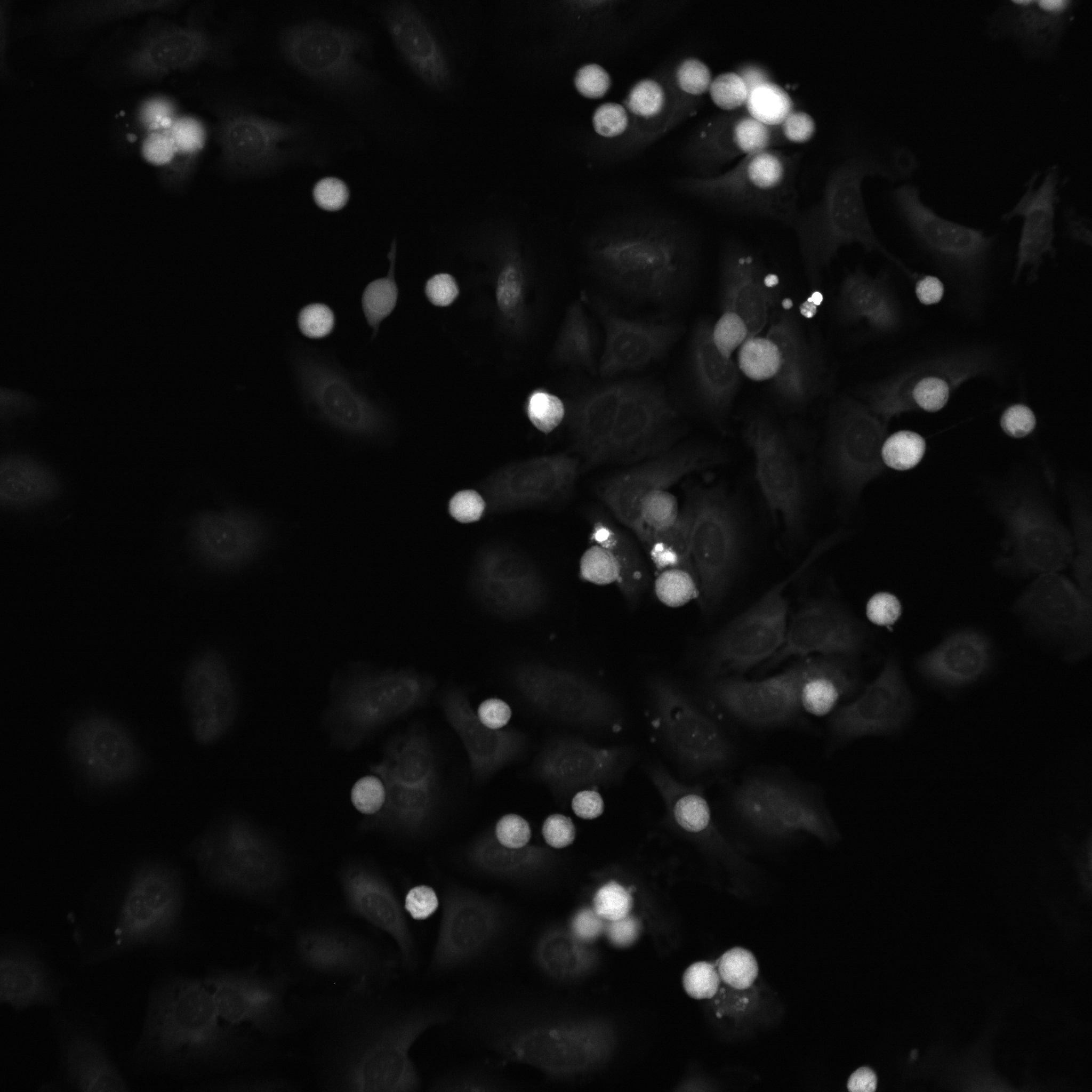

Supplement: Supplementary file 1 — Sample images and results. Sample datasets used in this paper (# 1 and #5 in table 2). The dataset includes input images of both dsRed and Cy5 channels and the corresponding cell segmentation. (ZIP 245,472 kb) [file 12859_2018_2375_MOESM1_ESM.zip › FYVE Hela 1/A - 8(fld 1 wv Green - dsRed).tif]

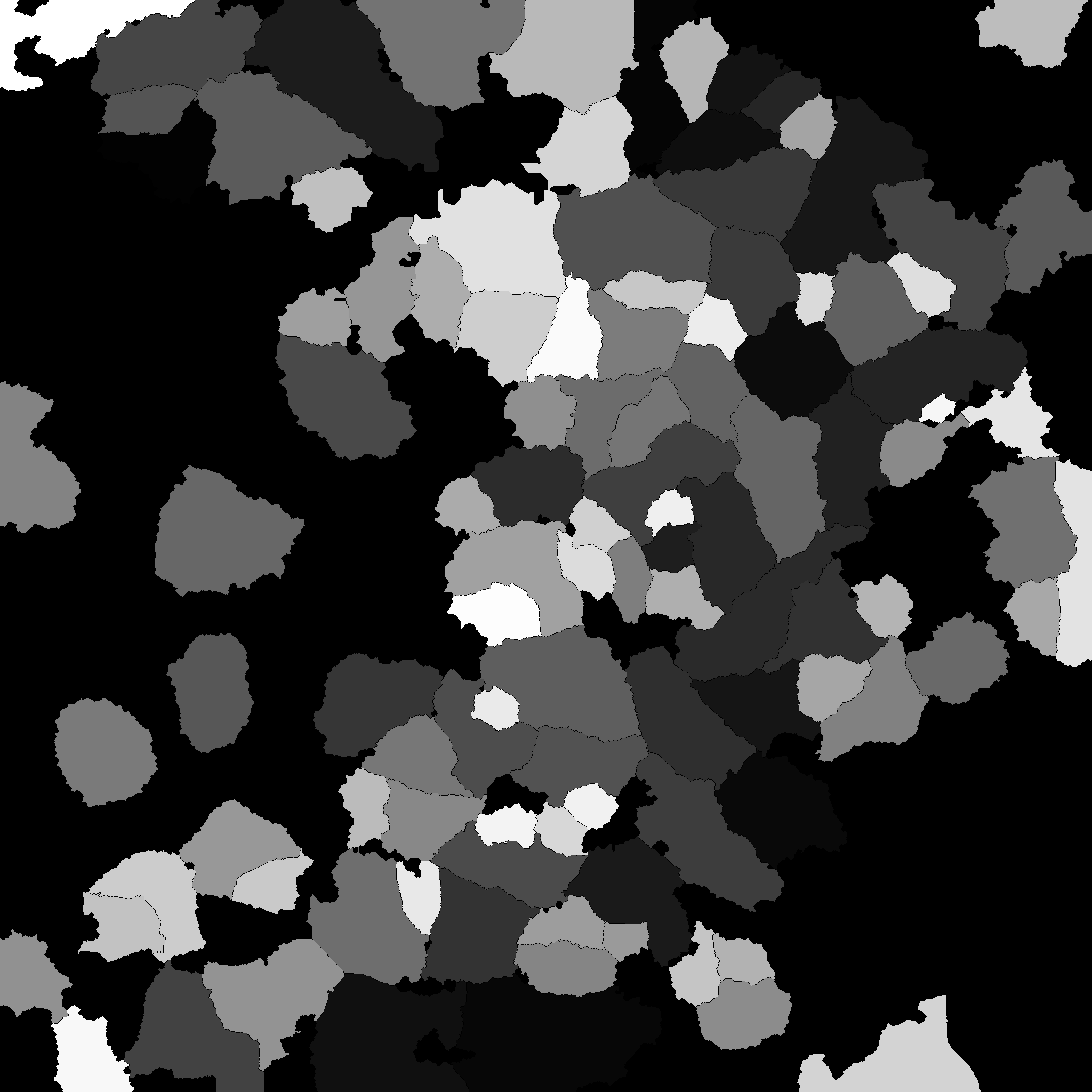

Supplement: Supplementary file 1 — Sample images and results. Sample datasets used in this paper (# 1 and #5 in table 2). The dataset includes input images of both dsRed and Cy5 channels and the corresponding cell segmentation. (ZIP 245,472 kb) [file 12859_2018_2375_MOESM1_ESM.zip › FYVE Hela 1/A - 8(fld 1 wv Green - dsRed)_cellseg_label.tif]

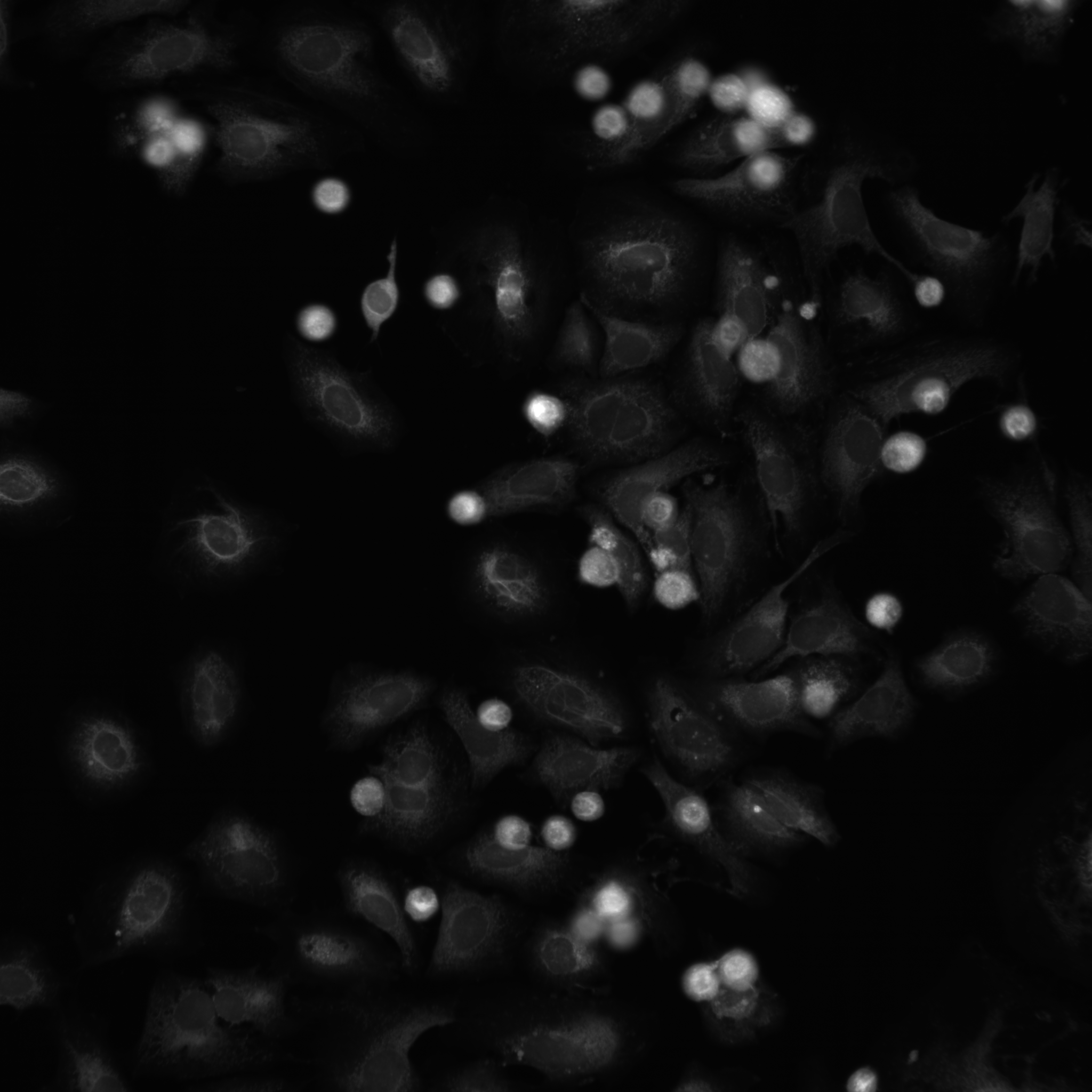

Supplement: Supplementary file 1 — Sample images and results. Sample datasets used in this paper (# 1 and #5 in table 2). The dataset includes input images of both dsRed and Cy5 channels and the corresponding cell segmentation. (ZIP 245,472 kb) [file 12859_2018_2375_MOESM1_ESM.zip › FYVE Hela 1/A - 8(fld 1 wv Red - Cy5).tif]

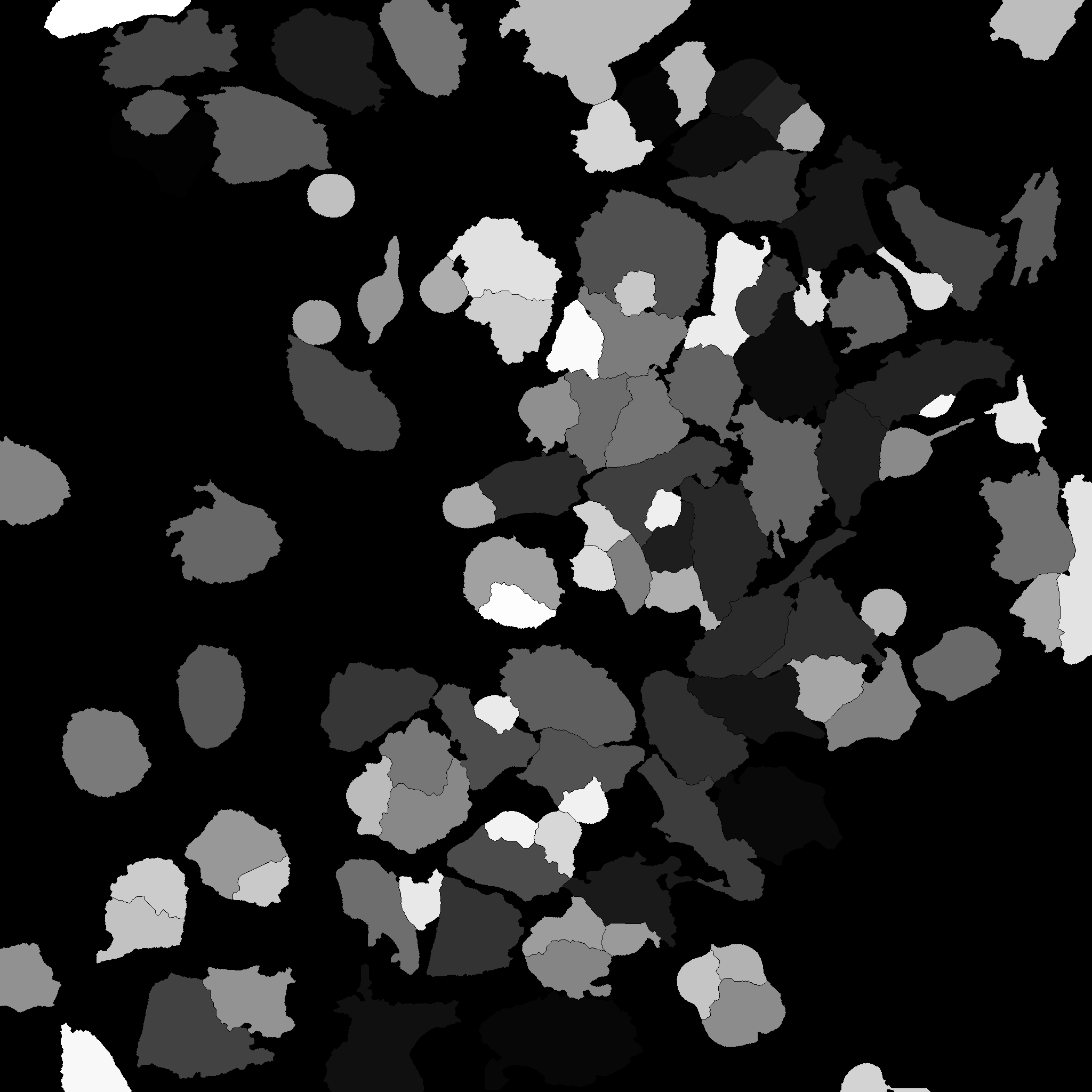

Supplement: Supplementary file 1 — Sample images and results. Sample datasets used in this paper (# 1 and #5 in table 2). The dataset includes input images of both dsRed and Cy5 channels and the corresponding cell segmentation. (ZIP 245,472 kb) [file 12859_2018_2375_MOESM1_ESM.zip › FYVE Hela 1/A - 8(fld 1 wv Red - Cy5)_cellseg_label.tif]

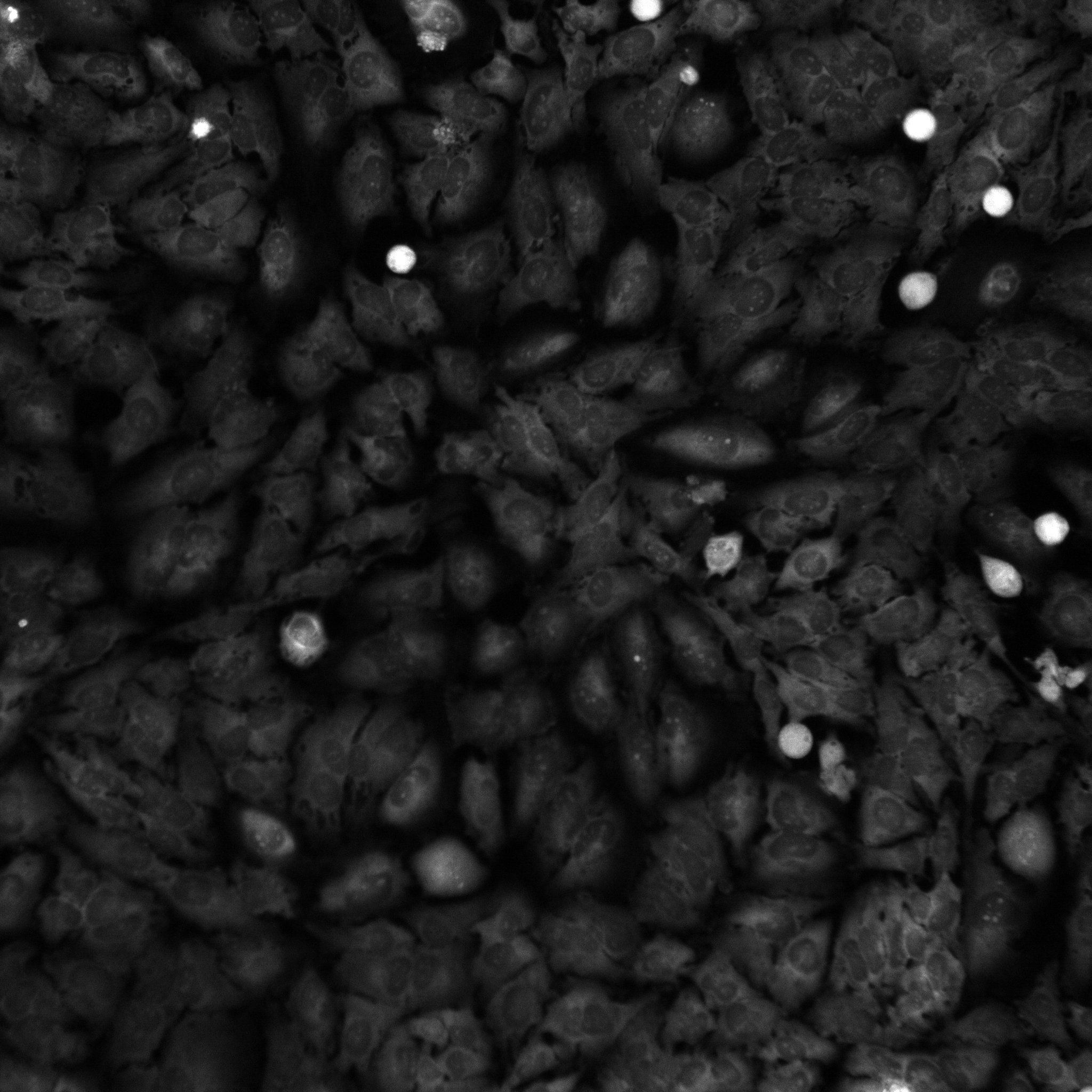

Supplement: Supplementary file 1 — Sample images and results. Sample datasets used in this paper (# 1 and #5 in table 2). The dataset includes input images of both dsRed and Cy5 channels and the corresponding cell segmentation. (ZIP 245,472 kb) [file 12859_2018_2375_MOESM1_ESM.zip › FYVE Hela 1/B - 1(fld 1 wv Green - dsRed).tif]

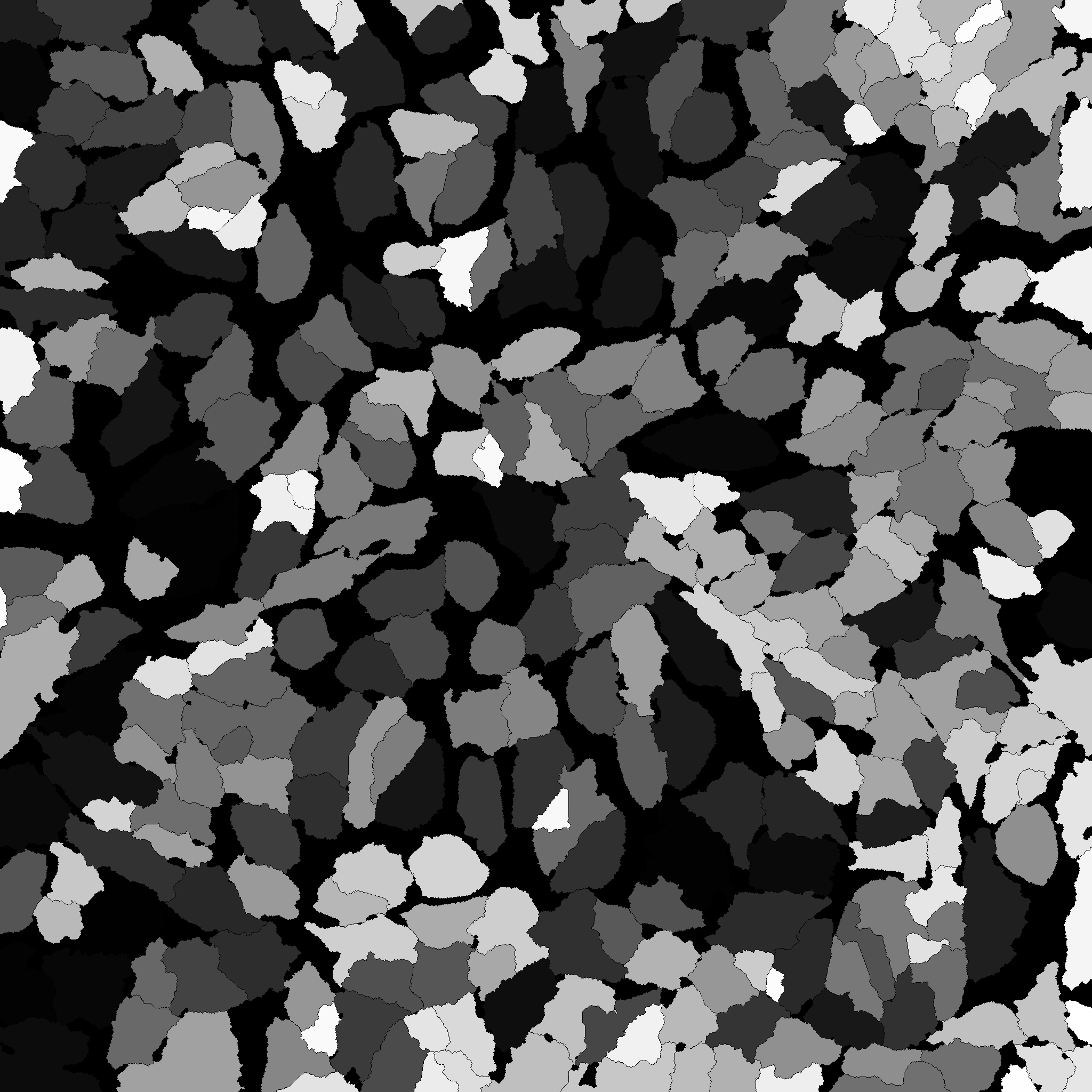

Supplement: Supplementary file 1 — Sample images and results. Sample datasets used in this paper (# 1 and #5 in table 2). The dataset includes input images of both dsRed and Cy5 channels and the corresponding cell segmentation. (ZIP 245,472 kb) [file 12859_2018_2375_MOESM1_ESM.zip › FYVE Hela 1/B - 1(fld 1 wv Green - dsRed)_cellseg_label.tif]

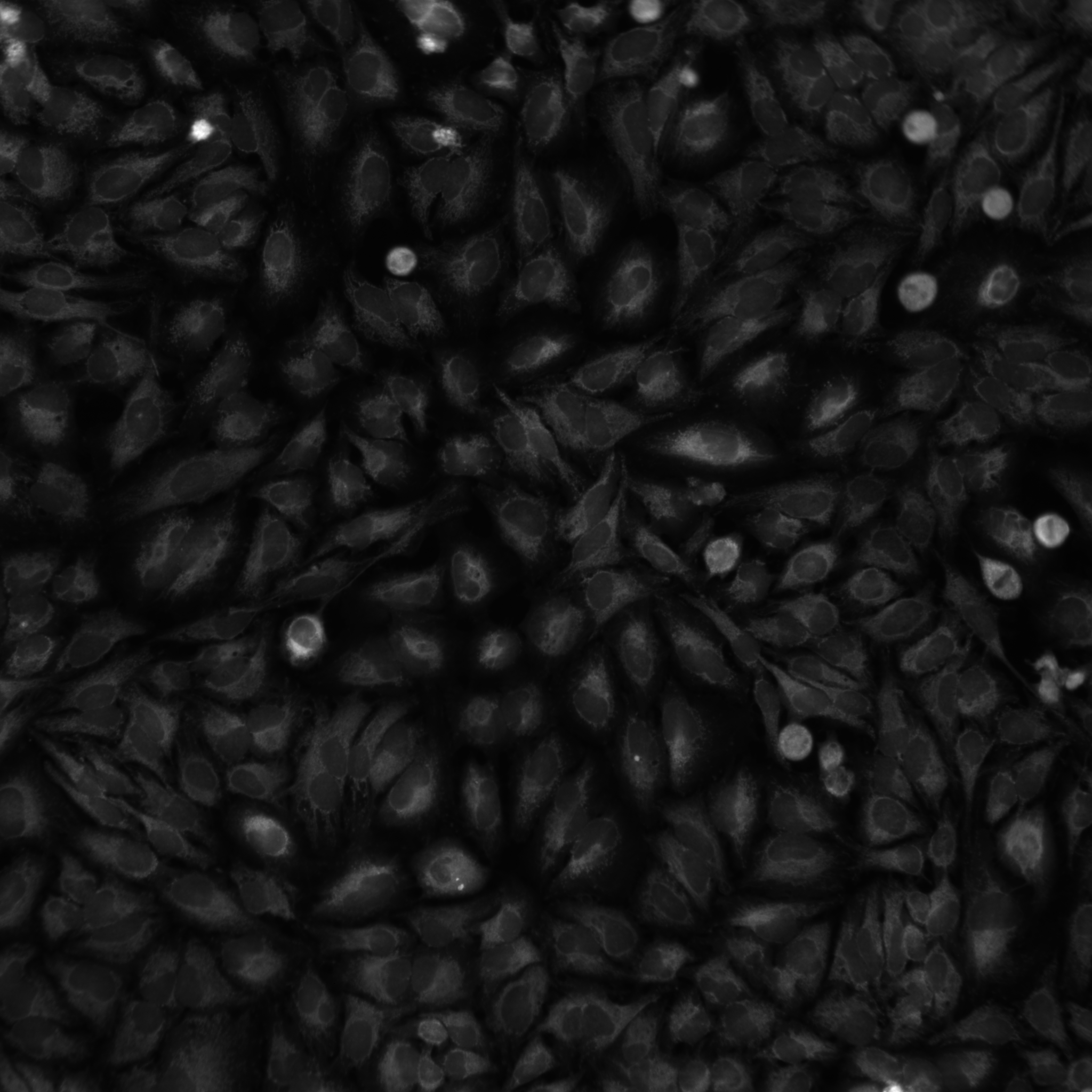

Supplement: Supplementary file 1 — Sample images and results. Sample datasets used in this paper (# 1 and #5 in table 2). The dataset includes input images of both dsRed and Cy5 channels and the corresponding cell segmentation. (ZIP 245,472 kb) [file 12859_2018_2375_MOESM1_ESM.zip › FYVE Hela 1/B - 1(fld 1 wv Red - Cy5).tif]

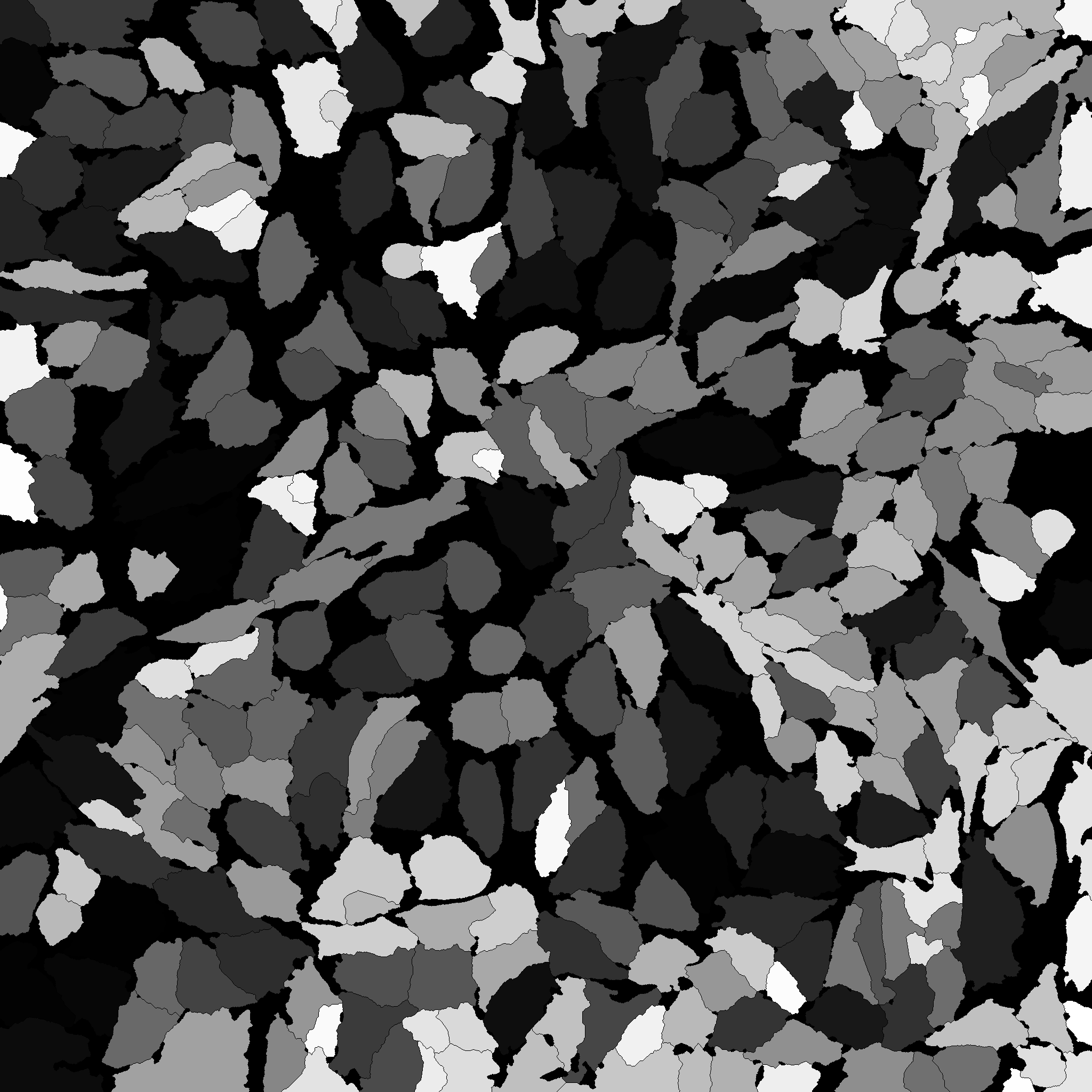

Supplement: Supplementary file 1 — Sample images and results. Sample datasets used in this paper (# 1 and #5 in table 2). The dataset includes input images of both dsRed and Cy5 channels and the corresponding cell segmentation. (ZIP 245,472 kb) [file 12859_2018_2375_MOESM1_ESM.zip › FYVE Hela 1/B - 1(fld 1 wv Red - Cy5)_cellseg_label.tif]

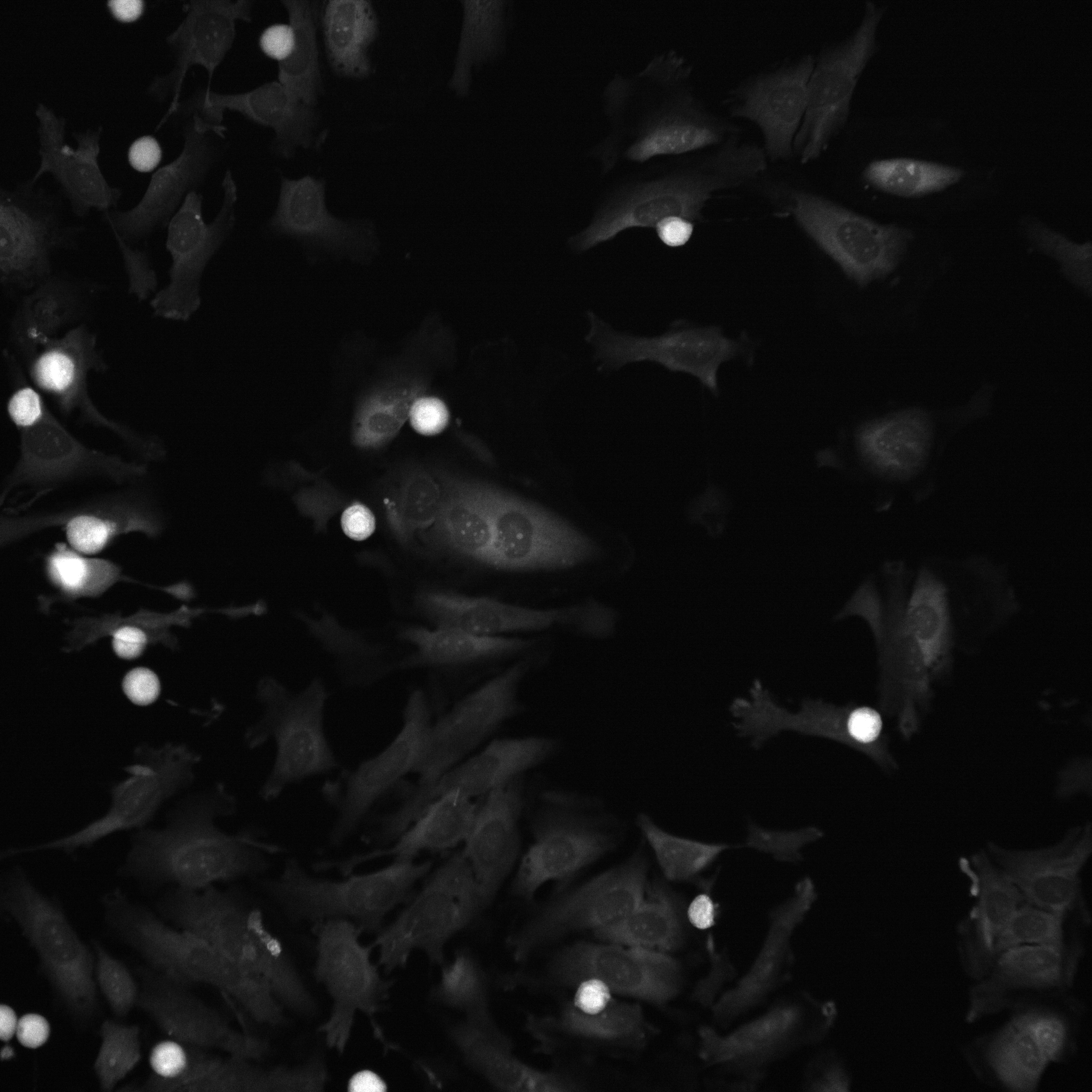

Supplement: Supplementary file 1 — Sample images and results. Sample datasets used in this paper (# 1 and #5 in table 2). The dataset includes input images of both dsRed and Cy5 channels and the corresponding cell segmentation. (ZIP 245,472 kb) [file 12859_2018_2375_MOESM1_ESM.zip › FYVE Hela 1/B - 10(fld 1 wv Green - dsRed).tif]

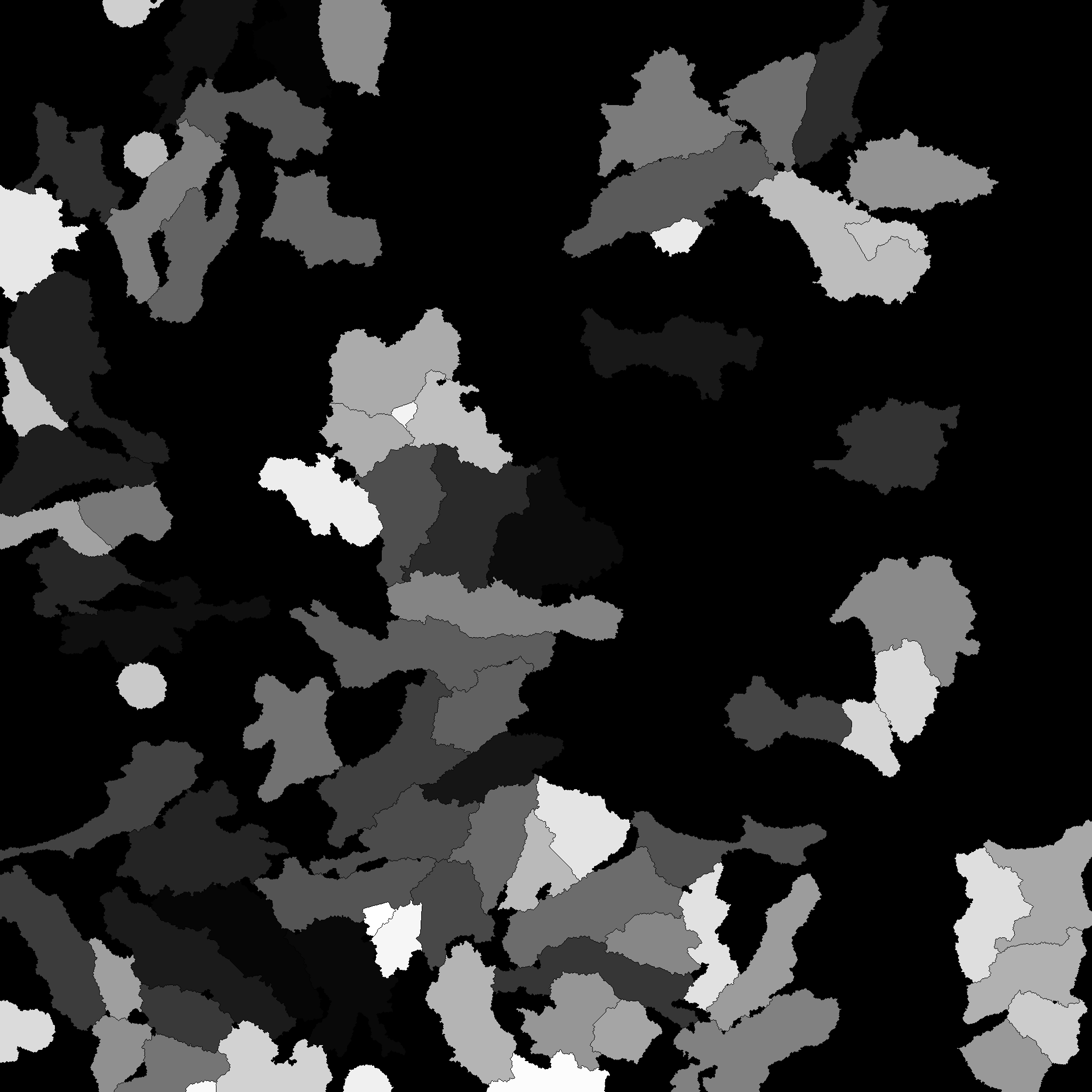

Supplement: Supplementary file 1 — Sample images and results. Sample datasets used in this paper (# 1 and #5 in table 2). The dataset includes input images of both dsRed and Cy5 channels and the corresponding cell segmentation. (ZIP 245,472 kb) [file 12859_2018_2375_MOESM1_ESM.zip › FYVE Hela 1/B - 10(fld 1 wv Green - dsRed)_cellseg_label.tif]

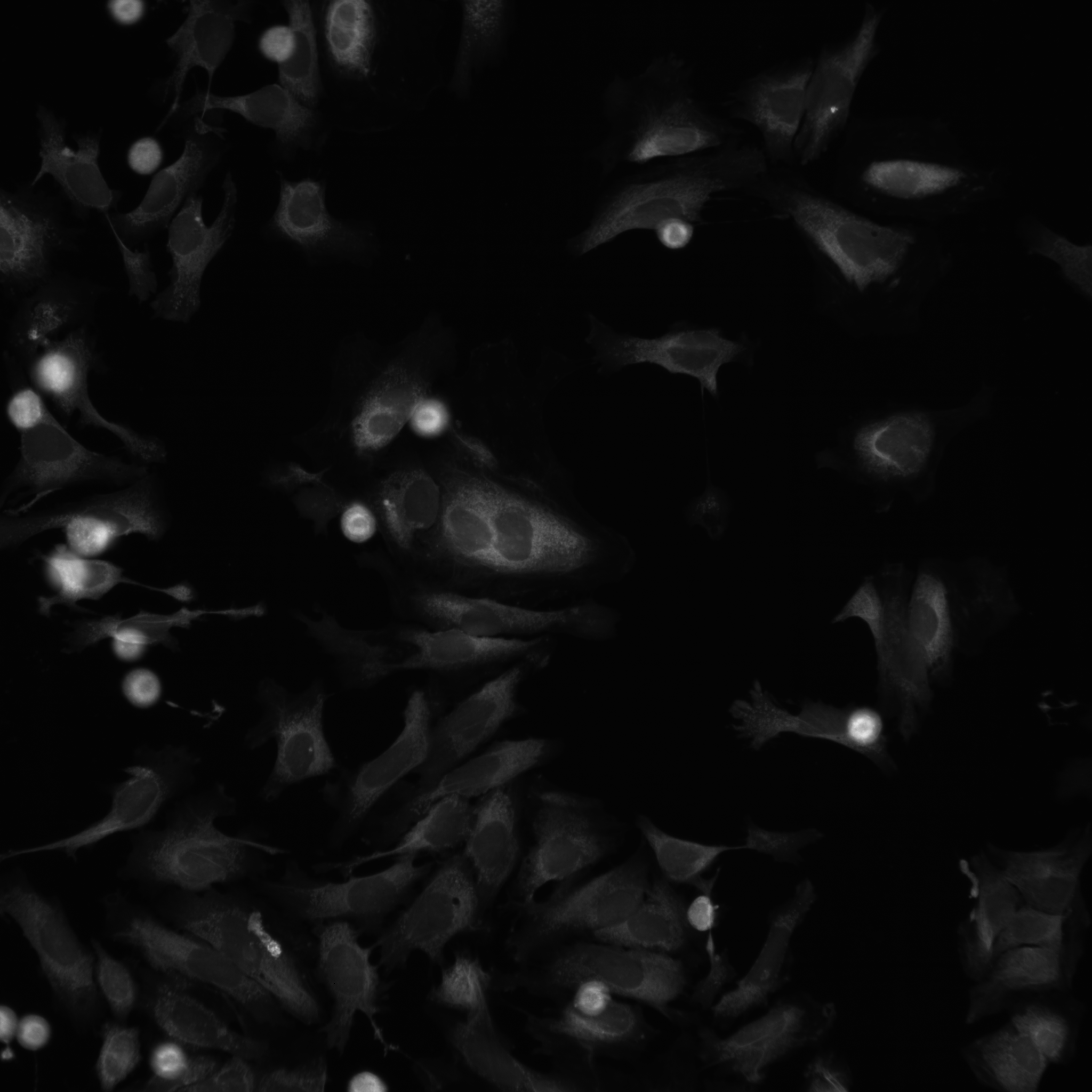

Supplement: Supplementary file 1 — Sample images and results. Sample datasets used in this paper (# 1 and #5 in table 2). The dataset includes input images of both dsRed and Cy5 channels and the corresponding cell segmentation. (ZIP 245,472 kb) [file 12859_2018_2375_MOESM1_ESM.zip › FYVE Hela 1/B - 10(fld 1 wv Red - Cy5).tif]

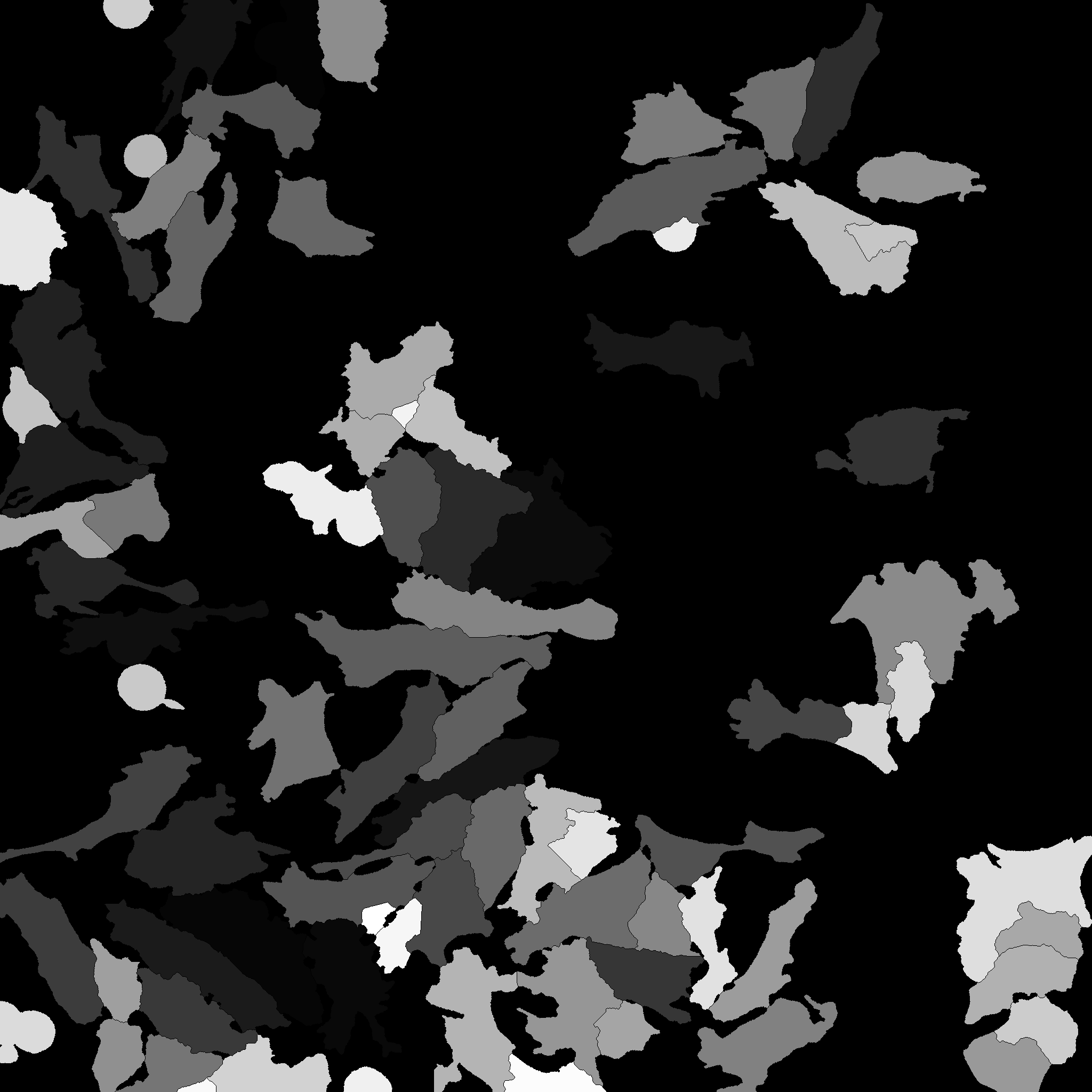

Supplement: Supplementary file 1 — Sample images and results. Sample datasets used in this paper (# 1 and #5 in table 2). The dataset includes input images of both dsRed and Cy5 channels and the corresponding cell segmentation. (ZIP 245,472 kb) [file 12859_2018_2375_MOESM1_ESM.zip › FYVE Hela 1/B - 10(fld 1 wv Red - Cy5)_cellseg_label.tif]

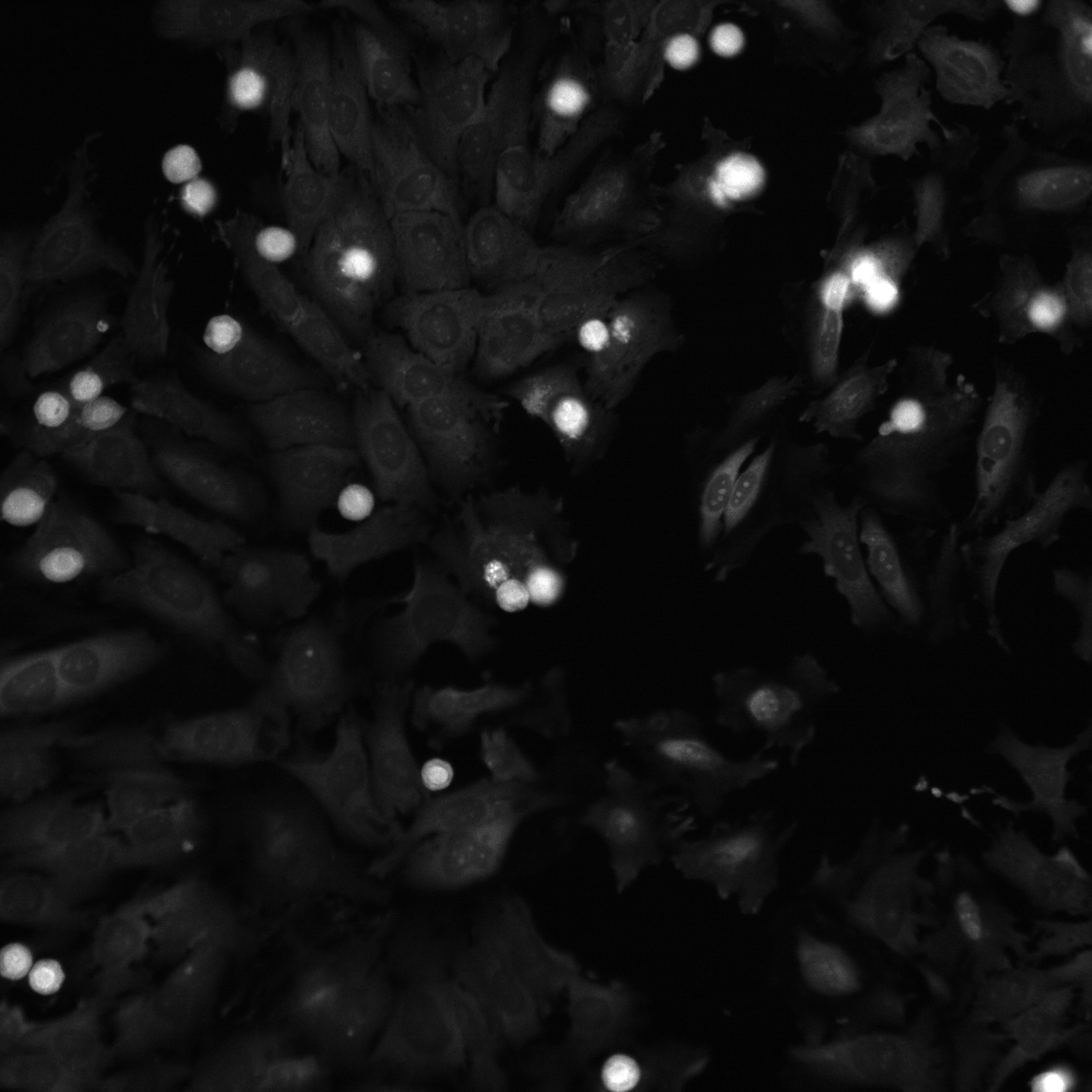

Supplement: Supplementary file 1 — Sample images and results. Sample datasets used in this paper (# 1 and #5 in table 2). The dataset includes input images of both dsRed and Cy5 channels and the corresponding cell segmentation. (ZIP 245,472 kb) [file 12859_2018_2375_MOESM1_ESM.zip › FYVE Hela 1/B - 11(fld 1 wv Green - dsRed).tif]

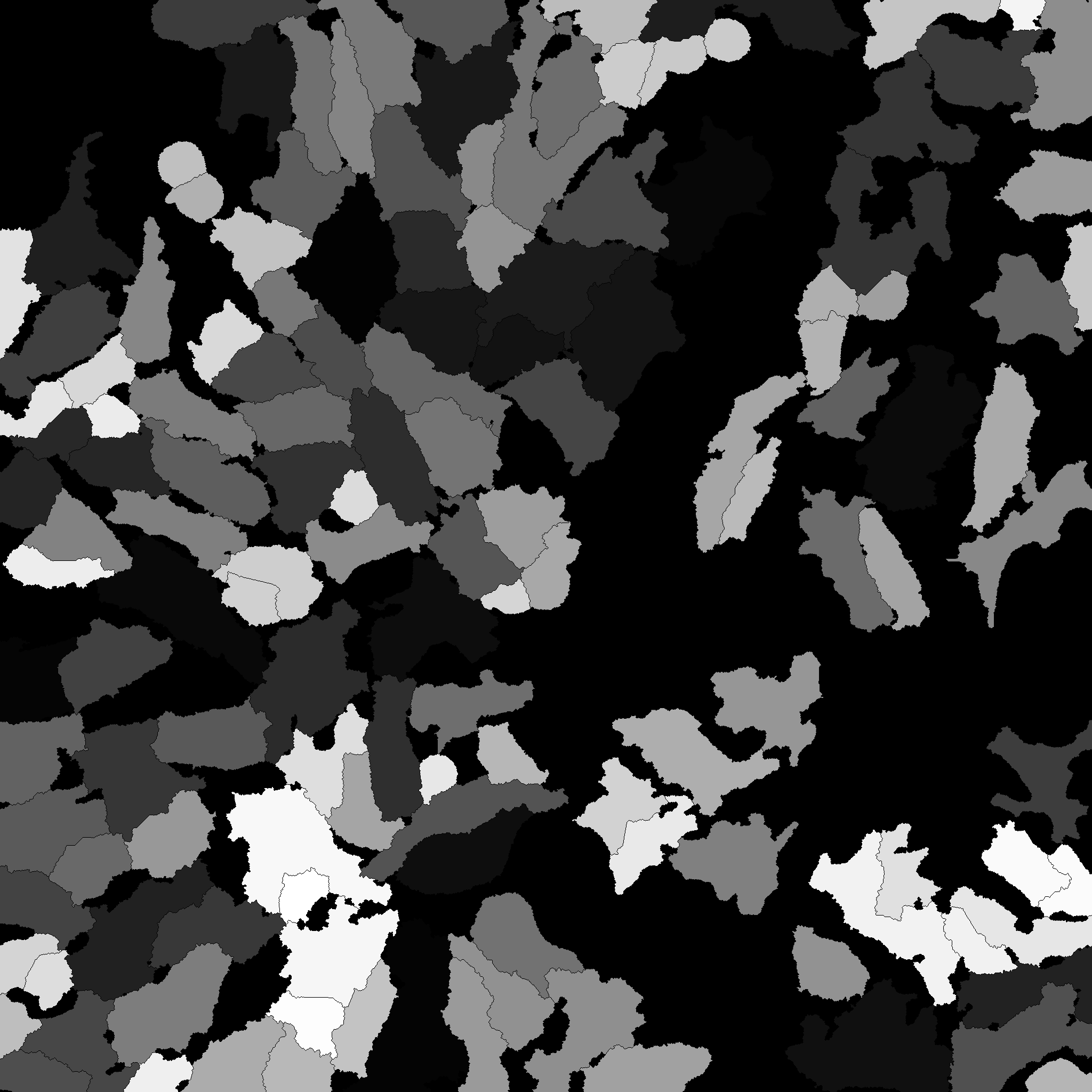

Supplement: Supplementary file 1 — Sample images and results. Sample datasets used in this paper (# 1 and #5 in table 2). The dataset includes input images of both dsRed and Cy5 channels and the corresponding cell segmentation. (ZIP 245,472 kb) [file 12859_2018_2375_MOESM1_ESM.zip › FYVE Hela 1/B - 11(fld 1 wv Green - dsRed)_cellseg_label.tif]

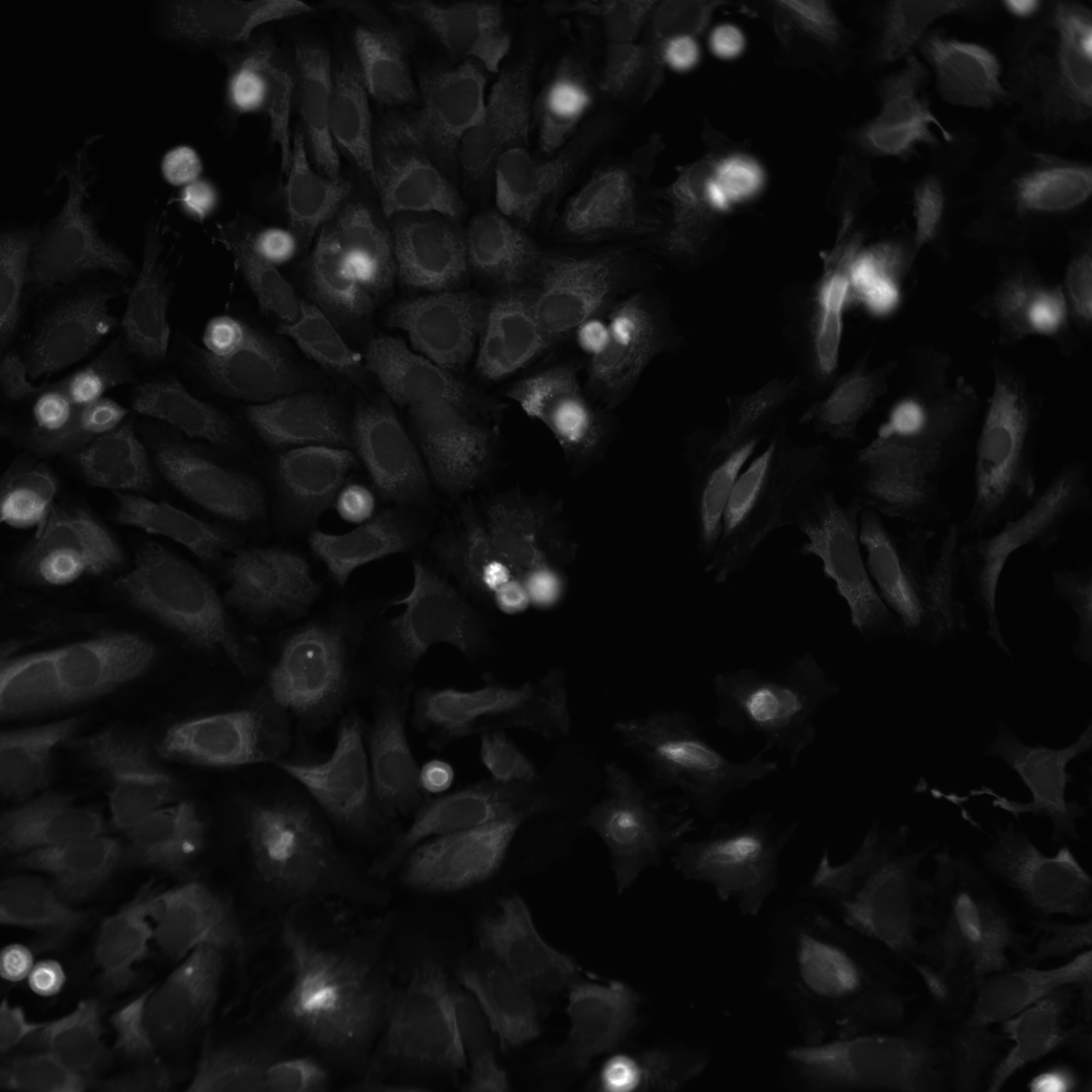

Supplement: Supplementary file 1 — Sample images and results. Sample datasets used in this paper (# 1 and #5 in table 2). The dataset includes input images of both dsRed and Cy5 channels and the corresponding cell segmentation. (ZIP 245,472 kb) [file 12859_2018_2375_MOESM1_ESM.zip › FYVE Hela 1/B - 11(fld 1 wv Red - Cy5).tif]

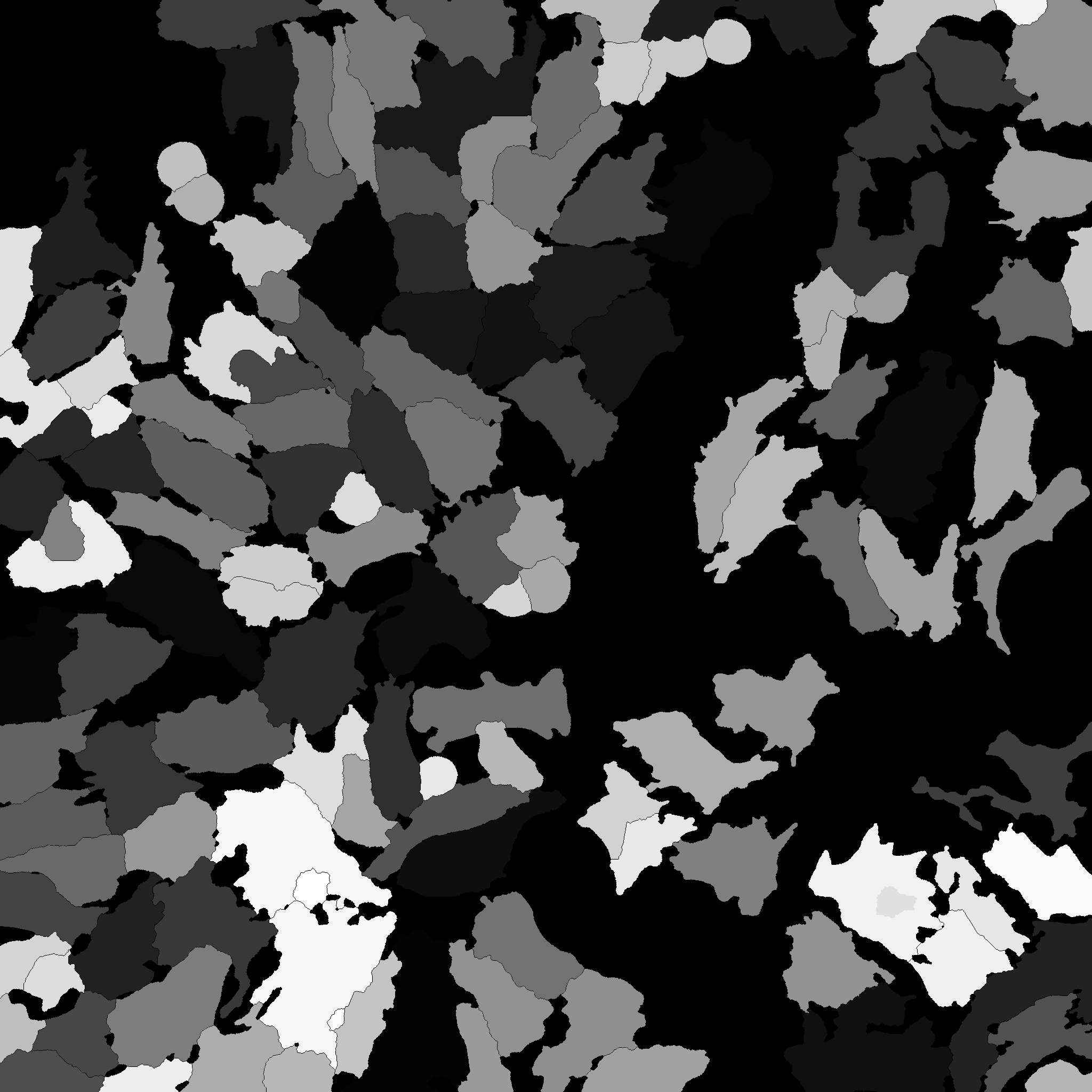

Supplement: Supplementary file 1 — Sample images and results. Sample datasets used in this paper (# 1 and #5 in table 2). The dataset includes input images of both dsRed and Cy5 channels and the corresponding cell segmentation. (ZIP 245,472 kb) [file 12859_2018_2375_MOESM1_ESM.zip › FYVE Hela 1/B - 11(fld 1 wv Red - Cy5)_cellseg_label.tif]

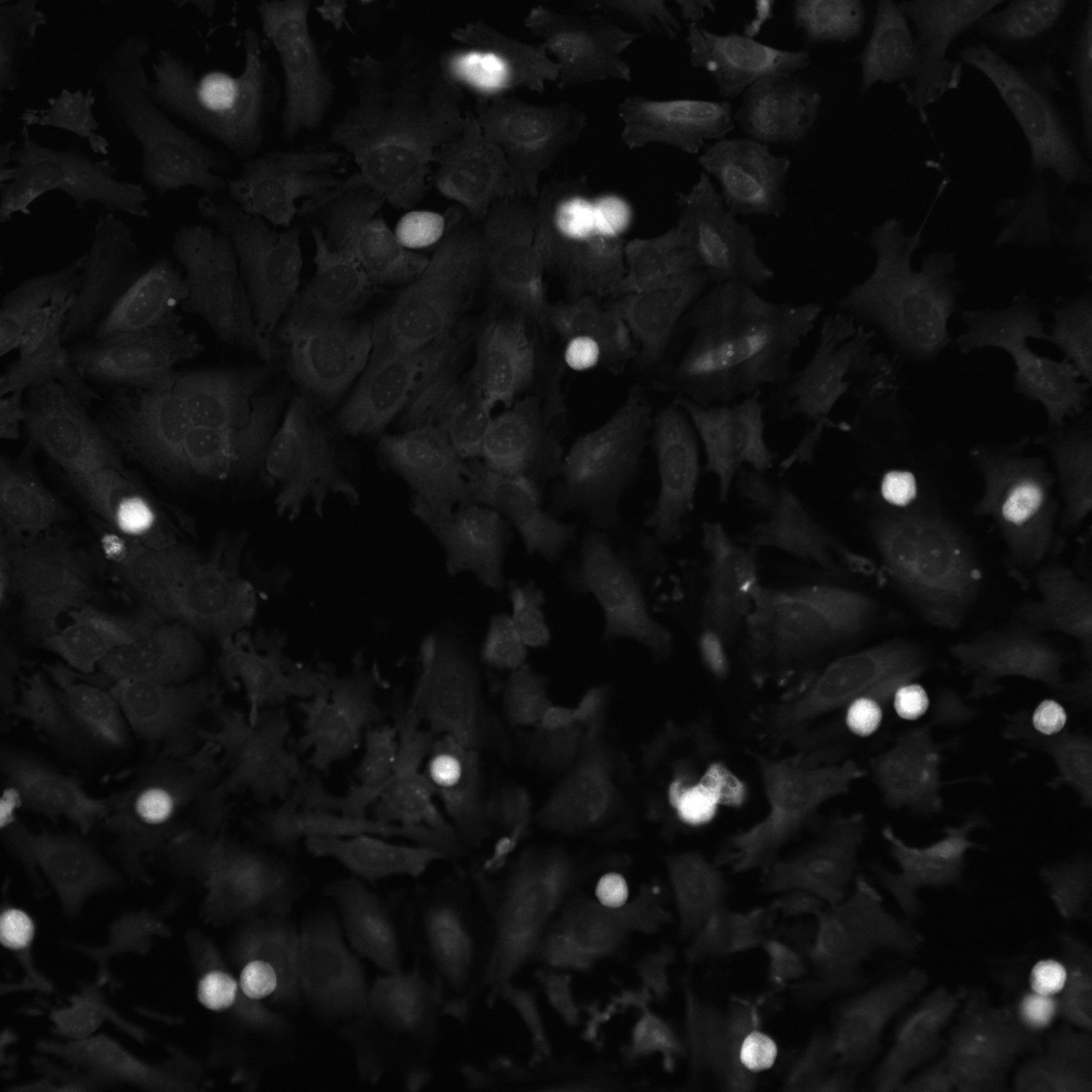

Supplement: Supplementary file 1 — Sample images and results. Sample datasets used in this paper (# 1 and #5 in table 2). The dataset includes input images of both dsRed and Cy5 channels and the corresponding cell segmentation. (ZIP 245,472 kb) [file 12859_2018_2375_MOESM1_ESM.zip › FYVE Hela 1/B - 12(fld 1 wv Green - dsRed).tif]

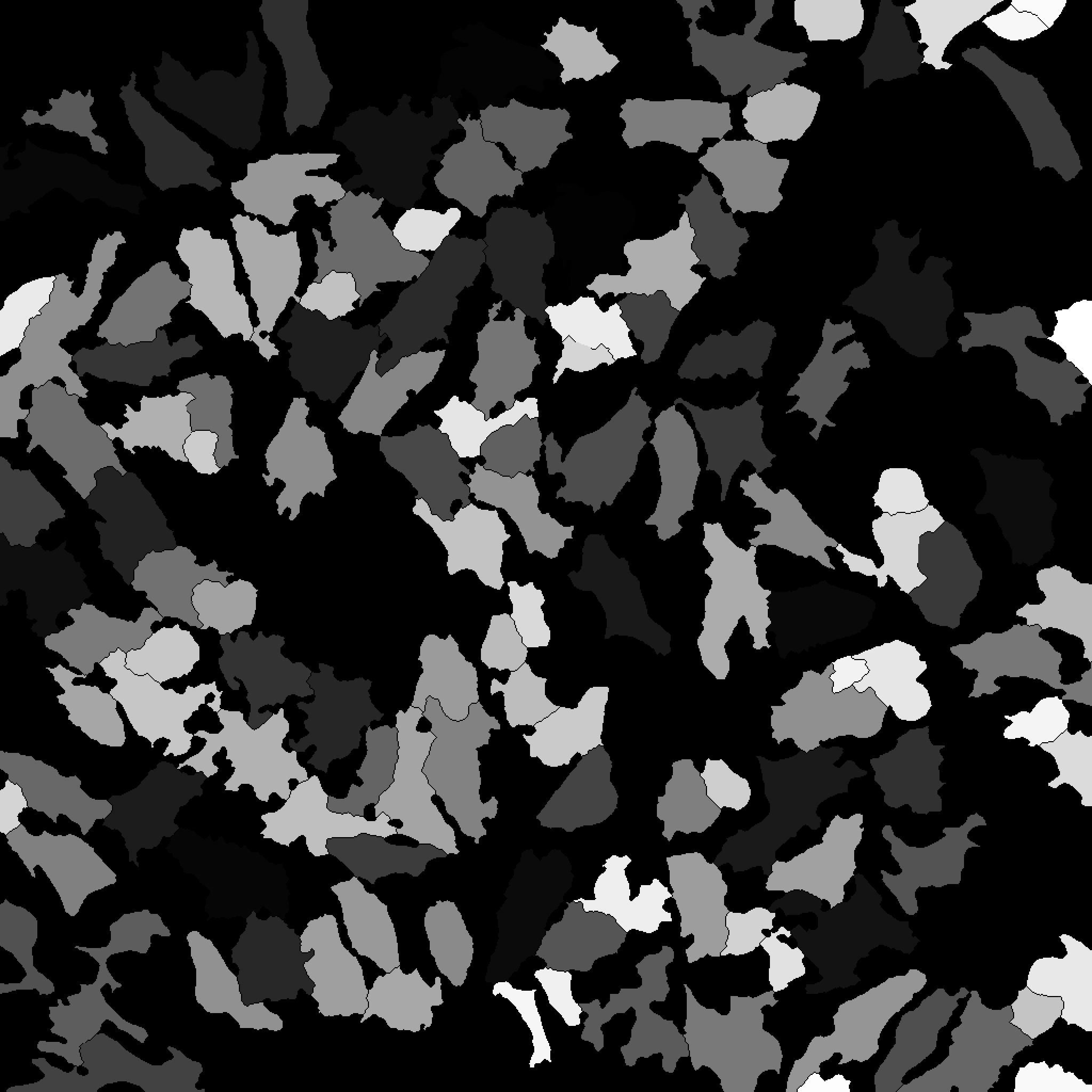

Supplement: Supplementary file 1 — Sample images and results. Sample datasets used in this paper (# 1 and #5 in table 2). The dataset includes input images of both dsRed and Cy5 channels and the corresponding cell segmentation. (ZIP 245,472 kb) [file 12859_2018_2375_MOESM1_ESM.zip › FYVE Hela 1/B - 12(fld 1 wv Green - dsRed)_cellseg_label.tif]

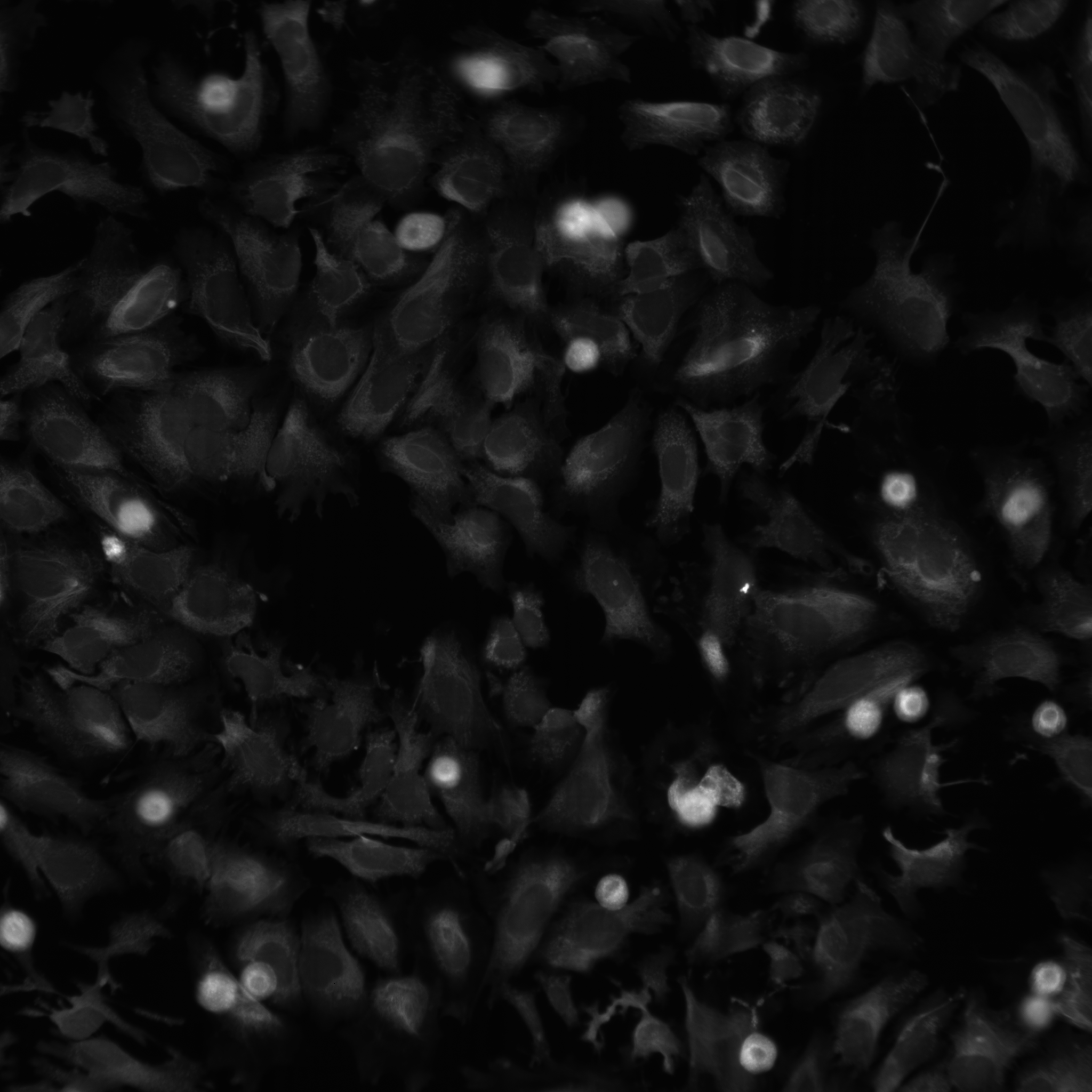

Supplement: Supplementary file 1 — Sample images and results. Sample datasets used in this paper (# 1 and #5 in table 2). The dataset includes input images of both dsRed and Cy5 channels and the corresponding cell segmentation. (ZIP 245,472 kb) [file 12859_2018_2375_MOESM1_ESM.zip › FYVE Hela 1/B - 12(fld 1 wv Red - Cy5).tif]

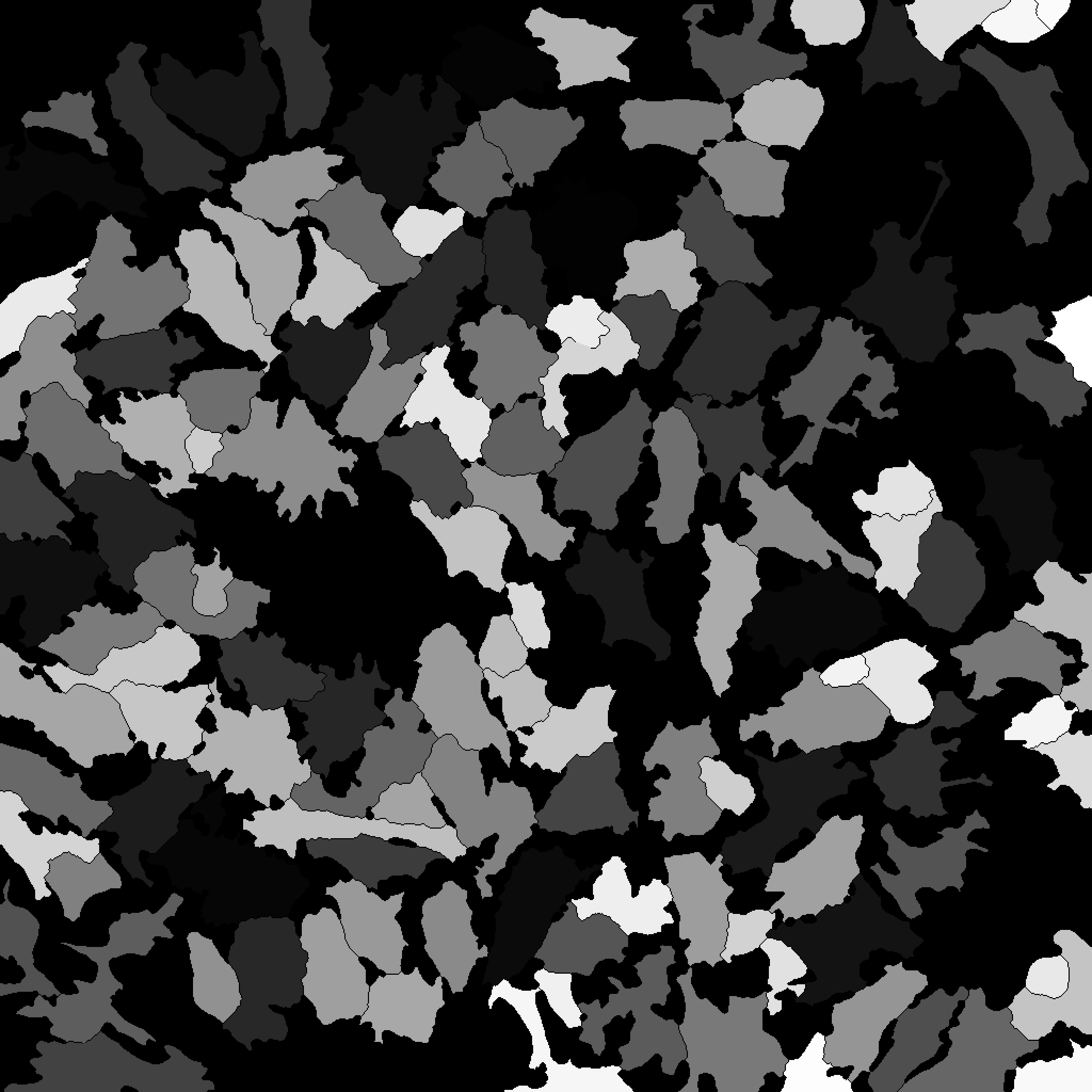

Supplement: Supplementary file 1 — Sample images and results. Sample datasets used in this paper (# 1 and #5 in table 2). The dataset includes input images of both dsRed and Cy5 channels and the corresponding cell segmentation. (ZIP 245,472 kb) [file 12859_2018_2375_MOESM1_ESM.zip › FYVE Hela 1/B - 12(fld 1 wv Red - Cy5)_cellseg_label.tif]

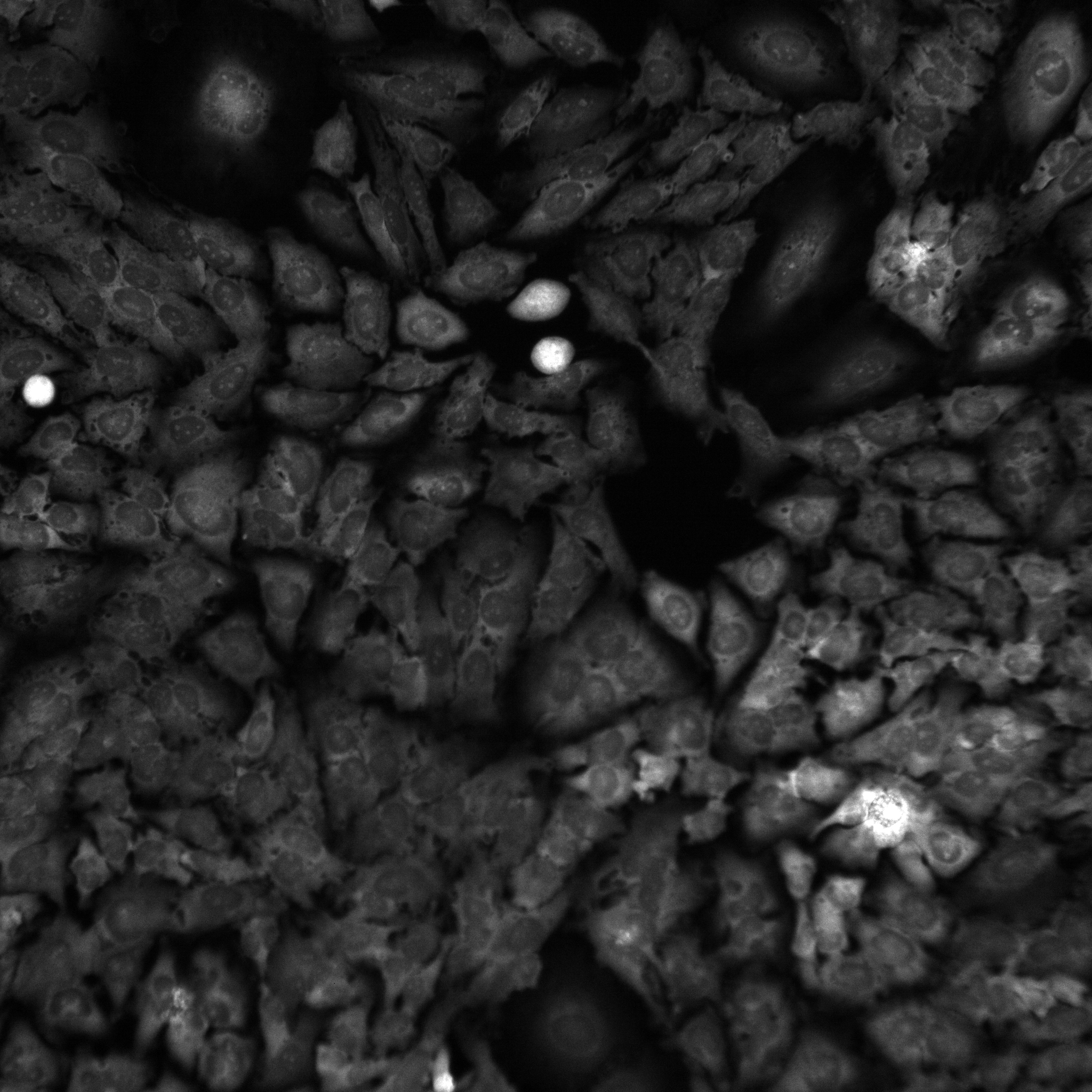

Supplement: Supplementary file 1 — Sample images and results. Sample datasets used in this paper (# 1 and #5 in table 2). The dataset includes input images of both dsRed and Cy5 channels and the corresponding cell segmentation. (ZIP 245,472 kb) [file 12859_2018_2375_MOESM1_ESM.zip › FYVE Hela 1/B - 2(fld 1 wv Green - dsRed).tif]

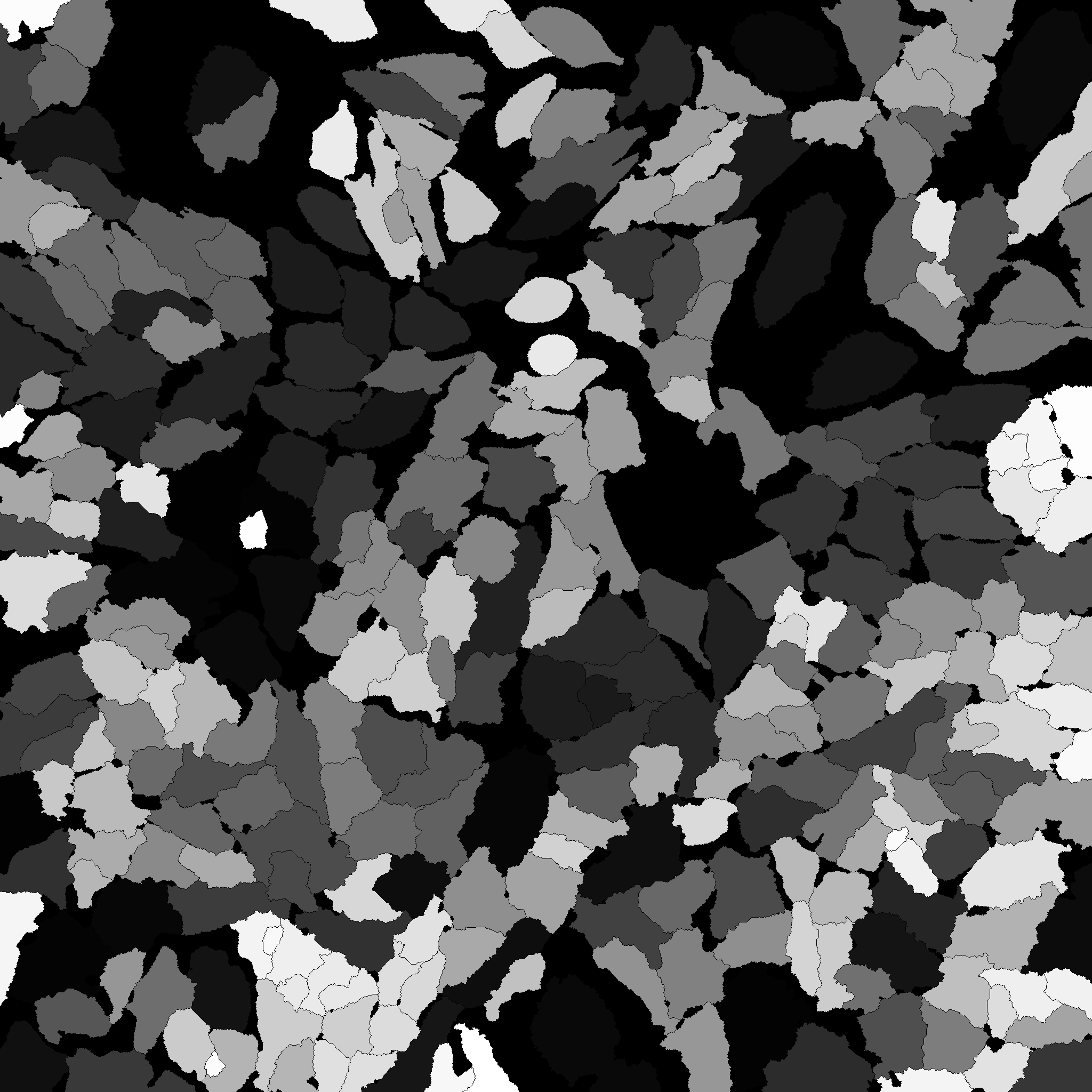

Supplement: Supplementary file 1 — Sample images and results. Sample datasets used in this paper (# 1 and #5 in table 2). The dataset includes input images of both dsRed and Cy5 channels and the corresponding cell segmentation. (ZIP 245,472 kb) [file 12859_2018_2375_MOESM1_ESM.zip › FYVE Hela 1/B - 2(fld 1 wv Green - dsRed)_cellseg_label.tif]

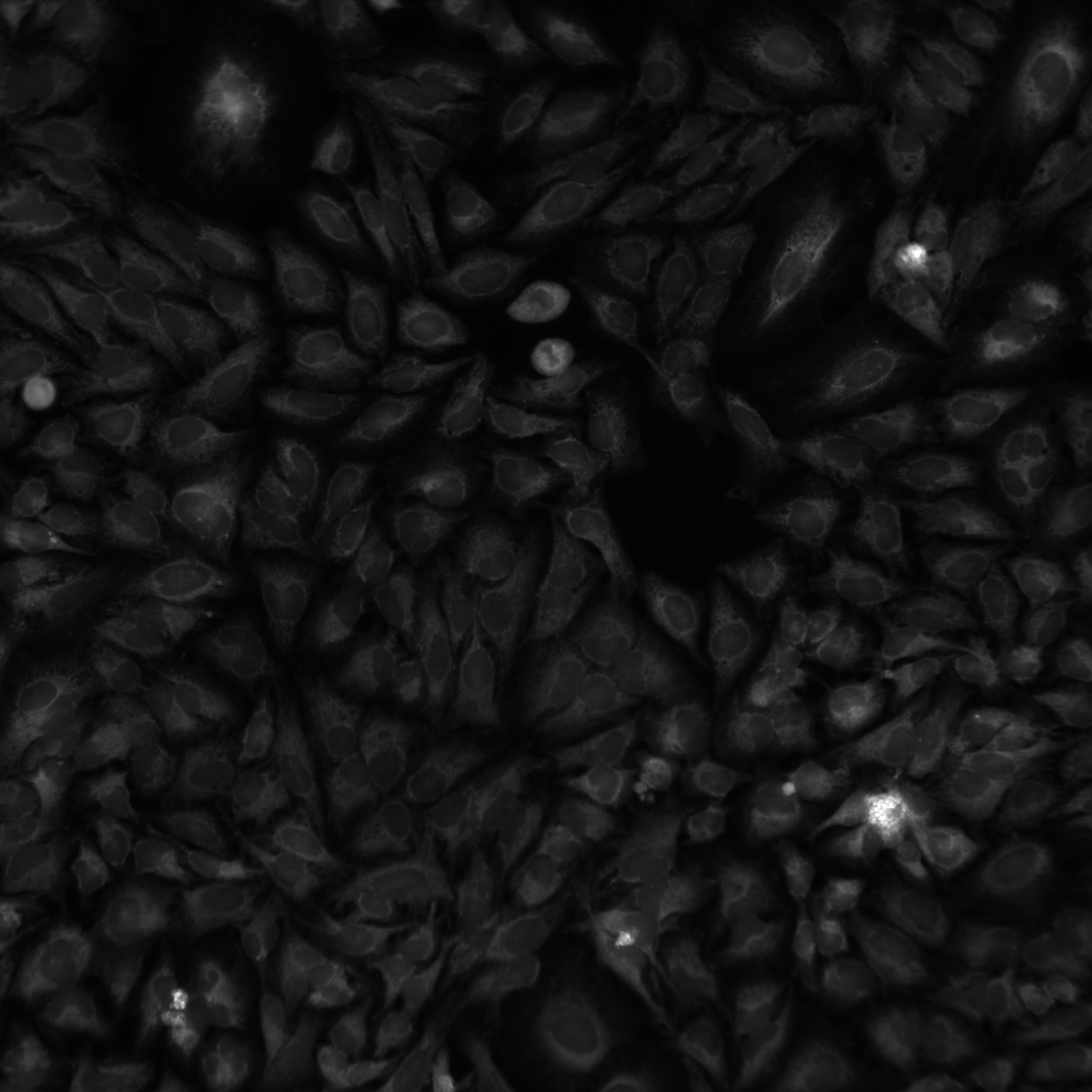

Supplement: Supplementary file 1 — Sample images and results. Sample datasets used in this paper (# 1 and #5 in table 2). The dataset includes input images of both dsRed and Cy5 channels and the corresponding cell segmentation. (ZIP 245,472 kb) [file 12859_2018_2375_MOESM1_ESM.zip › FYVE Hela 1/B - 2(fld 1 wv Red - Cy5).tif]

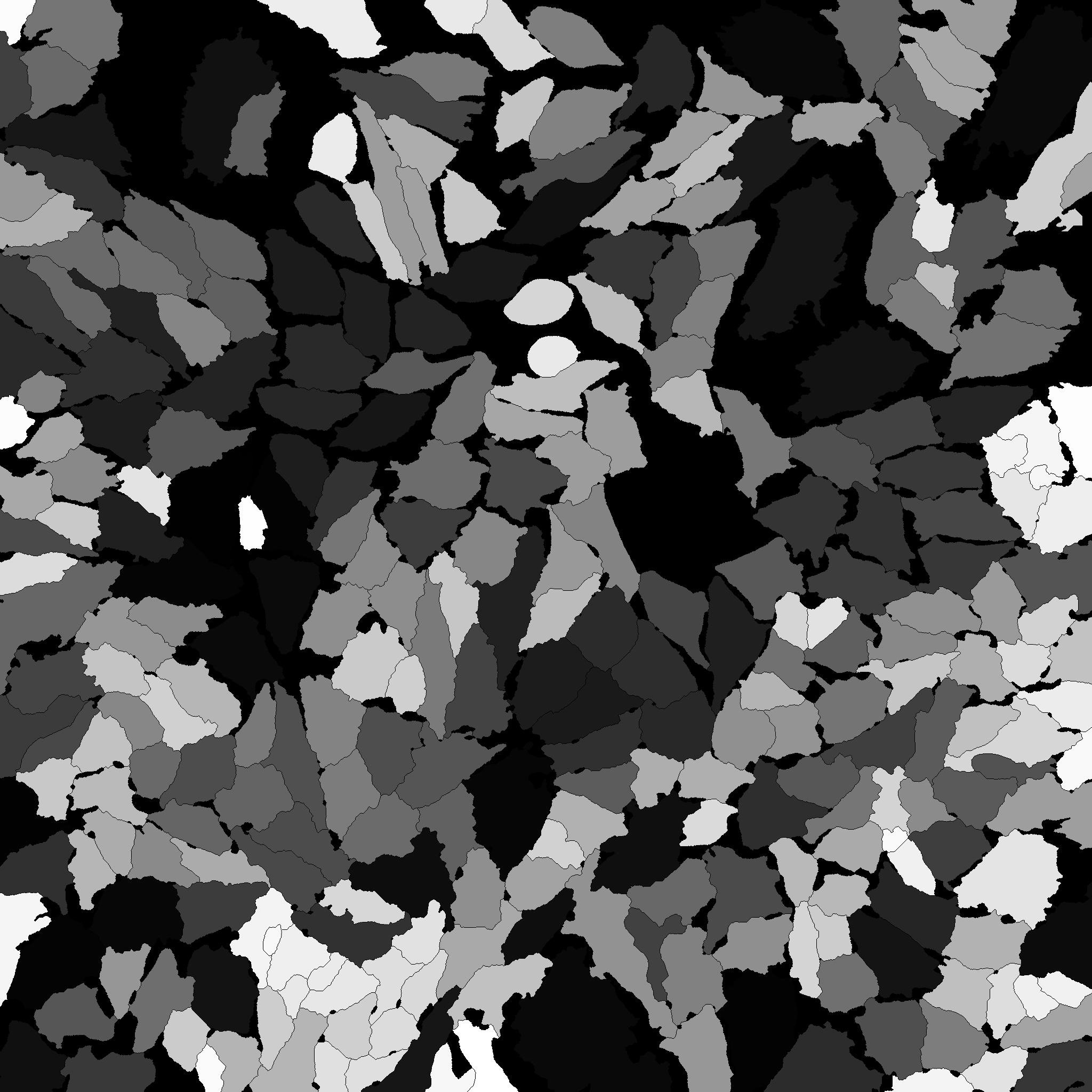

Supplement: Supplementary file 1 — Sample images and results. Sample datasets used in this paper (# 1 and #5 in table 2). The dataset includes input images of both dsRed and Cy5 channels and the corresponding cell segmentation. (ZIP 245,472 kb) [file 12859_2018_2375_MOESM1_ESM.zip › FYVE Hela 1/B - 2(fld 1 wv Red - Cy5)_cellseg_label.tif]

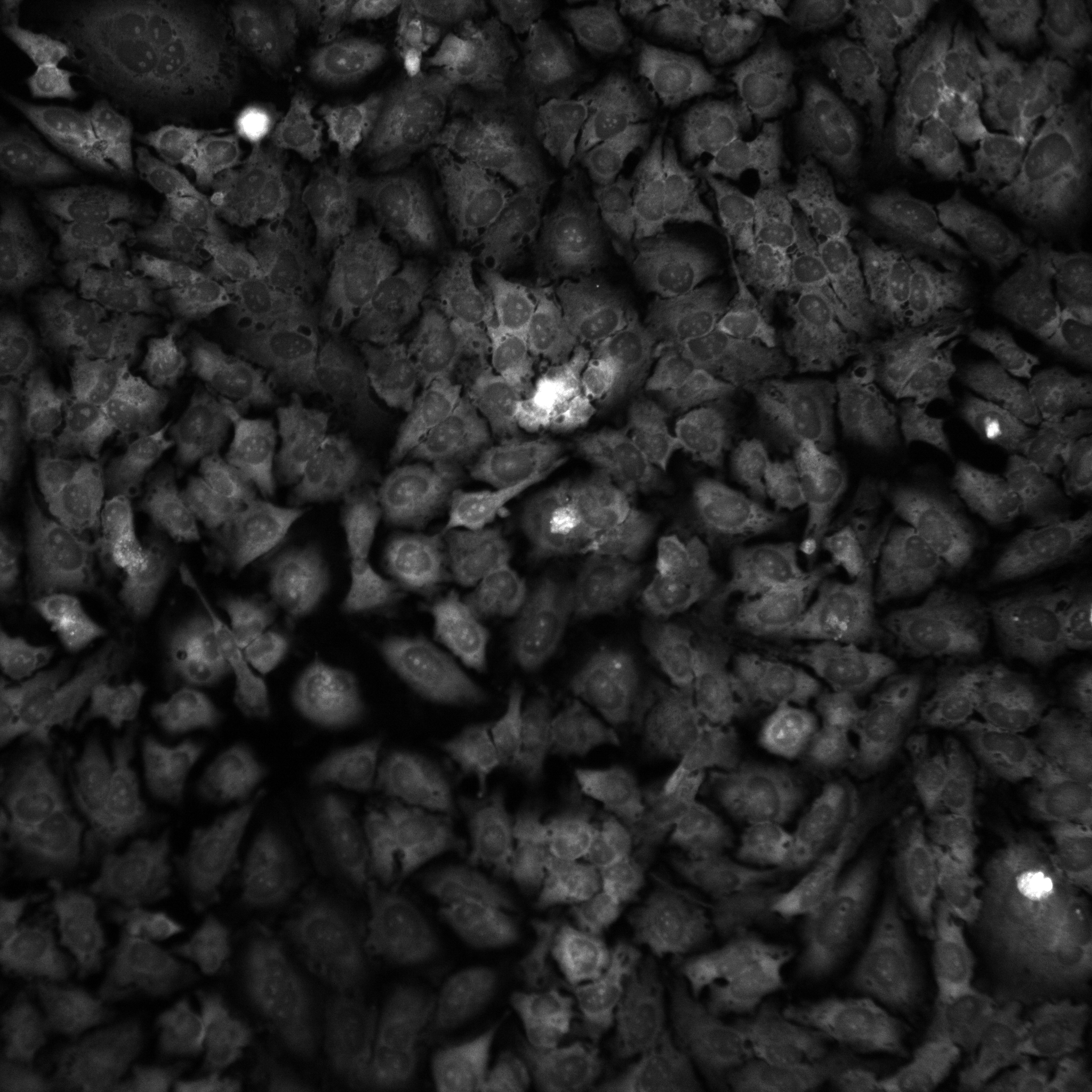

Supplement: Supplementary file 1 — Sample images and results. Sample datasets used in this paper (# 1 and #5 in table 2). The dataset includes input images of both dsRed and Cy5 channels and the corresponding cell segmentation. (ZIP 245,472 kb) [file 12859_2018_2375_MOESM1_ESM.zip › FYVE Hela 1/B - 3(fld 1 wv Green - dsRed).tif]

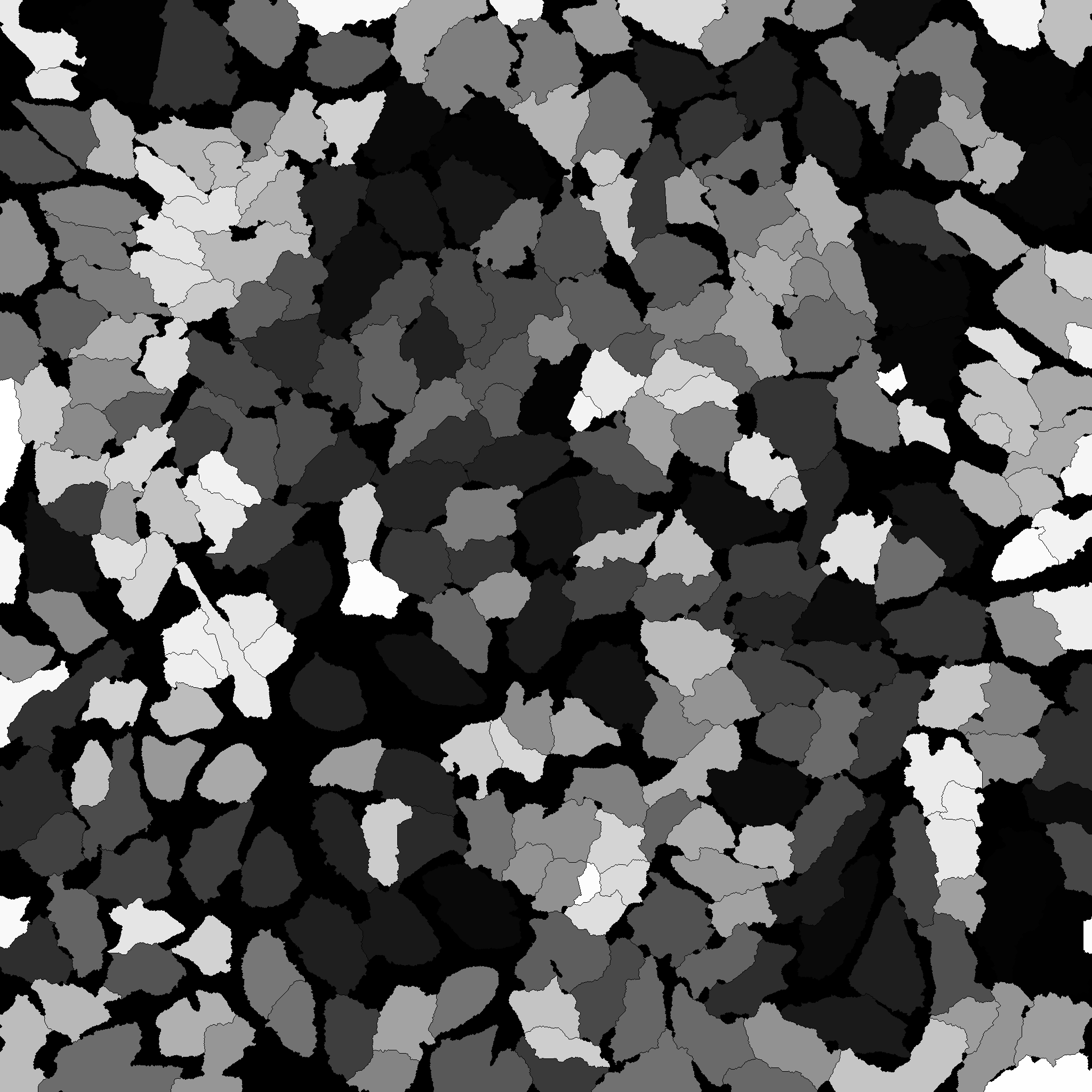

Supplement: Supplementary file 1 — Sample images and results. Sample datasets used in this paper (# 1 and #5 in table 2). The dataset includes input images of both dsRed and Cy5 channels and the corresponding cell segmentation. (ZIP 245,472 kb) [file 12859_2018_2375_MOESM1_ESM.zip › FYVE Hela 1/B - 3(fld 1 wv Green - dsRed)_cellseg_label.tif]

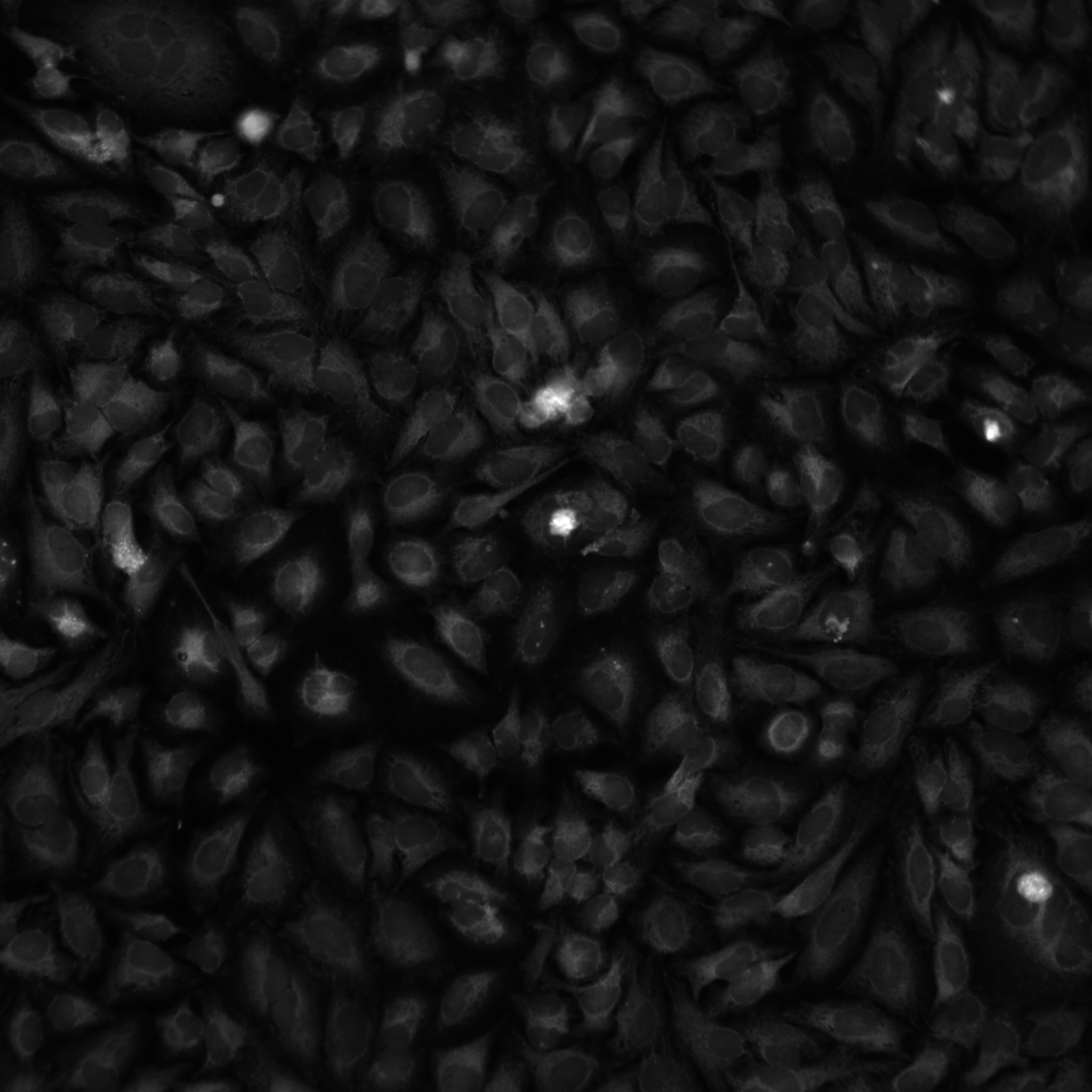

Supplement: Supplementary file 1 — Sample images and results. Sample datasets used in this paper (# 1 and #5 in table 2). The dataset includes input images of both dsRed and Cy5 channels and the corresponding cell segmentation. (ZIP 245,472 kb) [file 12859_2018_2375_MOESM1_ESM.zip › FYVE Hela 1/B - 3(fld 1 wv Red - Cy5).tif]

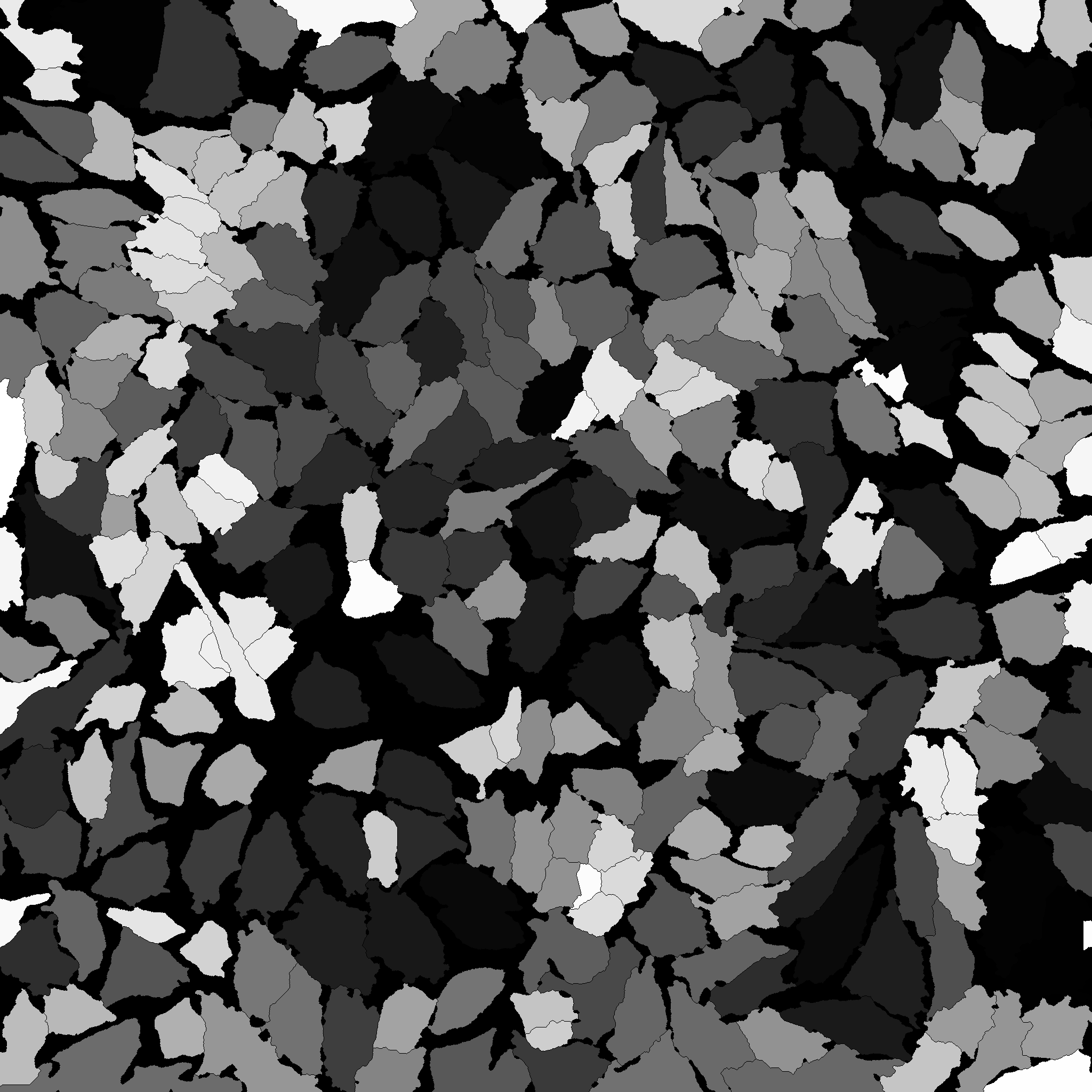

Supplement: Supplementary file 1 — Sample images and results. Sample datasets used in this paper (# 1 and #5 in table 2). The dataset includes input images of both dsRed and Cy5 channels and the corresponding cell segmentation. (ZIP 245,472 kb) [file 12859_2018_2375_MOESM1_ESM.zip › FYVE Hela 1/B - 3(fld 1 wv Red - Cy5)_cellseg_label.tif]

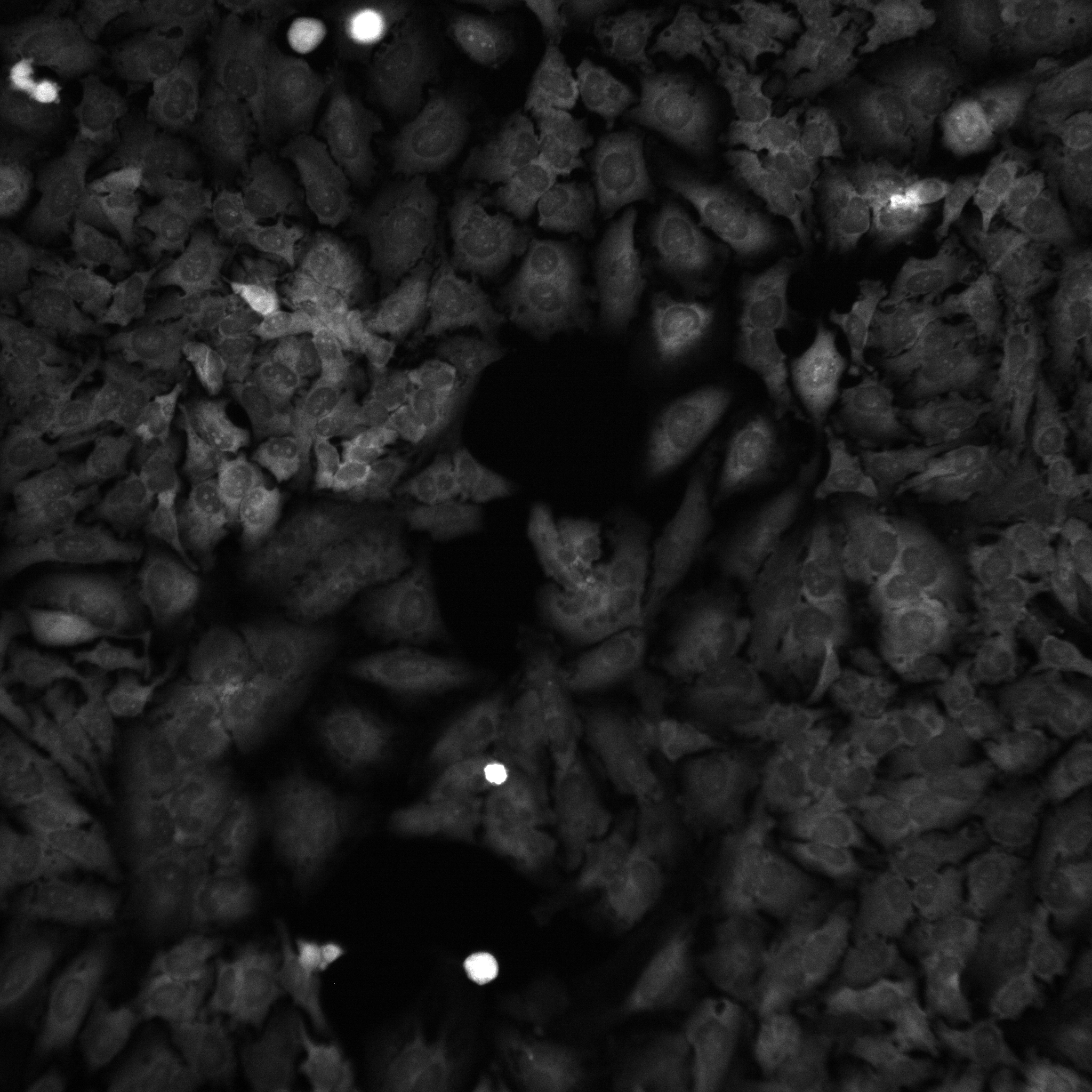

Supplement: Supplementary file 1 — Sample images and results. Sample datasets used in this paper (# 1 and #5 in table 2). The dataset includes input images of both dsRed and Cy5 channels and the corresponding cell segmentation. (ZIP 245,472 kb) [file 12859_2018_2375_MOESM1_ESM.zip › FYVE Hela 1/B - 4(fld 1 wv Green - dsRed).tif]

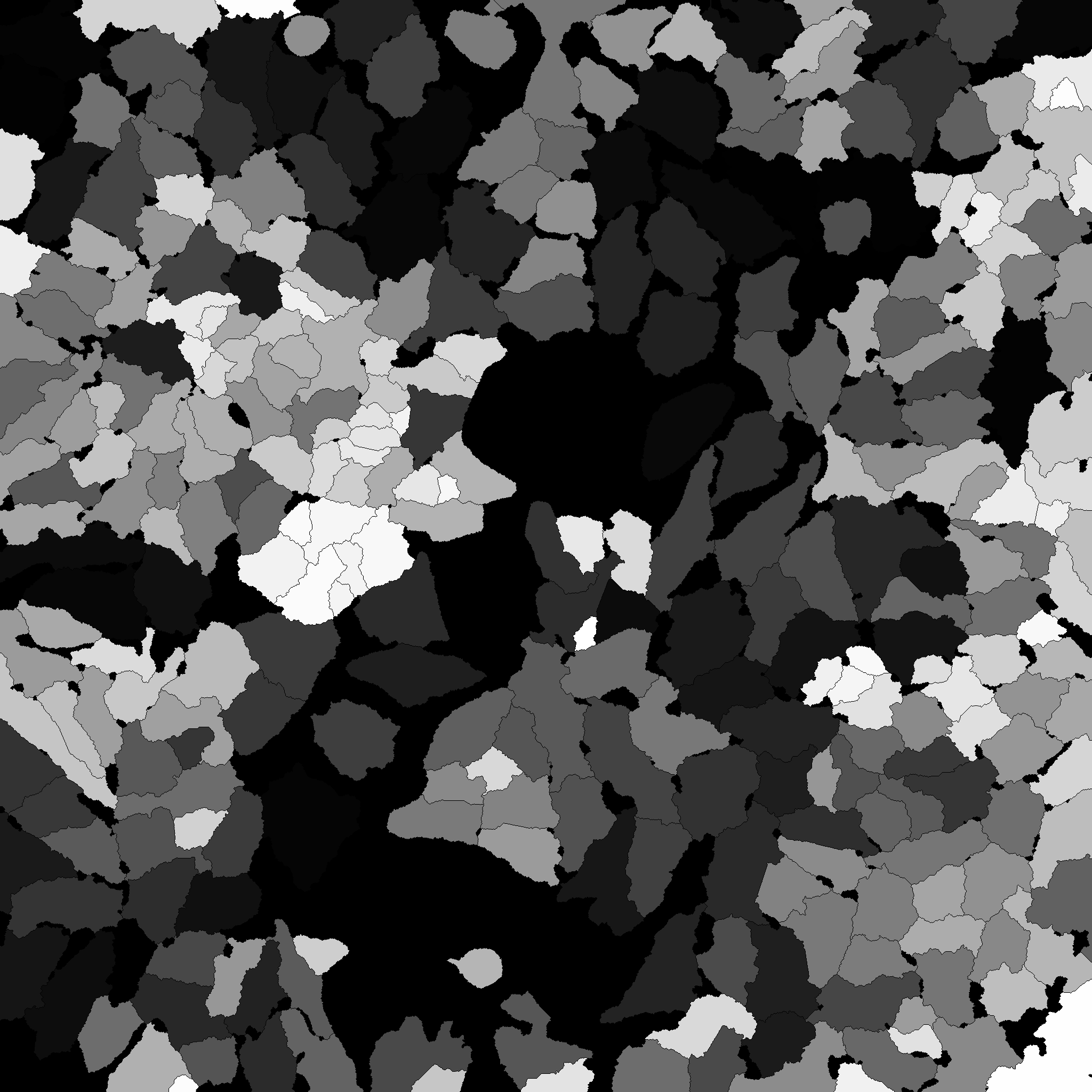

Supplement: Supplementary file 1 — Sample images and results. Sample datasets used in this paper (# 1 and #5 in table 2). The dataset includes input images of both dsRed and Cy5 channels and the corresponding cell segmentation. (ZIP 245,472 kb) [file 12859_2018_2375_MOESM1_ESM.zip › FYVE Hela 1/B - 4(fld 1 wv Green - dsRed)_cellseg_label.tif]

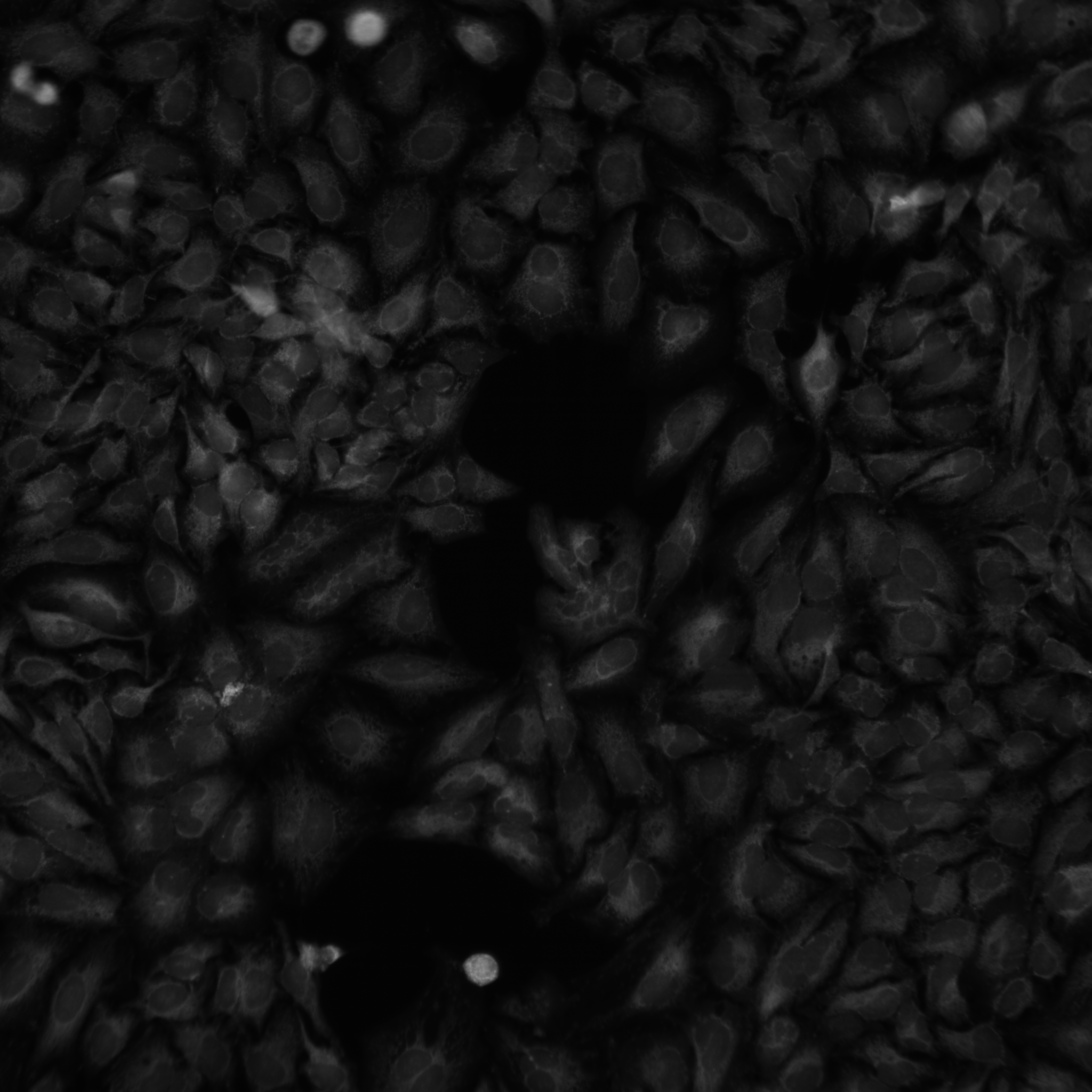

Supplement: Supplementary file 1 — Sample images and results. Sample datasets used in this paper (# 1 and #5 in table 2). The dataset includes input images of both dsRed and Cy5 channels and the corresponding cell segmentation. (ZIP 245,472 kb) [file 12859_2018_2375_MOESM1_ESM.zip › FYVE Hela 1/B - 4(fld 1 wv Red - Cy5).tif]

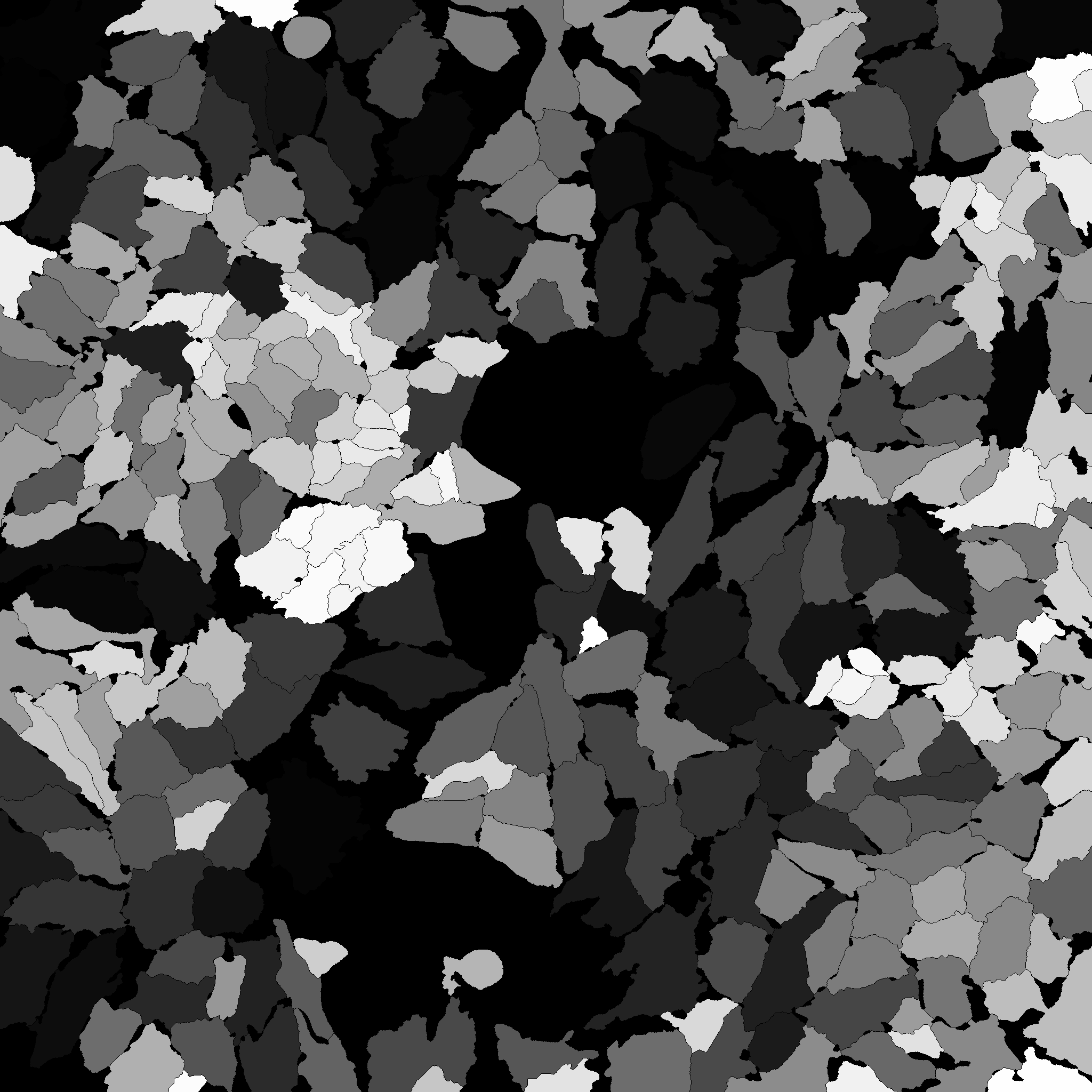

Supplement: Supplementary file 1 — Sample images and results. Sample datasets used in this paper (# 1 and #5 in table 2). The dataset includes input images of both dsRed and Cy5 channels and the corresponding cell segmentation. (ZIP 245,472 kb) [file 12859_2018_2375_MOESM1_ESM.zip › FYVE Hela 1/B - 4(fld 1 wv Red - Cy5)_cellseg_label.tif]

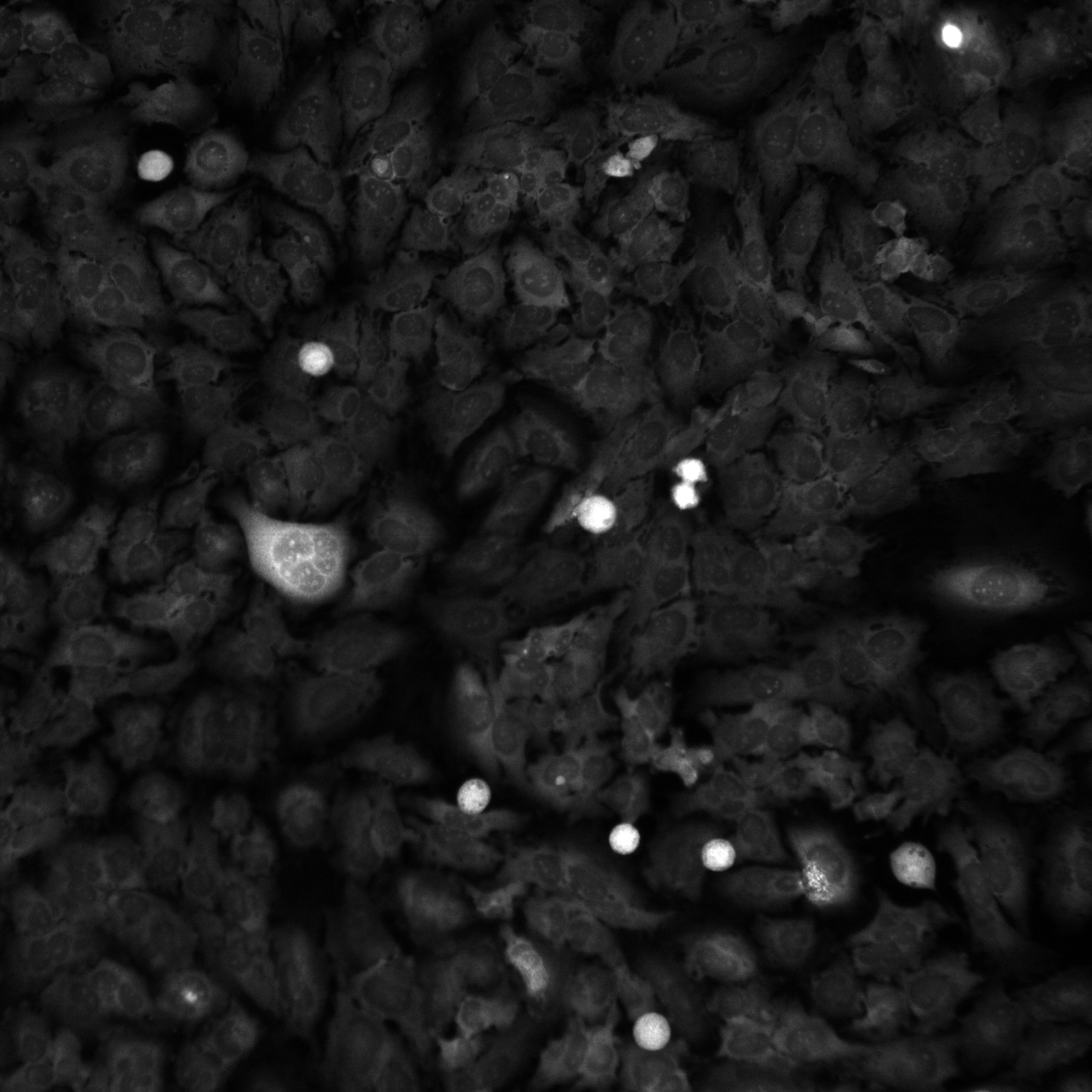

Supplement: Supplementary file 1 — Sample images and results. Sample datasets used in this paper (# 1 and #5 in table 2). The dataset includes input images of both dsRed and Cy5 channels and the corresponding cell segmentation. (ZIP 245,472 kb) [file 12859_2018_2375_MOESM1_ESM.zip › FYVE Hela 1/B - 5(fld 1 wv Green - dsRed).tif]

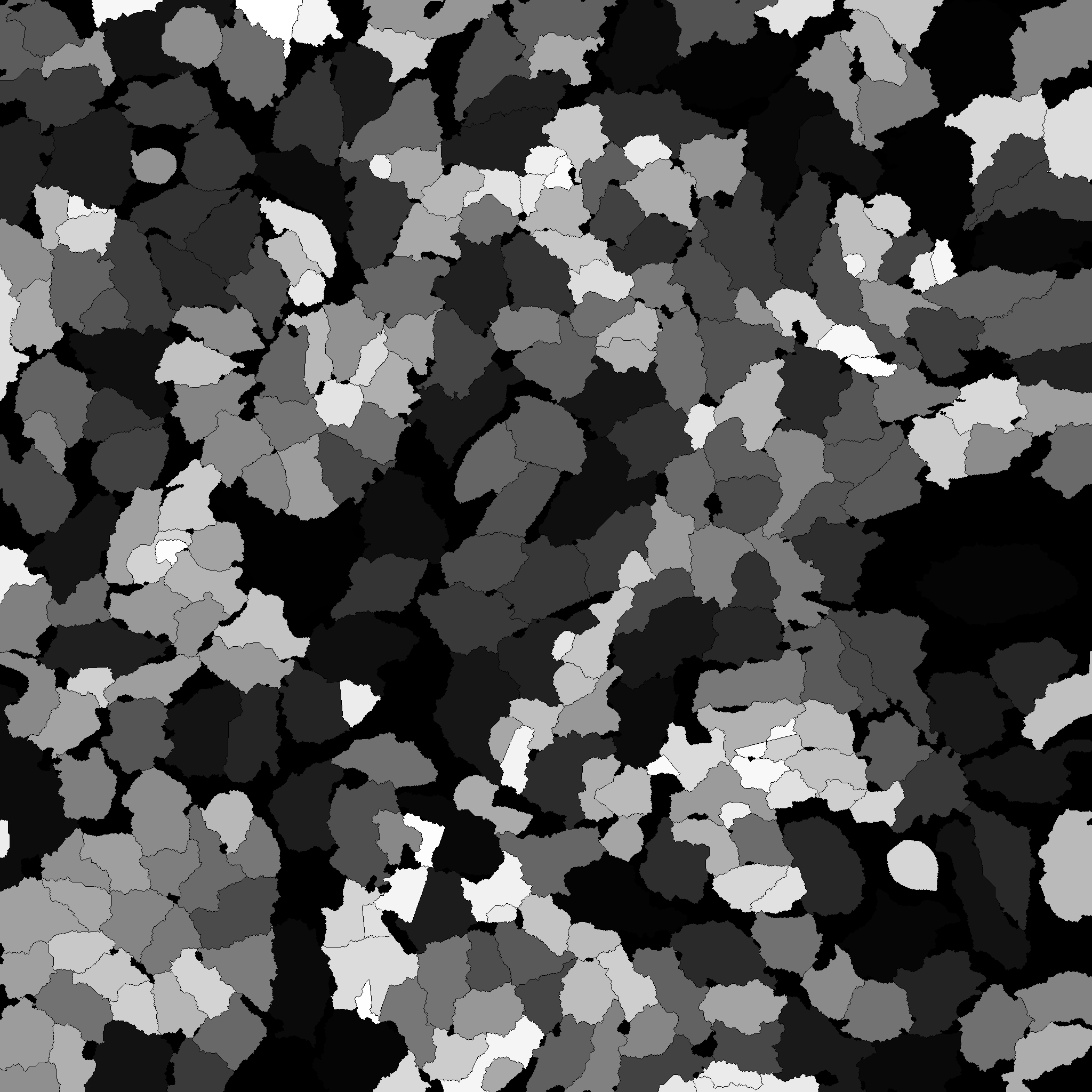

Supplement: Supplementary file 1 — Sample images and results. Sample datasets used in this paper (# 1 and #5 in table 2). The dataset includes input images of both dsRed and Cy5 channels and the corresponding cell segmentation. (ZIP 245,472 kb) [file 12859_2018_2375_MOESM1_ESM.zip › FYVE Hela 1/B - 5(fld 1 wv Green - dsRed)_cellseg_label.tif]

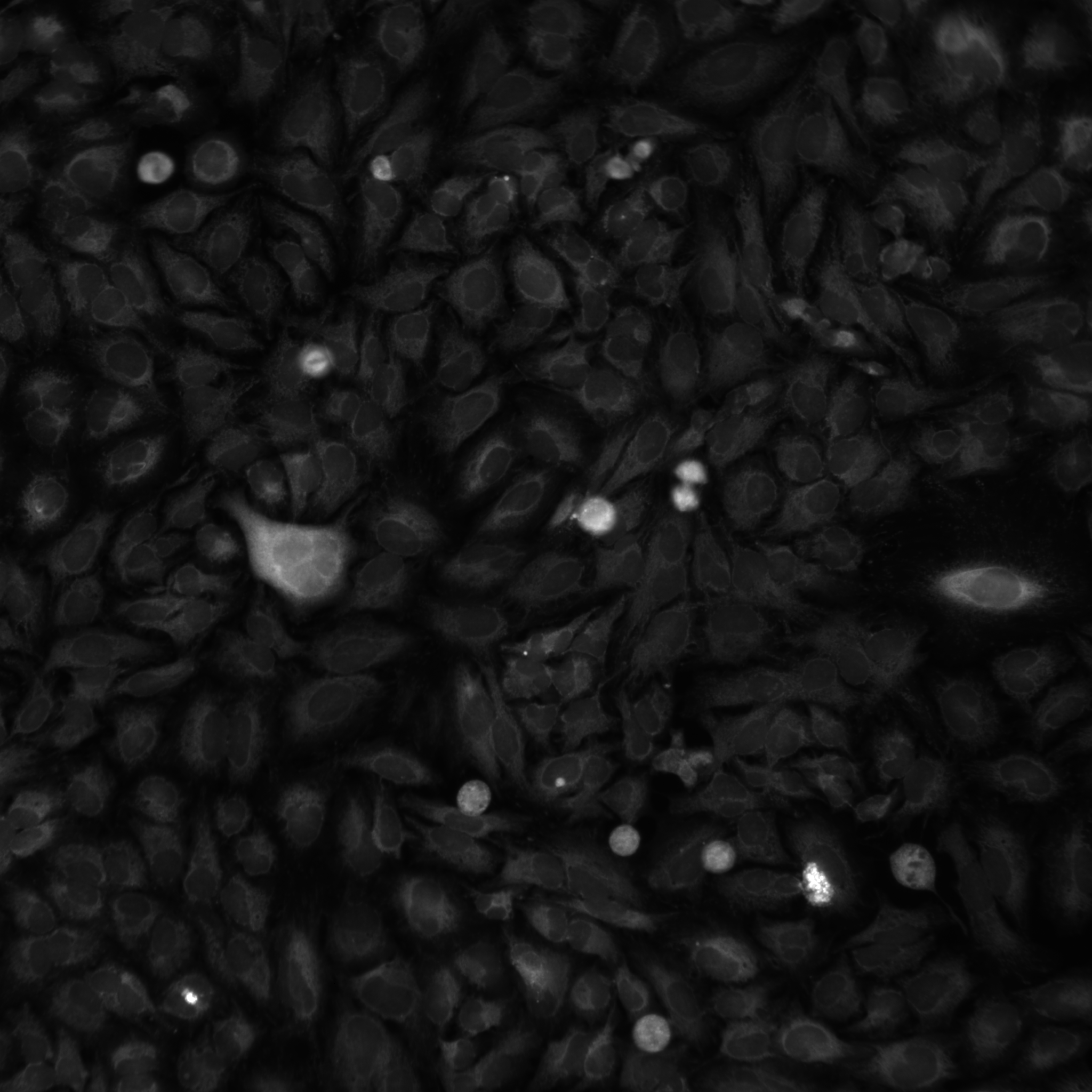

Supplement: Supplementary file 1 — Sample images and results. Sample datasets used in this paper (# 1 and #5 in table 2). The dataset includes input images of both dsRed and Cy5 channels and the corresponding cell segmentation. (ZIP 245,472 kb) [file 12859_2018_2375_MOESM1_ESM.zip › FYVE Hela 1/B - 5(fld 1 wv Red - Cy5).tif]

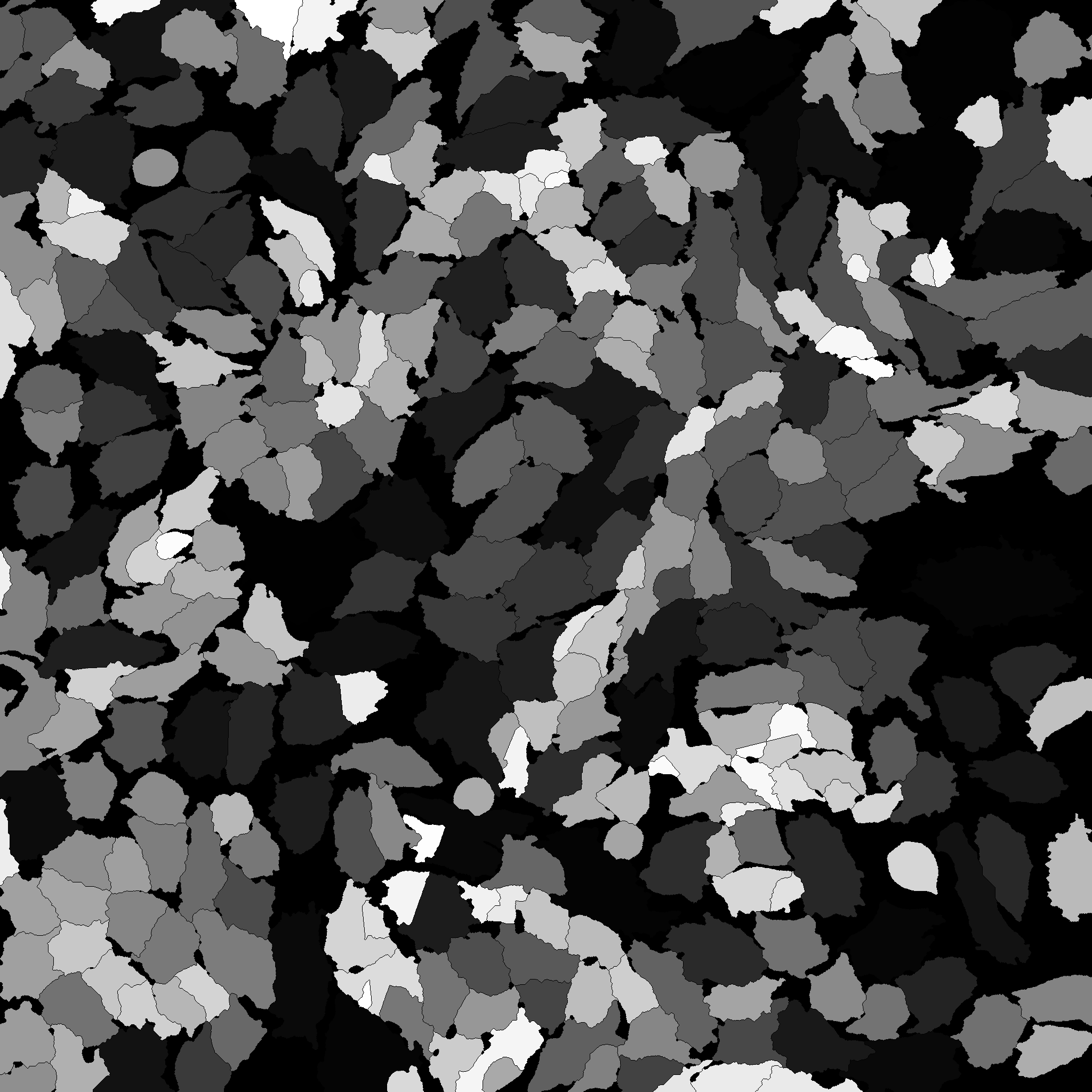

Supplement: Supplementary file 1 — Sample images and results. Sample datasets used in this paper (# 1 and #5 in table 2). The dataset includes input images of both dsRed and Cy5 channels and the corresponding cell segmentation. (ZIP 245,472 kb) [file 12859_2018_2375_MOESM1_ESM.zip › FYVE Hela 1/B - 5(fld 1 wv Red - Cy5)_cellseg_label.tif]

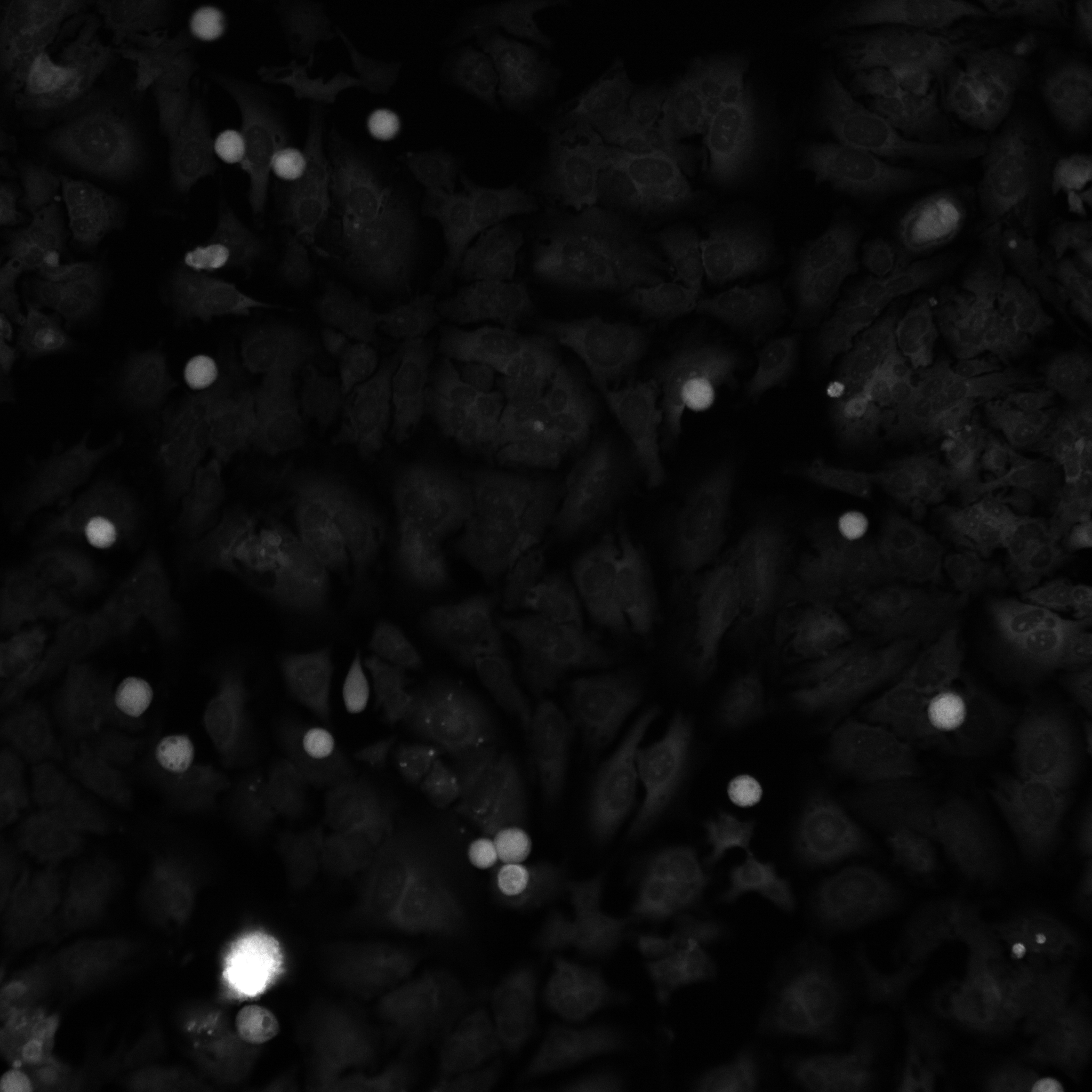

Supplement: Supplementary file 1 — Sample images and results. Sample datasets used in this paper (# 1 and #5 in table 2). The dataset includes input images of both dsRed and Cy5 channels and the corresponding cell segmentation. (ZIP 245,472 kb) [file 12859_2018_2375_MOESM1_ESM.zip › FYVE Hela 1/B - 6(fld 1 wv Green - dsRed).tif]

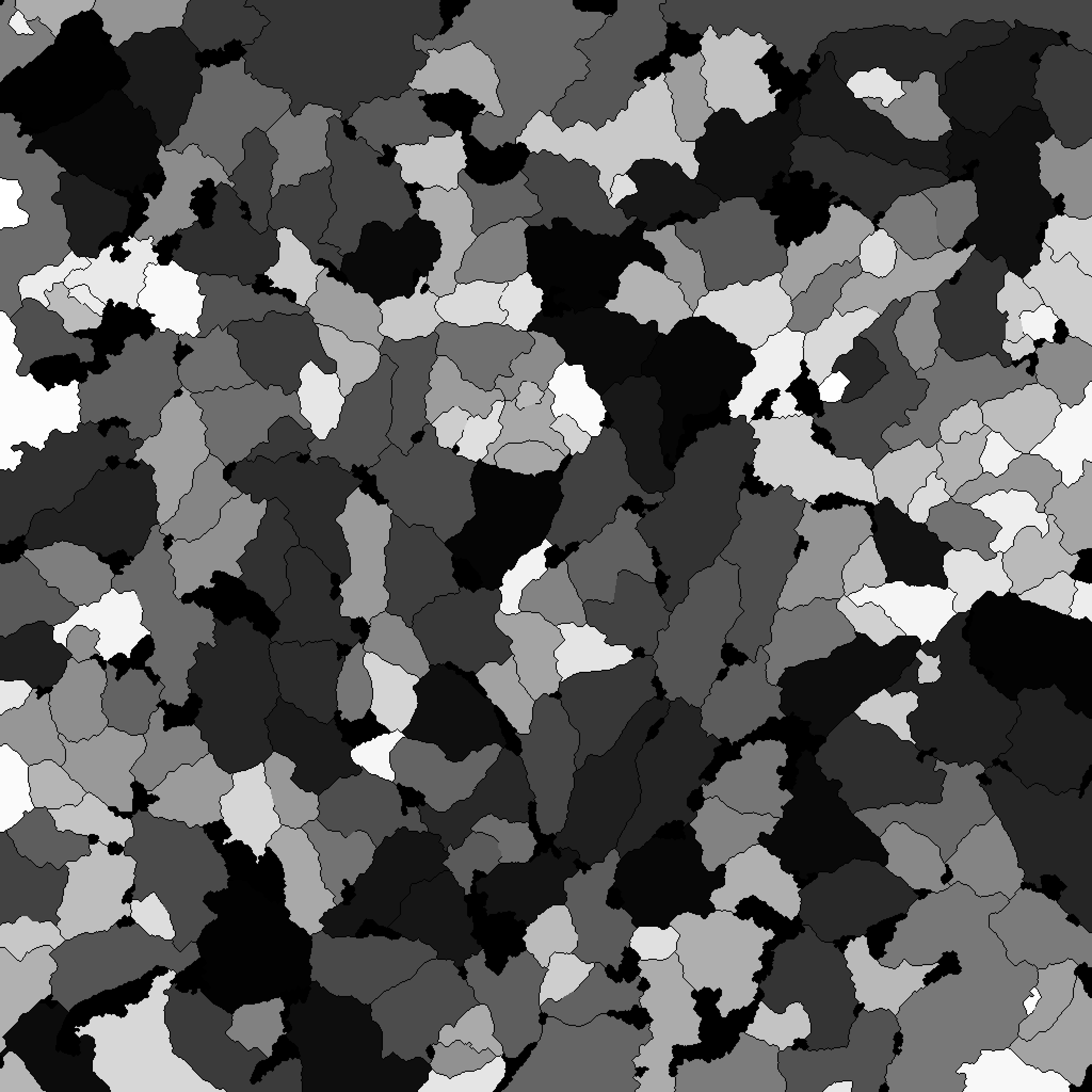

Supplement: Supplementary file 1 — Sample images and results. Sample datasets used in this paper (# 1 and #5 in table 2). The dataset includes input images of both dsRed and Cy5 channels and the corresponding cell segmentation. (ZIP 245,472 kb) [file 12859_2018_2375_MOESM1_ESM.zip › FYVE Hela 1/B - 6(fld 1 wv Green - dsRed)_cellseg_label.tif]

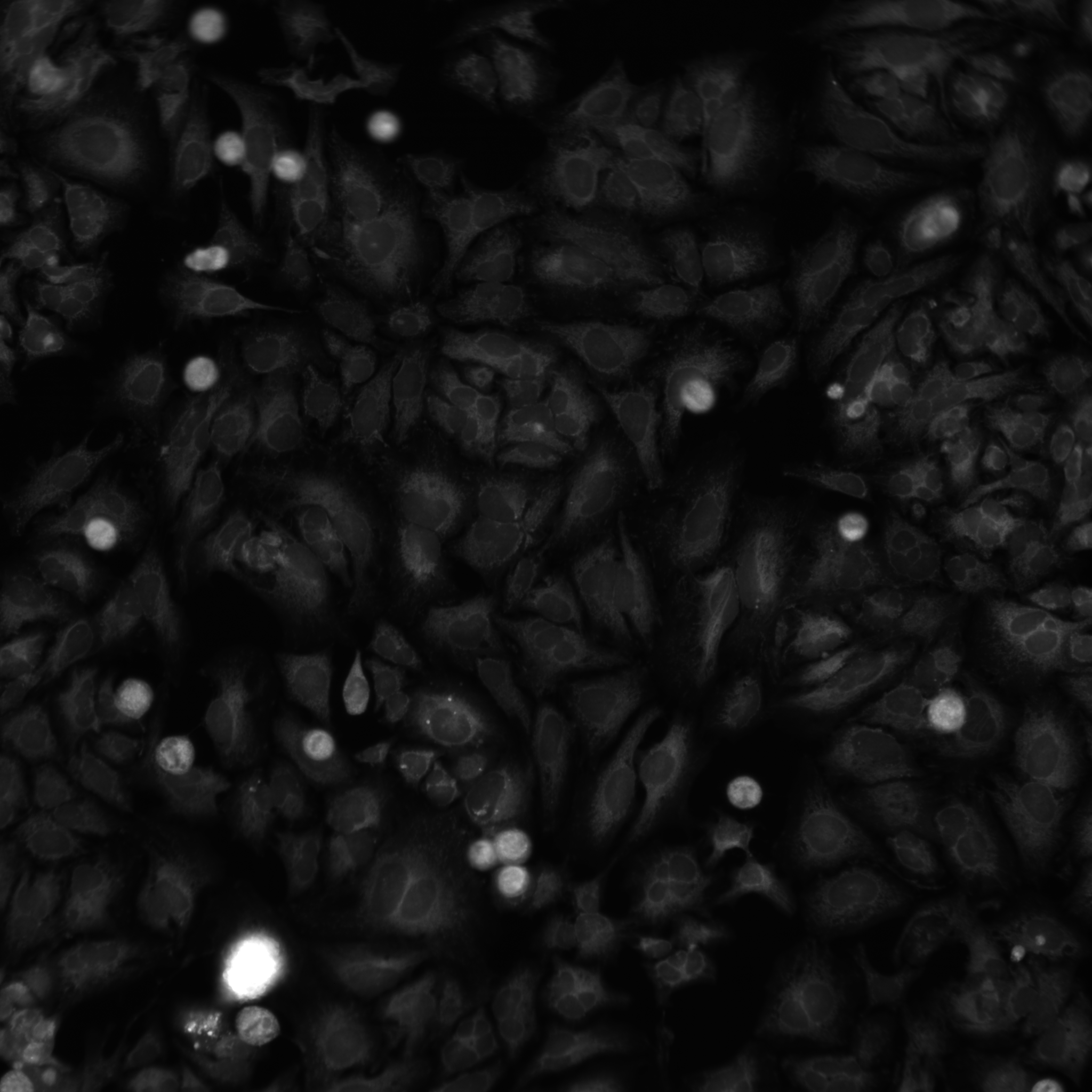

Supplement: Supplementary file 1 — Sample images and results. Sample datasets used in this paper (# 1 and #5 in table 2). The dataset includes input images of both dsRed and Cy5 channels and the corresponding cell segmentation. (ZIP 245,472 kb) [file 12859_2018_2375_MOESM1_ESM.zip › FYVE Hela 1/B - 6(fld 1 wv Red - Cy5).tif]

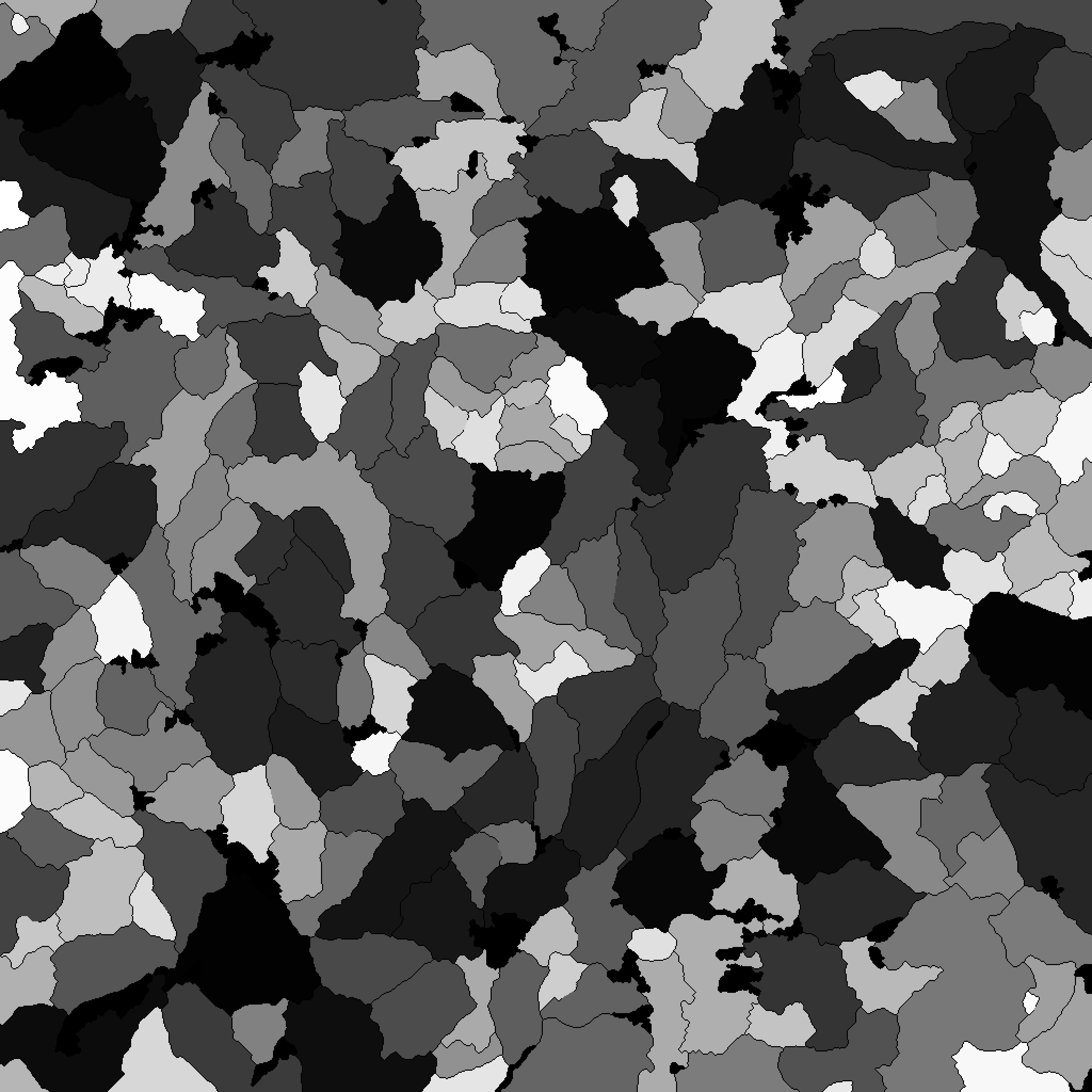

Supplement: Supplementary file 1 — Sample images and results. Sample datasets used in this paper (# 1 and #5 in table 2). The dataset includes input images of both dsRed and Cy5 channels and the corresponding cell segmentation. (ZIP 245,472 kb) [file 12859_2018_2375_MOESM1_ESM.zip › FYVE Hela 1/B - 6(fld 1 wv Red - Cy5)_cellseg_label.tif]

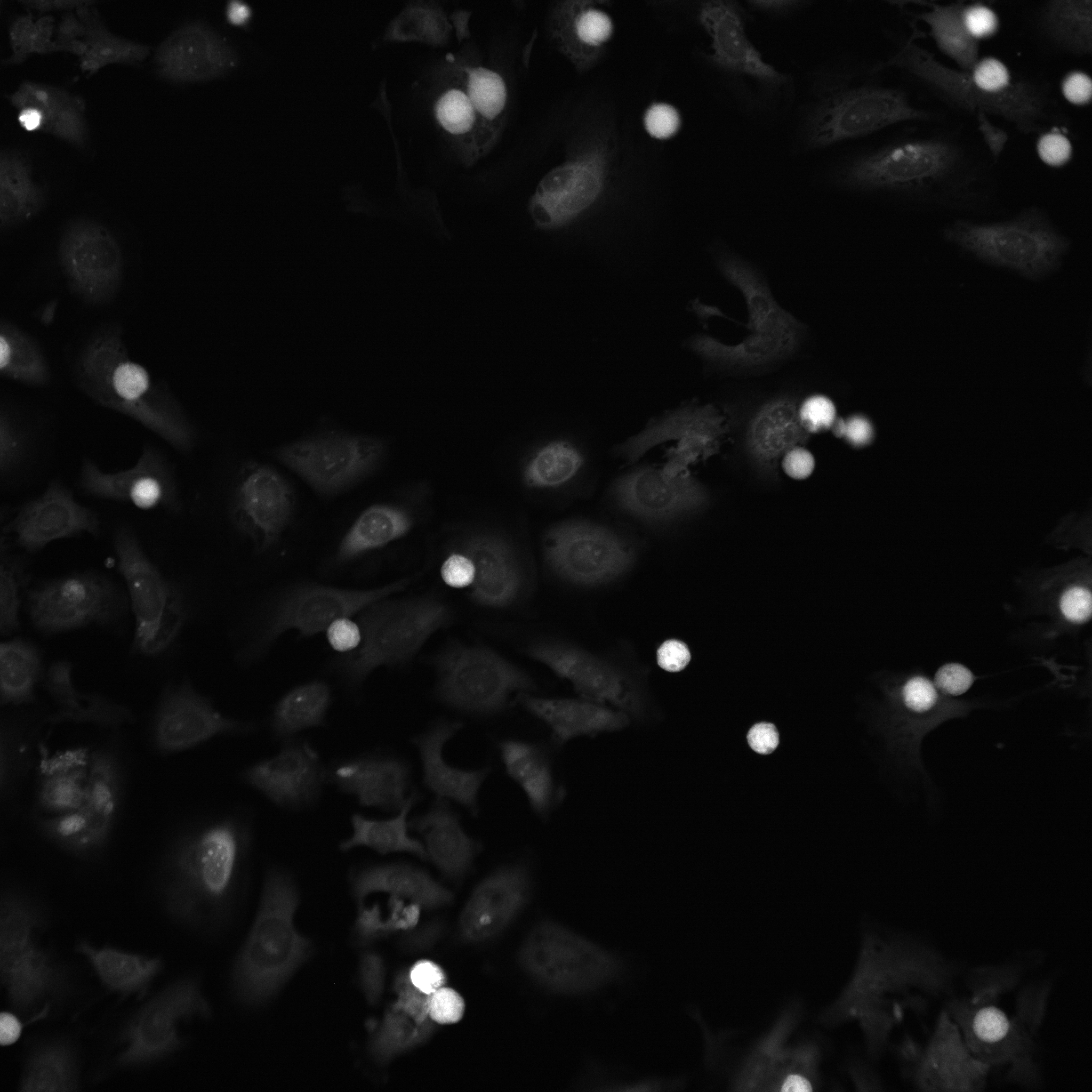

Supplement: Supplementary file 1 — Sample images and results. Sample datasets used in this paper (# 1 and #5 in table 2). The dataset includes input images of both dsRed and Cy5 channels and the corresponding cell segmentation. (ZIP 245,472 kb) [file 12859_2018_2375_MOESM1_ESM.zip › FYVE Hela 1/B - 7(fld 1 wv Green - dsRed).tif]

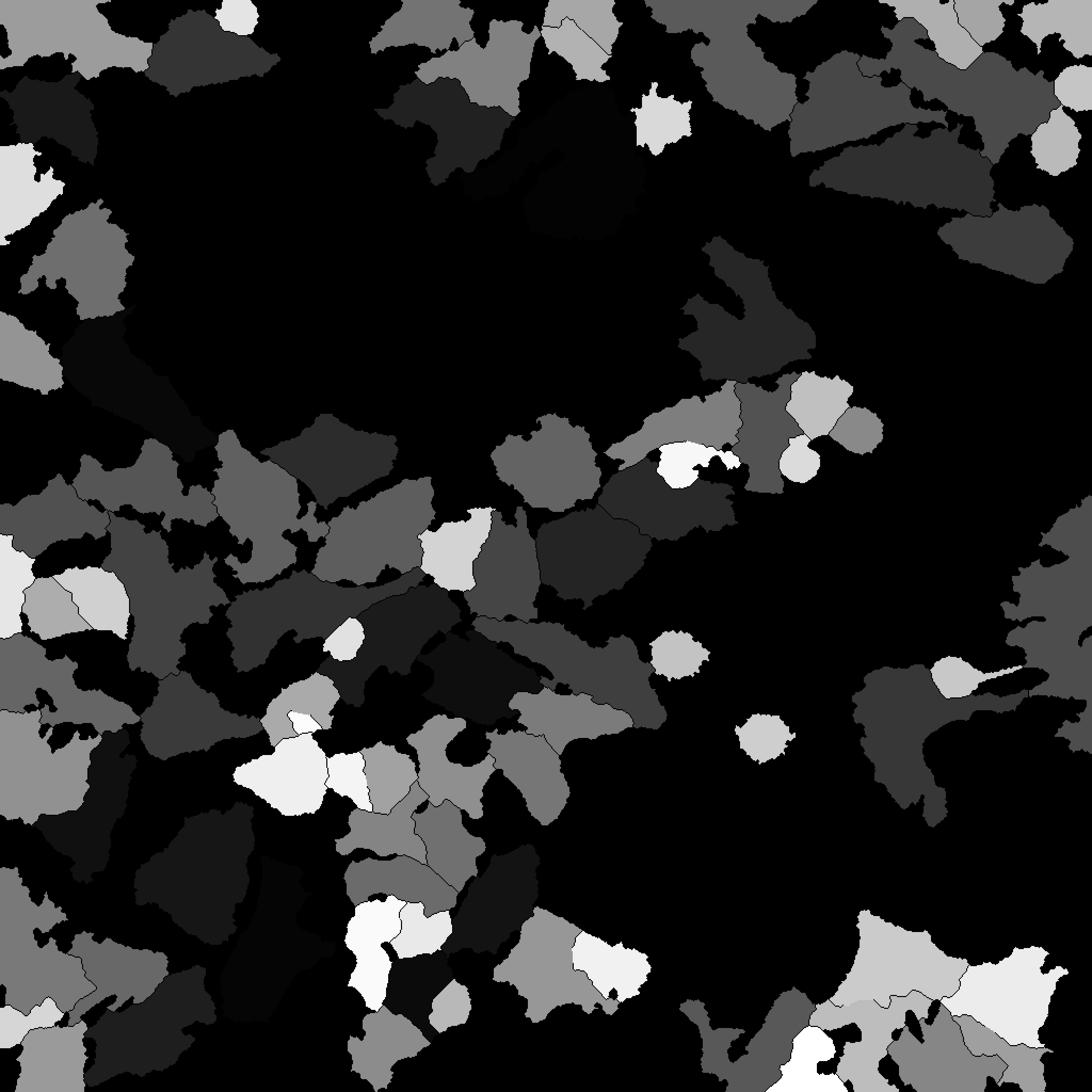

Supplement: Supplementary file 1 — Sample images and results. Sample datasets used in this paper (# 1 and #5 in table 2). The dataset includes input images of both dsRed and Cy5 channels and the corresponding cell segmentation. (ZIP 245,472 kb) [file 12859_2018_2375_MOESM1_ESM.zip › FYVE Hela 1/B - 7(fld 1 wv Green - dsRed)_cellseg_label.tif]

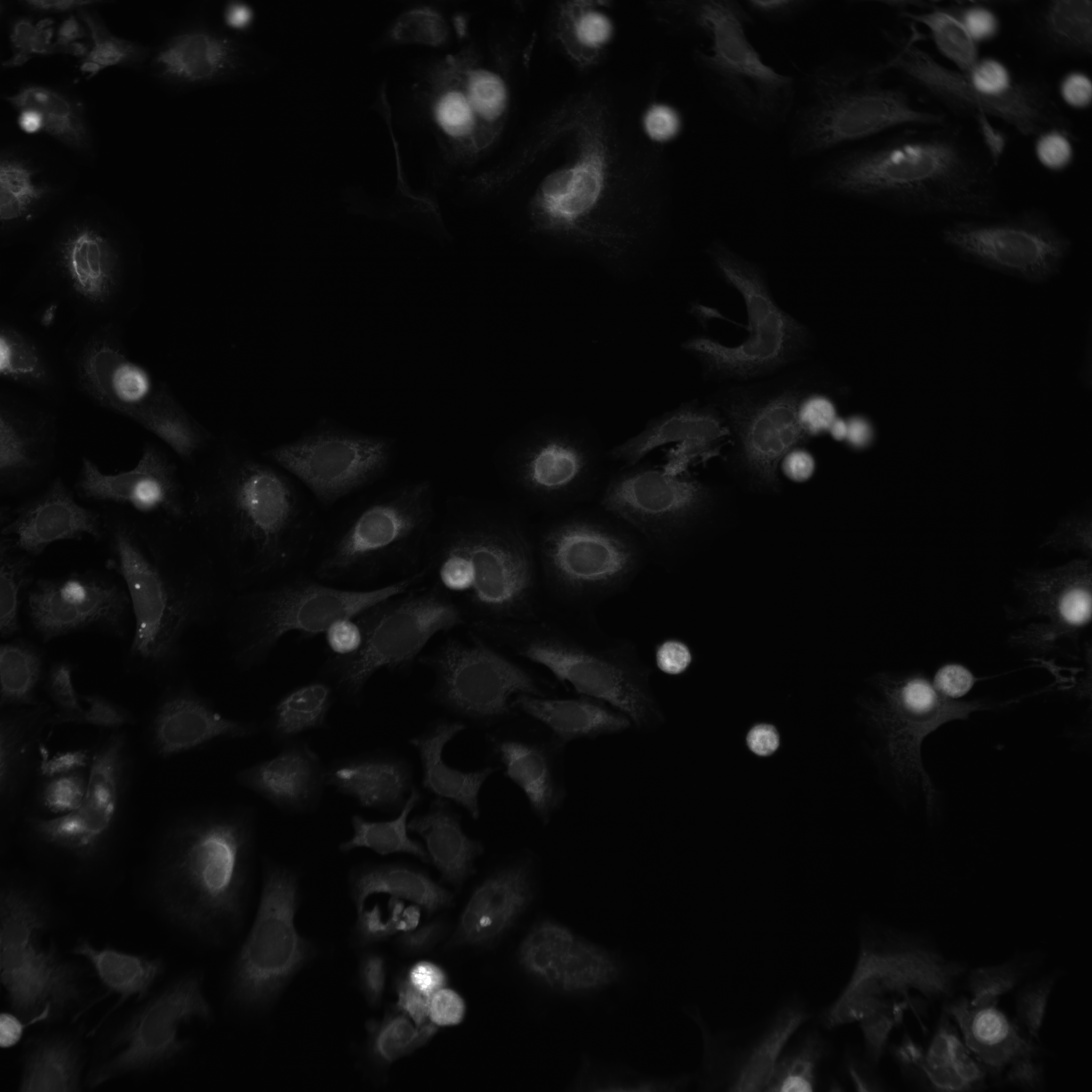

Supplement: Supplementary file 1 — Sample images and results. Sample datasets used in this paper (# 1 and #5 in table 2). The dataset includes input images of both dsRed and Cy5 channels and the corresponding cell segmentation. (ZIP 245,472 kb) [file 12859_2018_2375_MOESM1_ESM.zip › FYVE Hela 1/B - 7(fld 1 wv Red - Cy5).tif]

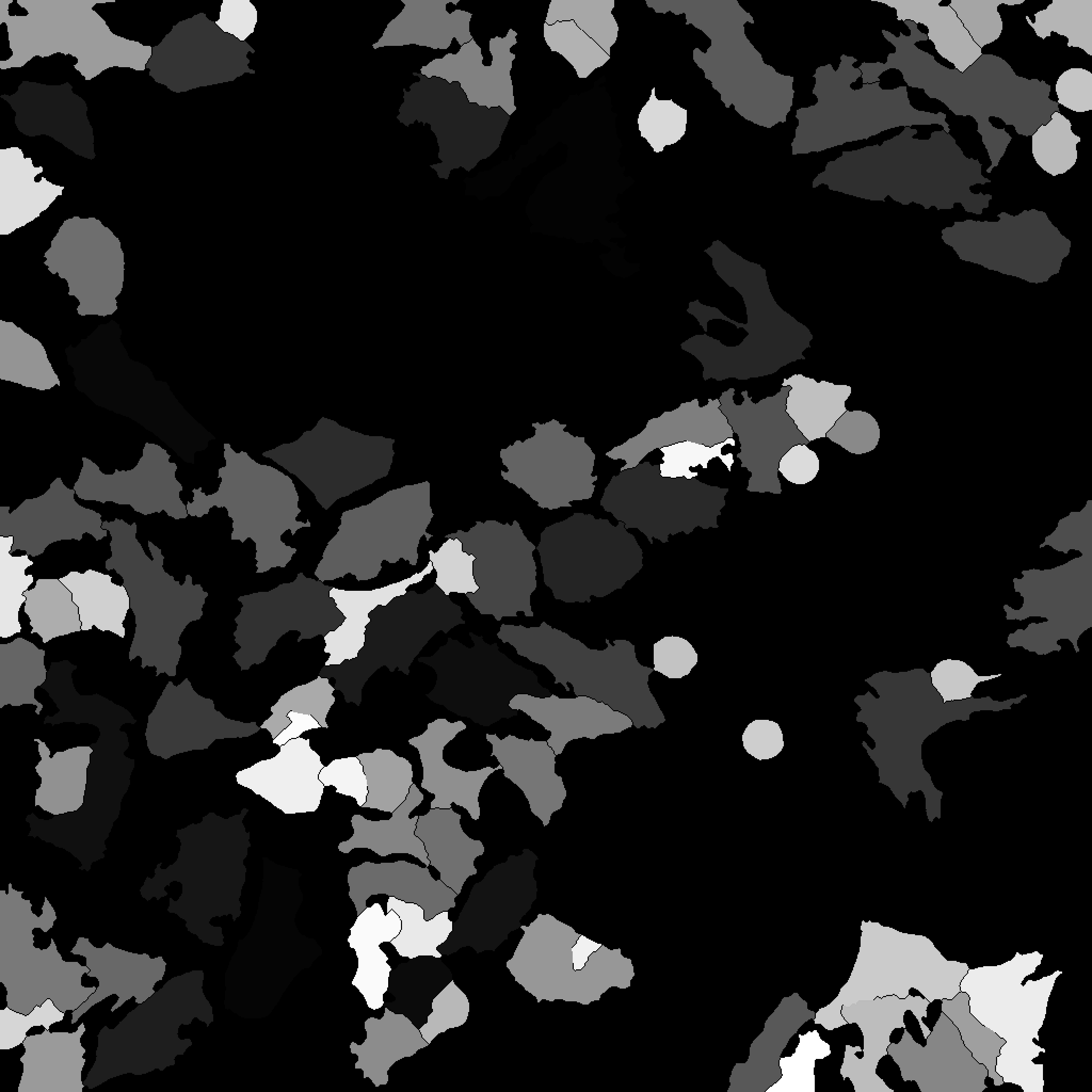

Supplement: Supplementary file 1 — Sample images and results. Sample datasets used in this paper (# 1 and #5 in table 2). The dataset includes input images of both dsRed and Cy5 channels and the corresponding cell segmentation. (ZIP 245,472 kb) [file 12859_2018_2375_MOESM1_ESM.zip › FYVE Hela 1/B - 7(fld 1 wv Red - Cy5)_cellseg_label.tif]

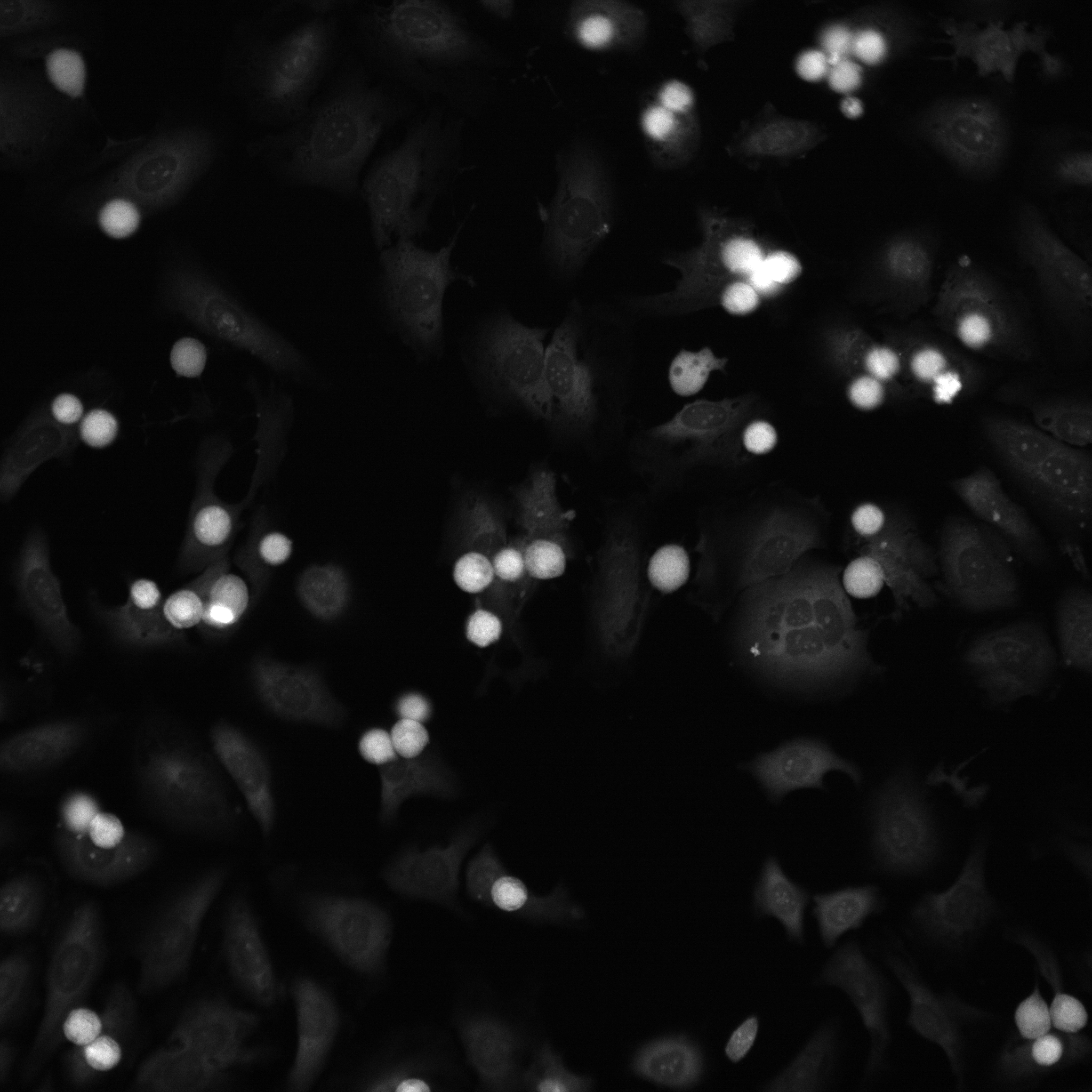

Supplement: Supplementary file 1 — Sample images and results. Sample datasets used in this paper (# 1 and #5 in table 2). The dataset includes input images of both dsRed and Cy5 channels and the corresponding cell segmentation. (ZIP 245,472 kb) [file 12859_2018_2375_MOESM1_ESM.zip › FYVE Hela 1/B - 8(fld 1 wv Green - dsRed).tif]

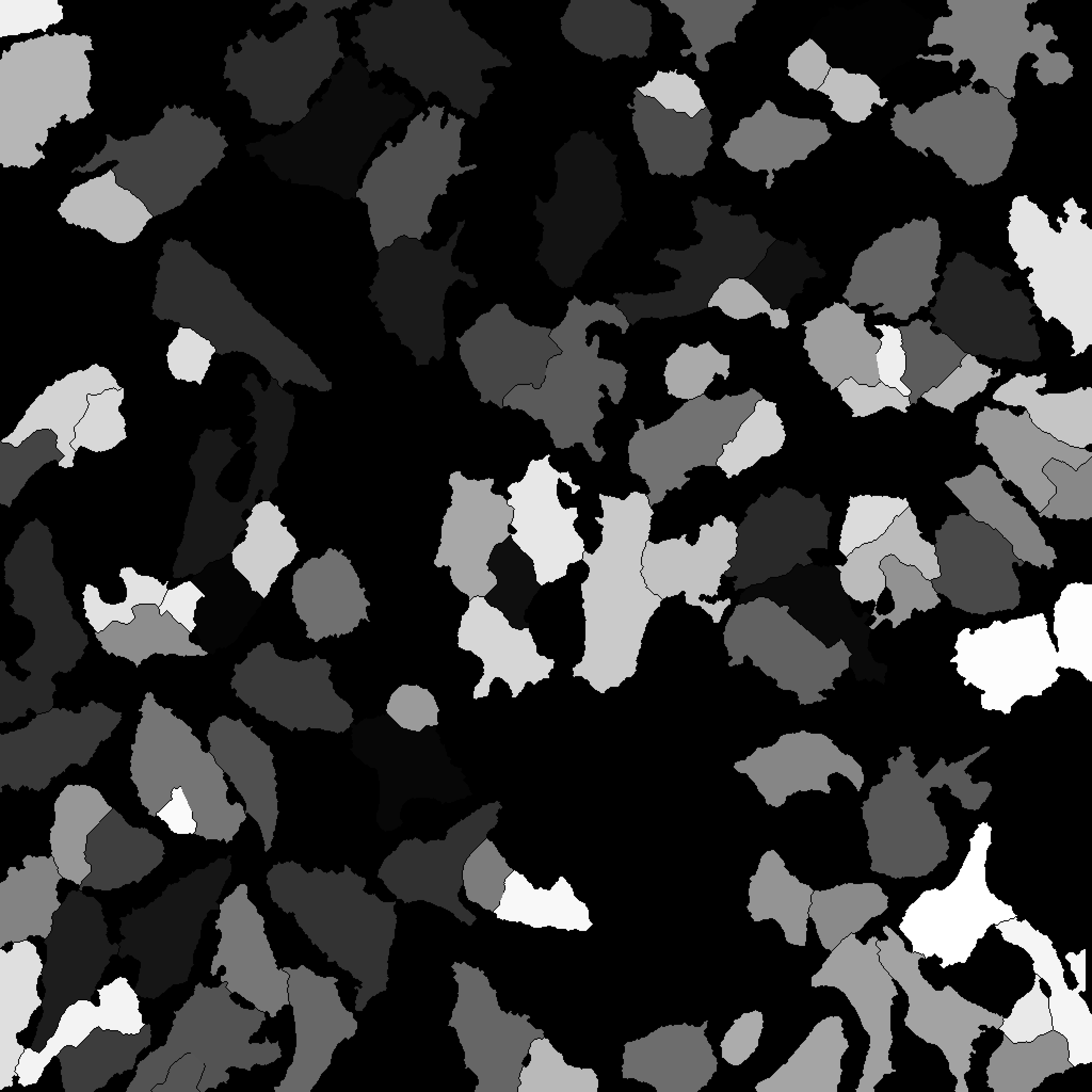

Supplement: Supplementary file 1 — Sample images and results. Sample datasets used in this paper (# 1 and #5 in table 2). The dataset includes input images of both dsRed and Cy5 channels and the corresponding cell segmentation. (ZIP 245,472 kb) [file 12859_2018_2375_MOESM1_ESM.zip › FYVE Hela 1/B - 8(fld 1 wv Green - dsRed)_cellseg_label.tif]

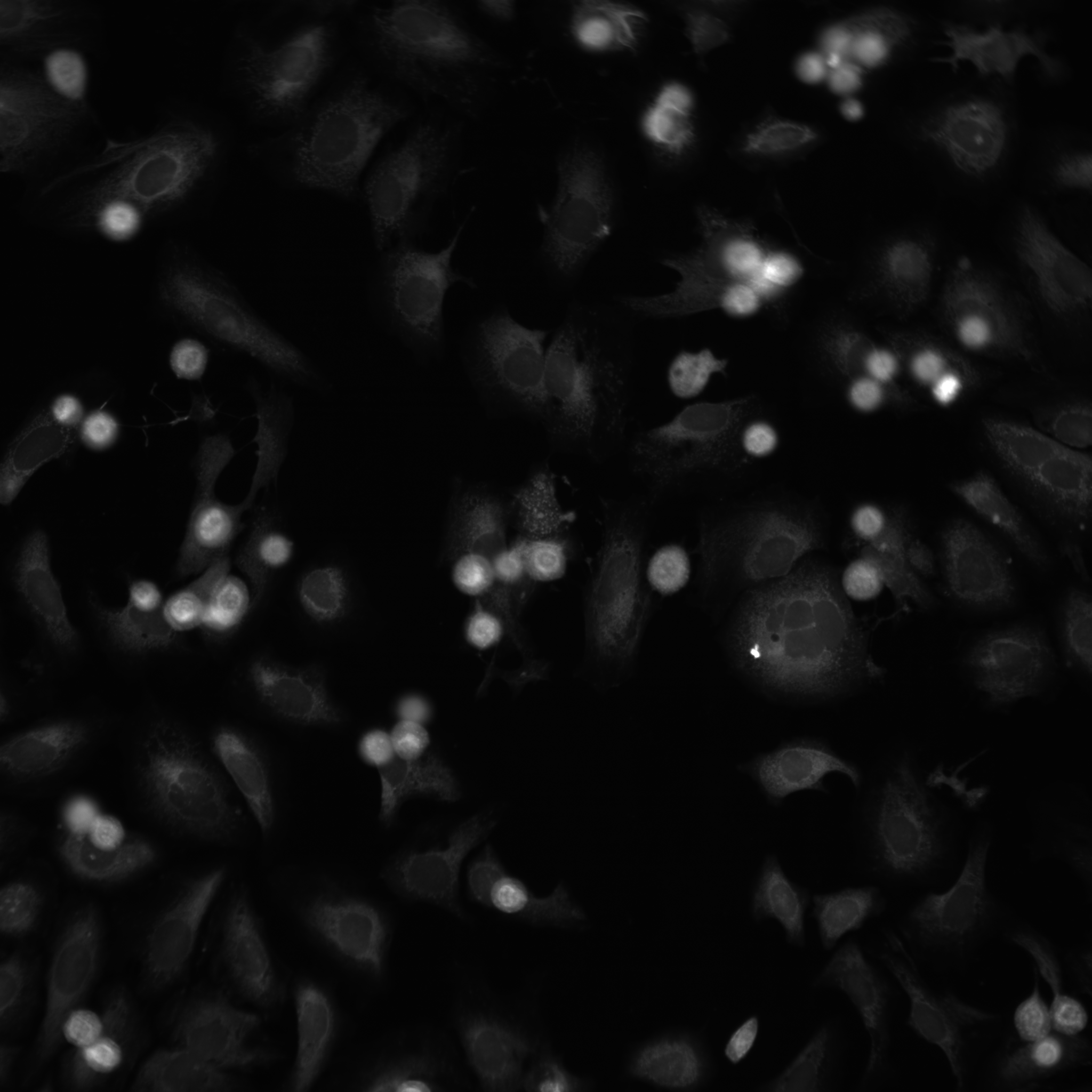

Supplement: Supplementary file 1 — Sample images and results. Sample datasets used in this paper (# 1 and #5 in table 2). The dataset includes input images of both dsRed and Cy5 channels and the corresponding cell segmentation. (ZIP 245,472 kb) [file 12859_2018_2375_MOESM1_ESM.zip › FYVE Hela 1/B - 8(fld 1 wv Red - Cy5).tif]

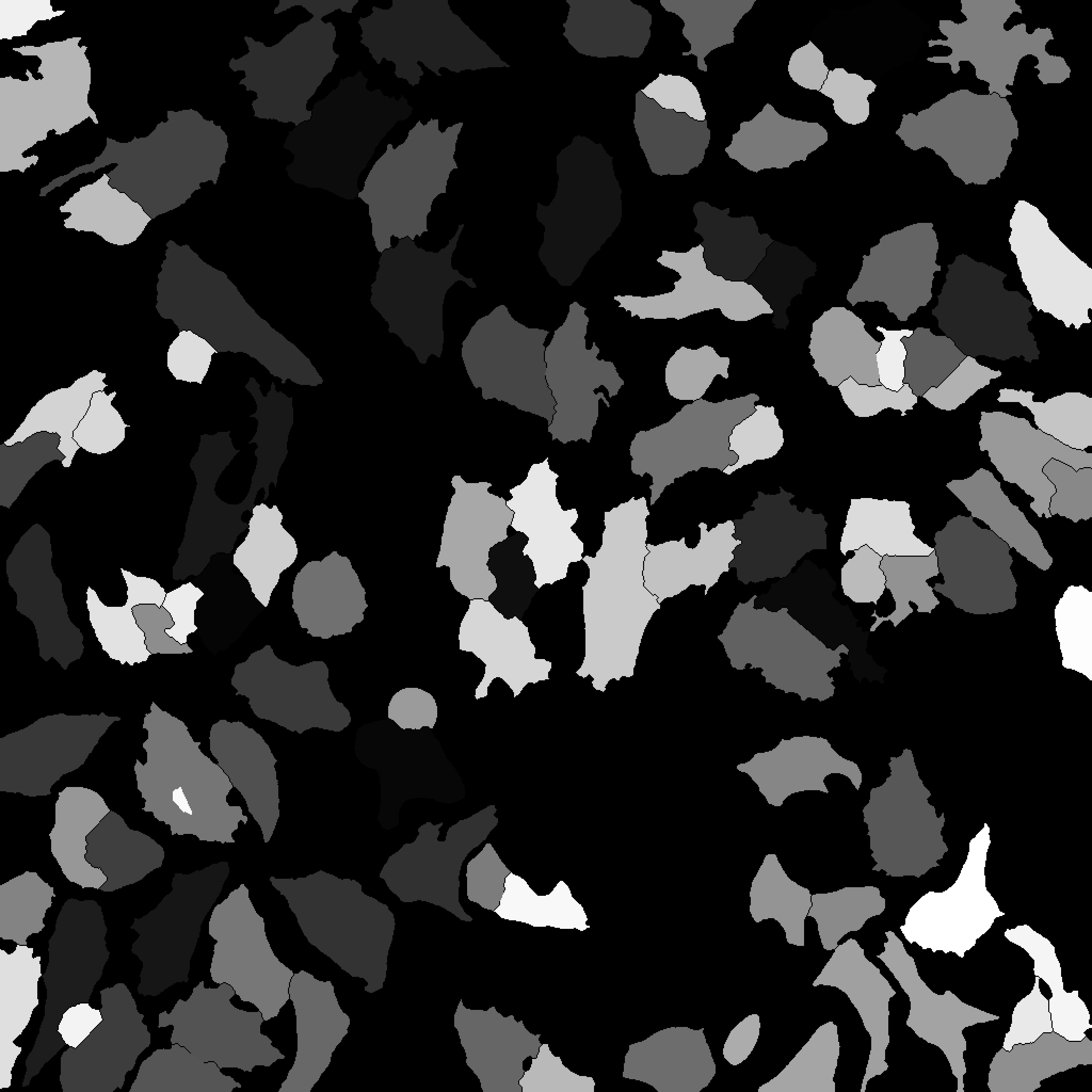

Supplement: Supplementary file 1 — Sample images and results. Sample datasets used in this paper (# 1 and #5 in table 2). The dataset includes input images of both dsRed and Cy5 channels and the corresponding cell segmentation. (ZIP 245,472 kb) [file 12859_2018_2375_MOESM1_ESM.zip › FYVE Hela 1/B - 8(fld 1 wv Red - Cy5)_cellseg_label.tif]

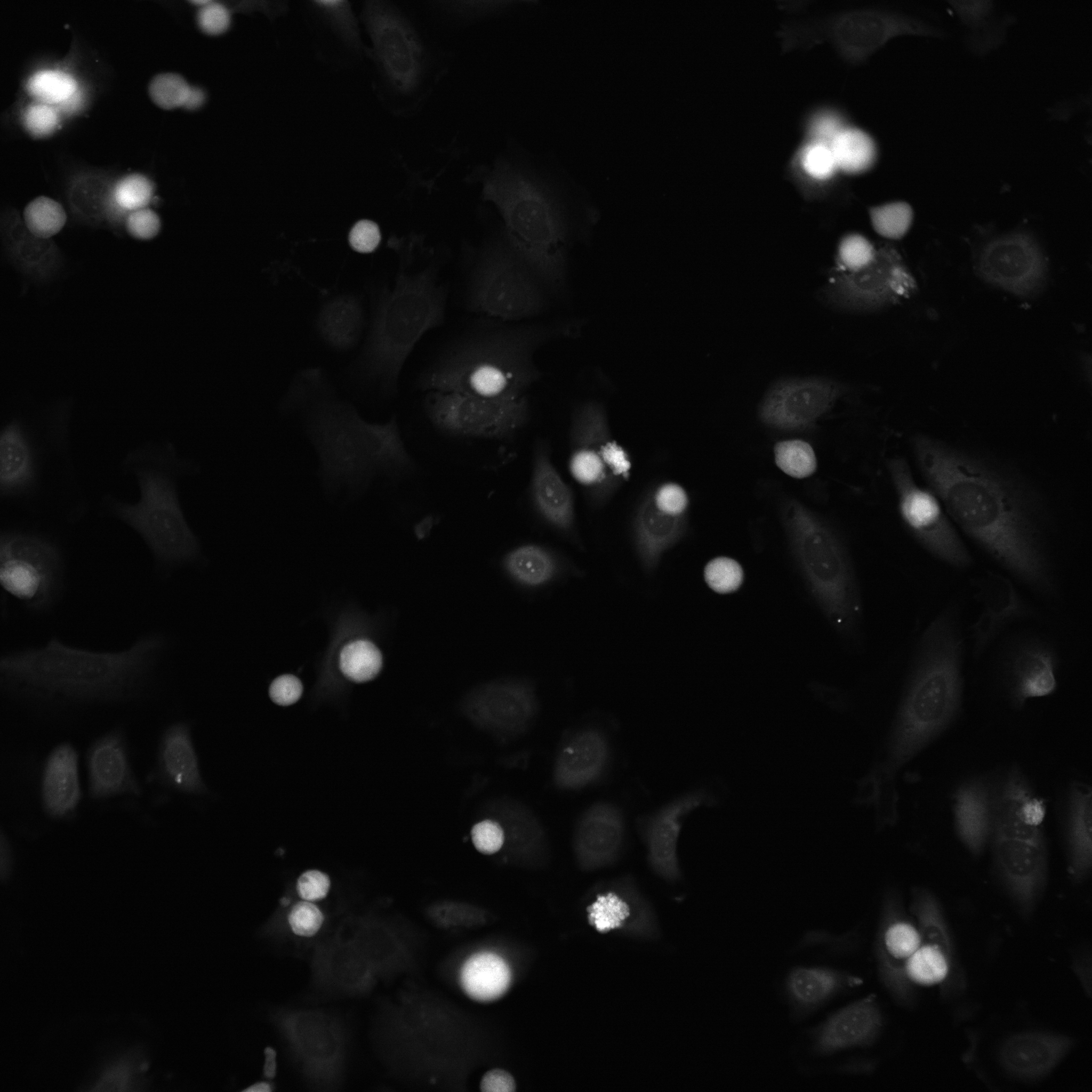

Supplement: Supplementary file 1 — Sample images and results. Sample datasets used in this paper (# 1 and #5 in table 2). The dataset includes input images of both dsRed and Cy5 channels and the corresponding cell segmentation. (ZIP 245,472 kb) [file 12859_2018_2375_MOESM1_ESM.zip › FYVE Hela 1/B - 9(fld 1 wv Green - dsRed).tif]

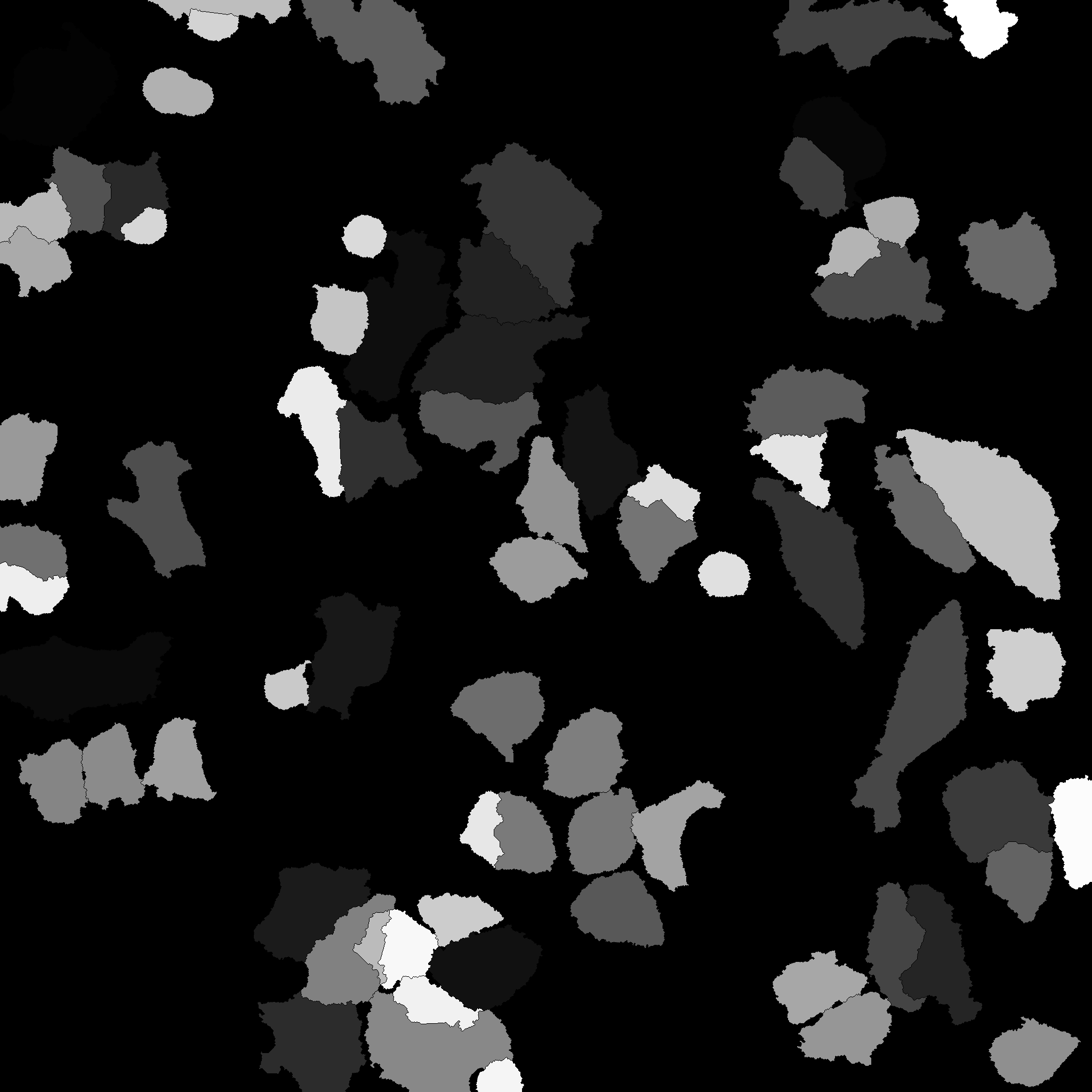

Supplement: Supplementary file 1 — Sample images and results. Sample datasets used in this paper (# 1 and #5 in table 2). The dataset includes input images of both dsRed and Cy5 channels and the corresponding cell segmentation. (ZIP 245,472 kb) [file 12859_2018_2375_MOESM1_ESM.zip › FYVE Hela 1/B - 9(fld 1 wv Green - dsRed)_cellseg_label.tif]

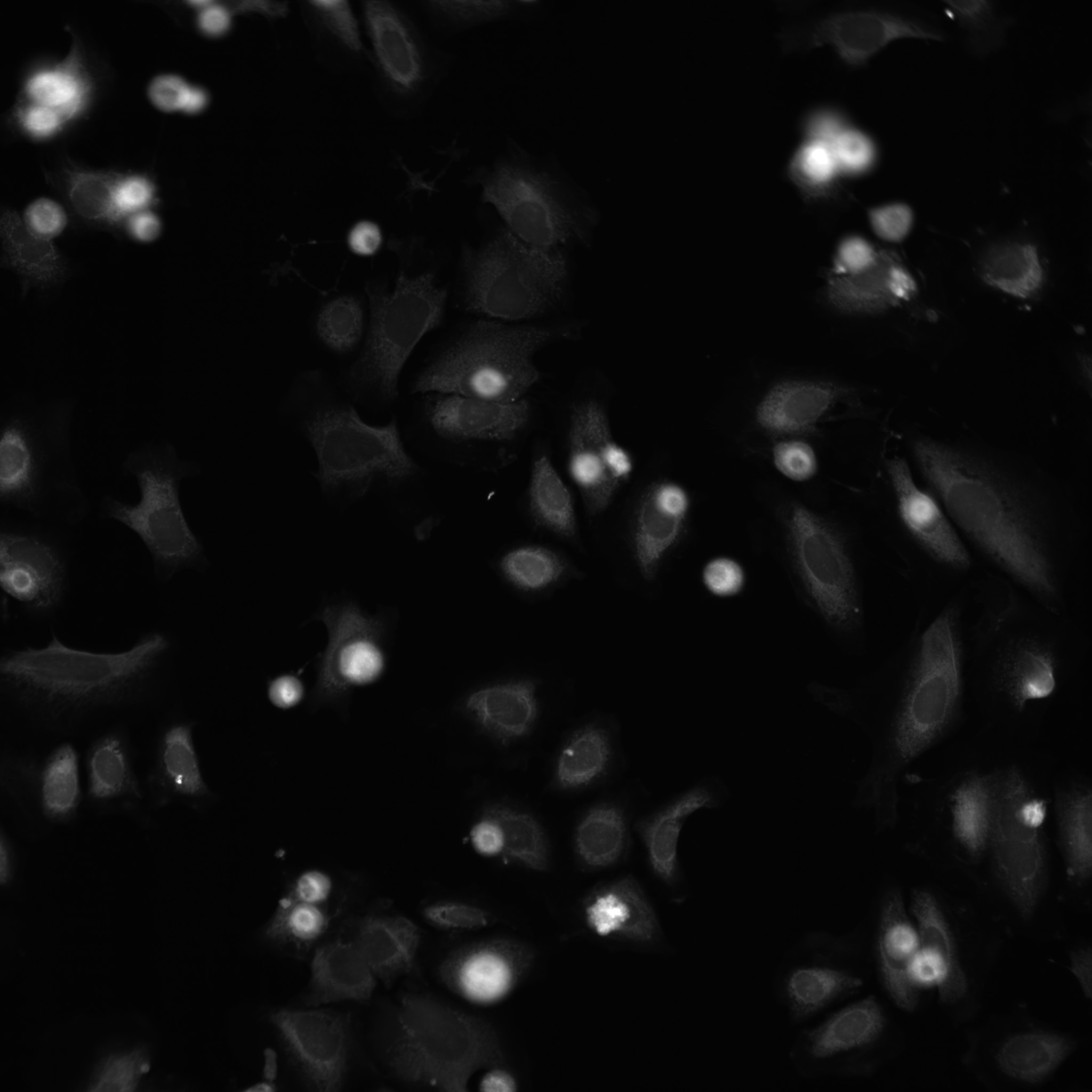

Supplement: Supplementary file 1 — Sample images and results. Sample datasets used in this paper (# 1 and #5 in table 2). The dataset includes input images of both dsRed and Cy5 channels and the corresponding cell segmentation. (ZIP 245,472 kb) [file 12859_2018_2375_MOESM1_ESM.zip › FYVE Hela 1/B - 9(fld 1 wv Red - Cy5).tif]

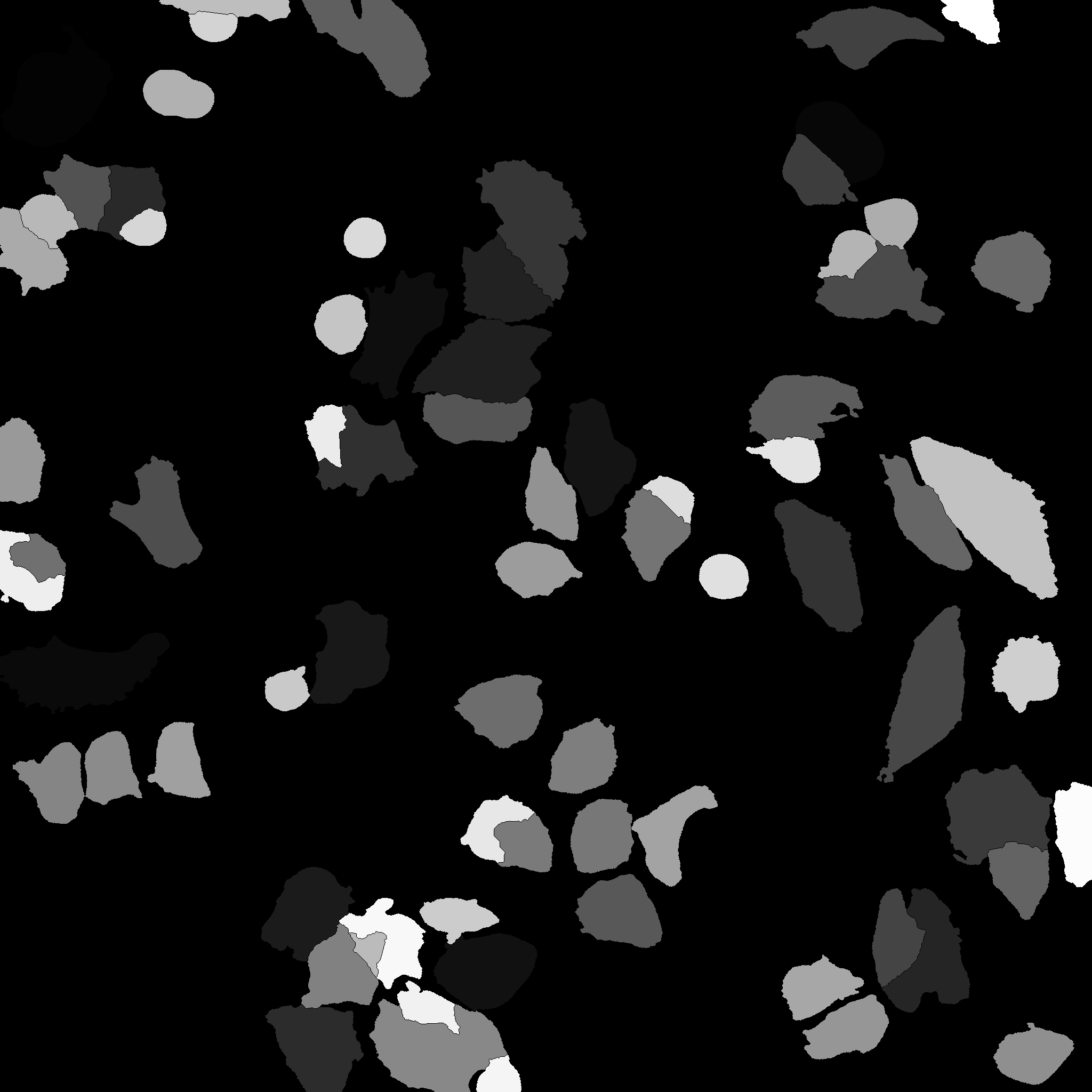

Supplement: Supplementary file 1 — Sample images and results. Sample datasets used in this paper (# 1 and #5 in table 2). The dataset includes input images of both dsRed and Cy5 channels and the corresponding cell segmentation. (ZIP 245,472 kb) [file 12859_2018_2375_MOESM1_ESM.zip › FYVE Hela 1/B - 9(fld 1 wv Red - Cy5)_cellseg_label.tif]
